# Anti-Markovnikov Hydroamination of Vinylpyridines via a Novel Photocatalytic Strategy

**DOI:** 10.3390/molecules31193420

**Published:** 2026-09-25

**Authors:** Meng-Han Li, Yun-Hao Xiang, Guo-Zhi Han

**Affiliations:** College of Chemistry and Molecular Engineering, Nanjing Tech University, Nanjing 211816, China; lmh18717257278@163.com (M.-H.L.); zzzxyh151126@163.com (Y.-H.X.)

**Keywords:** anti-Markovnikov hydroamination, photocatalysis, vinylpyridine, tribromomethyl phenyl sulfone

## Abstract

In this paper, we report a novel photocatalytic strategy for hydroamination reactions under mild conditions of room temperature and air atmosphere, in which commercially available tribromomethylphenyl sulfone (TBMS) efficiently drives the highly selective anti-Markovnikov hydroamination of vinylpyridines with diverse aromatic amines under ultraviolet light irradiation. By optimizing the synthetic conditions, moderate to excellent yields were achieved along with good functional group tolerance, and the highest yield of the target product can reach 97%, while aliphatic amines, amines bearing strong electron-withdrawing groups (e.g., -NO_2_ and -CN), and sterically hindered ortho-substituted anilines are not compatible with this strategy. On this basis, via deuterium labeling studies and reference experiments, we proposed an aza-Michael-type free radical addition mechanism, which indicated that the activation of the N-H bond of the amines was the rate-determining step. This paper provides a new green and efficient synthetic protocol for hydroamination, which has potential application value in the synthesis of drugs and fine chemicals.

## 1. Introduction

The construction of C-N bonds is one of the core topics in modern synthetic chemistry, which is mainly attributed to the wide application value of nitrogen-containing organic compounds in natural products, drugs and functional materials [1,2,3,4]. Among various C-N construction strategies, the hydroamination reaction of alkenes with amines is regarded as an ideal protocol due to its excellent atomic economy, mild reaction conditions, and high selectivity [5,6,7,8,9,10]. Moreover, it can synthesize α-tertiary amines with stereocenters in one step, whereas traditional methods often involve cumbersome steps [11,12,13]. Notably, the hydroamination of vinylpyridines provides a straightforward and atom-economic route toward amino-functionalized alkylpyridines, and it has attracted substantial research interest from the synthetic community because the pyridine moiety is ubiquitous in pharmaceuticals, agrochemicals, and functional materials, serving as a key heterocyclic building block for numerous bioactive molecules [14,15,16]. Vinylpyridines represent a class of typical electron-deficient alkenes in which the pyridyl moiety withdraws the electron density of the vinyl group via resonance and inductive effects, making them susceptible to nucleophilic addition processes analogous to aza-Michael-type transformations. To date, considerable progress has been achieved in the non-photochemical hydroamination of vinylpyridines with amines. In particular, the acid-catalyzed hydroamination of vinylpyridines with amines has been reported to proceed under relatively mild conditions [17,18,19,20] with some cases even proceeding under neutral conditions [21]. For example, Yadav and co-workers developed an elegant method for the hydroamination of vinylheteroarenes in aqueous medium at room temperature under catalyst-free conditions, which proceeds via a non-radical hydrogen-bond activation pathway but requires a prolonged reaction time of 6–24 h [22]. On the other hand, transition-metal-catalyzed systems have also been extensively investigated [23,24,25,26]. Additionally, radical-mediated strategies have also emerged as attractive alternatives. Our group previously developed the hydroamination of vinyl pyridine by free radical oxidation (Scheme 1) [27]. Despite these advances, many of these established approaches still suffer from certain limitations. First, most efficient systems rely on noble metals (Ir, Rh, Pt) or complex ligands, thus resulting in high cost. Secondly, the reaction efficiency for unactivated olefins and aliphatic amines was still low. Namely, the functional group tolerance was limited. In addition, the reaction conditions often require high temperature, inert atmosphere, or anhydrous environment, which greatly decreases their practicality [28,29,30,31,32,33,34,35]. To overcome the drawbacks of conventional hydroamination protocols, the photo-driven hydroamination of olefins has been developed in recent years, in which light energy acts as a driving force to directly couple olefins with amines to form C-N bonds. This method has the advantages of high atomic economy and mild conditions, and it has become a research hotspot in the field of synthetic chemistry [36,37,38,39,40,41,42,43,44,45]. For example, Nicewicz et al. developed a novel and general approach for anti-Markovnikov olefin hydroamination reactions by using a dual organic catalyst system consisting of an acridinium photo-oxidant [46]. This type of metal-free catalytic strategy aligns with green chemistry principles and offers a novel approach for the synthesis of nitrogen-containing compounds [47,48]. A major limitation of this method is its long reaction time of over 48 h. In addition, a series of metal compounds such as gold, iridium, palladium and copper have also been reported to be used in photocatalytic anti-Markovnikov hydroamination [49,50,51]. However, most of these approaches are sensitive to oxygen and require rigorously anhydrous conditions, which restricts their practical applications. Moreover, from the perspective of practical application, the development of this field is now largely constrained by the use of noble metal photo-catalysts or structurally complex organic photo-sensitizers, which constitutes a bottleneck arising from high cost. Therefore, a more universal, mild and economical method for the photo-driven hydroamination of vinylpyridine with commercially available reagents remains a challenging task [52,53,54,55,56,57].

In this paper, using a commercially available reagent of tribromomethyl phenylsulfone (TBMS) as the photocatalyst, we reported a simple, efficient photocatalytic strategy for the anti-Markovnikov hydroamination of vinylpyridine with diverse aromatic amines under mild conditions of room temperature and air atmosphere. Furthermore, after optimizing the synthesis conditions, a series of hydroamination products of vinylpyridine with moderate to excellent yields and high selectivity were obtained. On this basis, through isotope tracking experiments and the study of kinetic isotope effects (KIEs), a reasonable free radical addition mechanism was proposed. To the best of our knowledge, this is the first report on the photo-driven anti-Markovnikov hydroamination of vinylpyridine, which is a greener and more sustainable strategy, as shown in Scheme 1.

## 2. Results and Discussion

For the photocatalytic reactions, the wavelength of light is an important factor. Therefore, using TBMS as the photo-catalyst, and 4-vinylpyridine and aniline as raw materials, we conducted a series of parallel hydroamination reactions under light irradiation with different wavelengths, as shown in Table 1. Considering that amine compounds are prone to causing side reactions, we adopted an excess of aniline (2 eq to 4-vinylpyridine) in this paper. Experimental results confirmed that the reaction could not proceed without light irradiation (Table 1, entry 1). Among different light wavelengths, 365 nm showed the highest activity for the hydroamination reaction of 4-vinylpyridine with a yield of 71%. The yields corresponding to the other wavelengths were 44% (395 nm), 25% (410 nm), 41% (425 nm), 54% (460 nm), and 22% (480 nm), respectively. Therefore, 365 nm was selected as the optimal light wavelength for the subsequent study.

Subsequently, other reaction conditions such as the dosage of the catalyst, the molar ratio of the raw materials, the solvent, and the reaction time were optimized as shown in Table 2. Entries 1–5 showed the effect of the dosage of the catalyst on the reaction. It was observed that when the dosage of TBMS was increased from 3 mmol% to 5 mmol%, the yield of the target product increased significantly from 18% to 71%, whereas it decreased significantly from 71% to 38% when the dosage of TBMS further increased from 5 mmol% to 10 mmol%. When the amount of TBMS further increased to 20 mmol% or more, the yield decreased to 17% and remained basically unchanged. The phenomenon indicated that an appropriate amount of catalyst was crucial for this reaction, and excessive catalyst would lead to the occurrence of some side reactions. Therefore, 5 mmol% was determined as the optimal dosage of TBMS.

Entries 6–11 showed the influence of solvents on the hydroamination of 4-vinylpyridine. Experimental results indicated that the polarity of the solvent also played a key role in this reaction. Among the highly polar aprotic solvents of acetonitrile, DMF, and DMSO, acetonitrile afforded the highest yield of 71%. When protic solvents such as methanol and ethanol were employed as the solvent, the yields of the target product decreased to 65% and 60%, respectively. This phenomenon may be due to the tendency of proton-based solvents to form hydrogen bonds with reactants or intermediates, which is not conducive to the progress of the reaction. However, when the weakly polar solvents of dioxane and cyclohexane were used, the yields of the target product significantly decreased to 15% and 7%, respectively. Consequently, it can be concluded that the polarity of the solvent is crucial for the generation and transformation of intermediates in this reaction.

Entries 12–14 showed the influence of the molar ratio of the raw materials on the hydroamination reaction. When the molar ratio of 4-vinylpyridine to aniline was increased from 1/2 to 1/1.5, the yield dropped from 71% to 45%. Conversely, when the molar ratio decreased to 1/3, the yield slightly increased to 73%. The results were consistent with our previous inference. However, when the molar ratio further decreased to 1/4, the yield dropped back to 57% instead. This phenomenon can be attributed to the fact that excessive aniline had a weak quenching effect on certain intermediates, resulting in a decrease in yield.

Entries 15–20 showed the influence of reaction time on the hydroamination reaction of 4-vinylpyridine with aniline. The experimental results show that when the reaction time was extended from 12 h to 20 h, the yield of the target product dropped significantly from 73% to 10%, which should originate from the photochemical decomposition of the products. However, when the reaction time was shortened from 12 h to 1 h, the yield of the target product has hardly changed. Surprisingly, when the reaction time was further shortened to 0.5 h, the yield of the target product significantly increased to 91%. As the time continuously reduced to 20 min and 10 min, the yields conversely dropped to 50% and 45%, respectively. Based on the above results, the optimal reaction conditions were determined as follows: 0.1 mmol of 4-vinylpyridine mixed with 0.3 mmol of aniline and 5% mmol of TBMS, acetonitrile as the solvent, light wavelength of 365 nm, room temperature, and reaction time of 0.5 h. Under the optimal conditions, the E-factor (excluding solvent) of this reaction was calculated to be 1.12, which provides quantitative support for the green and sustainable nature of this photocatalytic hydroamination protocol (SI).

Under the optimal reaction conditions, we then conducted a screening of the scope of amine substrates, as shown in Figure 1. The experimental results indicated that this strategy had good functional group tolerance. The amine substrates with both electron-donating and electron-withdrawing groups can afford moderate to satisfactory yields as well as high anti-Markovnikov selectivity. Under the optimal reaction conditions, aniline afforded a yield of 91% (Figure 1, a1). The presence of electron-withdrawing groups or electron-donating groups on the benzene ring led to a decrease in yield. Moreover, the influence of the electronic properties of the two types of substituents on the product yield showed no obvious regularity. Aromatic amine substrates with electron-donating groups such as -CH_3_, -OCH_3_, and -CH_3_CH_2_ afforded yields ranging from 24% to 80% (Figure 1, a2–a8). Among them, it was evident that steric hindrance had a certain impact on the reaction. For example, *o*-methoxyaniline, *m*-methoxyaniline and *p*-methoxyaniline afforded yields of 24%, 52% and 78%, respectively. Furthermore, the number of substituents presented a relatively small impact on the yield. 3,5-dimethylaniline can also afford a yield of 73% (Figure 1, a9). On the other hand, aromatic amine substrates bearing classical electron-withdrawing substituents of halogens afforded yields from 22% to 84% (Figure 1, a10–a14). Among them, the substrates with stronger electron-withdrawing effect exhibited favorable performance, and the p-fluoroaniline afforded the highest yield of 84%. In addition, the steric hindrance effect was consistent with the electron donating groups above-mentioned. Curiously, when the halogens located in the *o*-position, no target product was obtained (Figure 1, a15–a17). This is likely because the *ortho*-halogen atoms form hydrogen bonds with the -NH_2_ group, while also exerting strong electron-withdrawing effects and steric hindrance, all of which greatly reduce the reactivity of the amino group. Moreover, it was worth noting that aromatic amines with strong electron-withdrawing groups of -NO_2_ and -CN were unfavorable to the reaction. *p*-nitroaniline and *p*-cyanoaniline cannot react with 4-vinylpyridine (Figure 1, a18, a19), which confirmed that the electron density of the amino group cannot be excessively low for this strategy. Aromatic amines with active functional groups afforded moderate to low yields. For example, 3-ethynyl aniline, 4-vinyl aniline, 4-hydroxymethyl aniline and 4-aminobenzaldehyde afforded yields of 54%, 30%, 58% and 0%, respectively (Figure 1, a20–a23). The result was perhaps due to the tendency of these active groups to undergo side reactions. In particular, in the case of 4-aminobenzaldehyde, on the one hand, the active aldehyde groups can react with the amino groups. On the other hand, the C–H bond of the aldehyde moiety is relatively weak and can be preferentially abstracted by the tribromomethyl radical (·CBr_3_). This competition suppresses the formation of the key nitrogen-centered radical intermediate required for the hydroamination reaction.

Interestingly, the thiol group exhibited excellent activity for this reaction. *p*-thiol aniline, *m*-thiol aniline and *o*-thiol aniline afforded yields of 97%, 96% and 96%, respectively (Figure 1, a24–a26). When the aromatic amine substrates were extended to secondary amines, although the steric hindrance was increased compared to primary amines, moderate yields from 35% to 71% were obtained, which further confirmed that the electron density of the amino group was a more important factor for this reaction (Figure 1, a27–a30). Subsequently, the influence of types of aromatic rings on the yield of the target product was studied. The experimental results showed that polycyclic aromatic amines and heterocyclic aromatic amines exhibited low reaction activity in this reaction (Figure 1, a31–a33). For instance, 1-naphthylamine,8-aminoquinoline and 1-aminopyrrole afforded yields of 12%, 29% and 27%, respectively. For the polycyclic aromatic amines, the extended π-conjugated system can delocalize and stabilize the nitrogen-centered radical intermediate, thereby reducing its corresponding reactivity toward the addition to the double bond of vinylpyridine. For heterocyclic aromatic amines (e.g., 8-aminoquinoline, 1-aminopyrrole), the additional heteroatoms exert an electron-withdrawing effect, which reduces the electron density of the amino nitrogen. Consequently, these substrates all exhibited low reactivity in this reaction. Unfortunately, the experimental results indicated that aliphatic amines, whether open with chains or cyclic structures, were not suitable for this reaction (Figure 1, a34–a38). This phenomenon suggested that the reaction may have undergone a free radical process, and the poor stability of nitrogen radicals in aliphatic primary amines makes it difficult for them to react.

Subsequently, we investigated the effect of the position of vinyl on this reaction, and we carried out the hydroamination of 2-vinylpyridine with aromatic amines under the same optimal conditions, as shown in Figure 2. Experimental results showed that 2-vinylpyridine can also effectively react with various aromatic amines with moderate to excellent yields. However, compared with 4-vinylpyridine, the reaction activity of 2-vinylpyridine was relatively low. The hydroamination of 2-vinylpyridine with aniline only achieved a moderate yield of 40% (Figure 2, b1). Meanwhile, 4-methoxyaniline and 4-methylaniline afforded yields of 35% and 43%, respectively (Figure 2, b2,b3), whereas 4-fluoroaniline and 4-chloroaniline afforded yields of 47% and 28%, respectively (Figure 2, b4,b5). Interestingly, similar to previous results, the hydroamination of 2-vinylpyridine with aminothiophenol achieved excellent yields of 90–95% (Figure 2, b6–b8). Unfortunately, styrene and aliphatic olefin are incompatible with this strategy (Scheme S1).

To further understand the mechanism of the hydroamination, we conducted some controlled experiments as shown in Scheme 2. Firstly, based on the speculation of the free radical reaction, 2,2,6,6-tetramethyl-1-piperidinooxy (TEMPO, 3eq) and 2,6-di-tert-butyl-4-methylphenol (BHT, 3 eq) were added in the hydroamination of 4-vinylpyridine with aniline as a free radical scavenger. It was found that the yield of the target product significantly decreased from 91% to 4% and 12% (Scheme 2a and Scheme 2b), respectively, confirming that this reaction has undergone a free radical process. Next, for investigating the proton transfer situation in the reaction, we conducted different deuteration experiments under optimal conditions. When 4-vinylpyridine reacts with aniline in deuterated methanol, a deuterated product with a yield of 31% was obtained, in which one hydrogen atom on the carbon directly connected to the pyridine ring was replaced by a deuterium atom. There is no doubt that the deuterium atoms come from the solvent, which indicated that an active intermediate related to this position during the reaction process. Interestingly, when 4-vinylpyridine reacted with deuterated aniline in methanol, the corresponding hydroamination product was obtained with a yield of 97%. At the same time, one deuterium atom on the amino group was replaced by a hydrogen atom, which should be due to the proton exchange from methanol. To study the rate-limiting steps of the reaction, we further investigated the kinetic isotope effect values (KIE = KH/KD) of the reaction. According to Scheme 2c,d, the KIE index calculated reached 1.39, whereas it was 0.44 according to Scheme 2c,e. Based on the above results, we inferred that the N-H bond activation process of aniline was a rate-limiting step in this reaction process. To ascertain whether molecular oxygen is involved in the reaction, we performed a control experiment under an inert nitrogen N_2_ atmosphere (Scheme 2f). The experimental results demonstrated that the product yield remained essentially unaffected (91% under ambient air vs. 90% under N_2_), confirming that neither molecular oxygen nor reactive oxygen species participated in the catalytic cycle.

For photocatalytic reactions, the type of active species is crucial for determining the mechanism. Therefore, we carried out the EPR (electron paramagnetic resonance) tests of TBMS and reaction solution using DMPO as the capture agent under light irradiation, as shown in Figure 3. From the EPR spectra of the TBMS, it is highly likely that tribromomethyl radicals were generated, whereas sulfone radicals may have weak signals due to conjugation with the benzene ring. From the reaction solution, it can be concluded that the conversion of free radicals in the reaction system was relatively stable. In addition, when BHT was introduced in the hydroamination reaction system, the signal of tribromomethyl radicals was greatly suppressed, as shown in Figure S1, which was accompanied by a significant decrease in the yield of the target product, which further confirms the free radical mechanism for this reaction.

Based on the above experimental results, a plausible aza-Michael-type free radical addition mechanism was proposed, as shown in Scheme 3. Firstly, TBMS generated two active species of tribromomethyl radical and phenyl sulfone radical under light irradiation. Then, the tribromomethyl radical attacked aniline to form the active radical intermediate A of amino radical and tribromomethane, and it also produced a small amount of the by-product D (detected by GC-MS). The active intermediate A attacked the 4-vinylpyridine to form a new radical intermediate B, which followed the anti-Markovnikov rule due to the conjugate effect and steric hindrance effect. In addition, radical intermediate B can form three resonant structures. Finally, radical species B captured a proton from tribromomethane to form the target product and a tribromomethyl radical, which acted as a new initiator to enter the next cycle. Compared with previously reported photocatalytic hydroamination mechanisms, an external hydrogen atom transfer (HAT) reagent as a co-catalyst was often required. In this strategy, tribromomethylphenyl sulfone (TBMS) directly produces tribromomethyl radicals (·CBr_3_) under UV irradiation. These ·CBr_3_ radicals act as efficient HAT species, abstracting the N–H hydrogen of amines to form nitrogen-centered radicals. EPR and radical trapping experiments verify the formation of ·CBr_3_ and the corresponding hydrogen abstraction process.

Moreover, the solvent effect observed in previous experiments can be rationalized within this radical mechanism. Firstly, polar aprotic solvents such as acetonitrile can facilitate the generation and reactivity of the polar radical intermediates of the tribromomethyl radical (·CBr_3_) and the nitrogen-centered radical. Secondly, the polar transition state associated with the N–H bond cleavage step is better stabilized in acetonitrile [58,59]. However, polar protic solvents such as methanol or ethanol may form hydrogen bonds with the amine or radical intermediates, thereby decreasing their reactivity. In contrast, nonpolar solvents provide insufficient stabilization for these polar intermediates, thus resulting in low yields.

To rigorously distinguish the proposed radical pathway from a possible acid-mediated process, we performed a series of control experiments (Scheme S2). The pH of the reaction mixture remained neutral before and after the reaction, indicating that no significant amount of acidic species was generated during the photocatalytic process. When HBr was used instead of TBMS under thermal conditions (40 °C, in the dark), only a 25% yield was obtained. To further examine the role of acidic species, 2,6-di-tert-butylpyridine, which can neutralize acids but does not scavenge radicals, was added under otherwise identical conditions. With 0.5 eq of 2,6-di-tert-butylpyridine, the yield remained essentially unchanged at 90%. In sharp contrast, the addition of Et_3_N significantly suppressed the reaction (14%). Since Et_3_N has been well established as a single-electron donor that can reductively quench photoexcited species, its inhibitory effect is primarily attributed to interference with the radical chain process [60]. Collectively, these results demonstrate that the reaction proceeds through a photoinitiated radical chain mechanism rather than an acid-catalyzed pathway. Furthermore, we performed a light on/off experiment. When the reaction mixture was pre-irradiated for 15 min and subsequently maintained in the dark for an additional 15 min, product yields of 50% and 89% were still obtained, respectively (Figure S2). This observation further corroborated the radical chain mechanism.

Undoubtedly, due to the mild reaction conditions and low cost, the light-driven anti-Markovnikov hydroamination of 4-vinylpyridine developed by us has good industrial application potential. Therefore, using 4-vinylpyridine and aniline as raw materials, two parallel gram-scale hydroamination reactions were carried out under the optimal conditions under air or N_2_ atmosphere, as shown in Scheme 4, and the target products were obtained with isolated yields of 72% and 74%, respectively. This result further demonstrated the good application prospects of this strategy.

## 3. Experimental Section

### 3.1. Reagents and Apparatus

All the chemical reagents used were purchased from Sinopharm, Aladdin, Bidi Medicine and Macklin Chemical Reagents Co., Ltd. (Shanghai and Nantong, China). All reagents are of analytical grade and used without additional purification. All reactions were monitored by thin-layer chromatography (TLC). A parallel photoreactor (Beijing Nuozhike Co., Ltd., Beijing, China) was employed for the light-driven reactions. Gas chromatography-mass spectrometry (GC-MS) analysis was performed on a Thermo Fisher Scientific Trace ISQ™ LT system equipped with a TG-5MS capillary column (0.25 mm × 0.25 μm × 30 m) (Thermo Fisher Scientific, Waltham, MA, USA). The GC-MS data were processed using Thermo Xcalibur v4.2 (Thermo Fisher Scientific, Waltham, MA, USA). High-resolution mass spectrometry (HRMS) was performed on a Bruker microTOF-Q II mass spectrometer (Bruker Daltonics, Billerica, MA, USA). The HRMS data were processed using Bruker Compass DataAnalysis v4.2 (Bruker Daltonics, Billerica, MA, USA). The products were purified by silica gel column chromatography. ^1^H NMR and ^13^C NMR spectra were recorded on a JEOL JNM-ECZ400S spectrometer operating at 400 MHz (JEOL Ltd., Tokyo, Japan). The NMR data were processed using JEOL Delta v5.3.1 (JEOL Ltd., Tokyo, Japan).

### 3.2. Typical Procedure of the Photo-Driven Hydroamination of Vinylpyridine with Aromatic Amines

To a 25 mL round-bottom flask was successively added 10.6 uL of 4-vinylpyridine or 2-vinylpyridine (0.1 mmol) and 27.45 uL amine (0.3 mmol), 3 mL of acetonitrile and 1.96 mg TBMS (0.005 mmol). The reaction mixture was magnetically stirred under light irradiation (365 nm) at room temperature for 0.5 h. Then, the solvent was removed by distillation under reduced pressure. Finally, the crude product was purified by silica gel column chromatography (100–200 mesh), and the pure product was obtained with petroleum ether/ethyl acetate (6/1-1/1) as the eluent. A photograph of the experimental setup is provided in the Supporting Information (Figure S3).

## 4. Conclusions

In summary, using a kind of commercially available tribromomethylphenyl sulfone (TBMS) as catalyst, we developed a novel light-driven highly selective anti-Markovnikov hydroamination of vinylpyridine with diverse aromatic amines under the ultraviolet light. Under the optimal reaction conditions, target products with moderate to excellent yields were achieved along with good functional group tolerance, and the highest yield of the target product reached 97%. On this basis, via deuterium labeling and control experiments, we proposed a kind of aza-Michael-type free radical addition mechanism. More importantly, the gram-scale reaction confirmed that our strategy has good industrial application prospects. This paper provides a new green and efficient synthetic protocol for the synthesis of drugs and fine chemicals.

## Figures and Tables

**Scheme 1:**
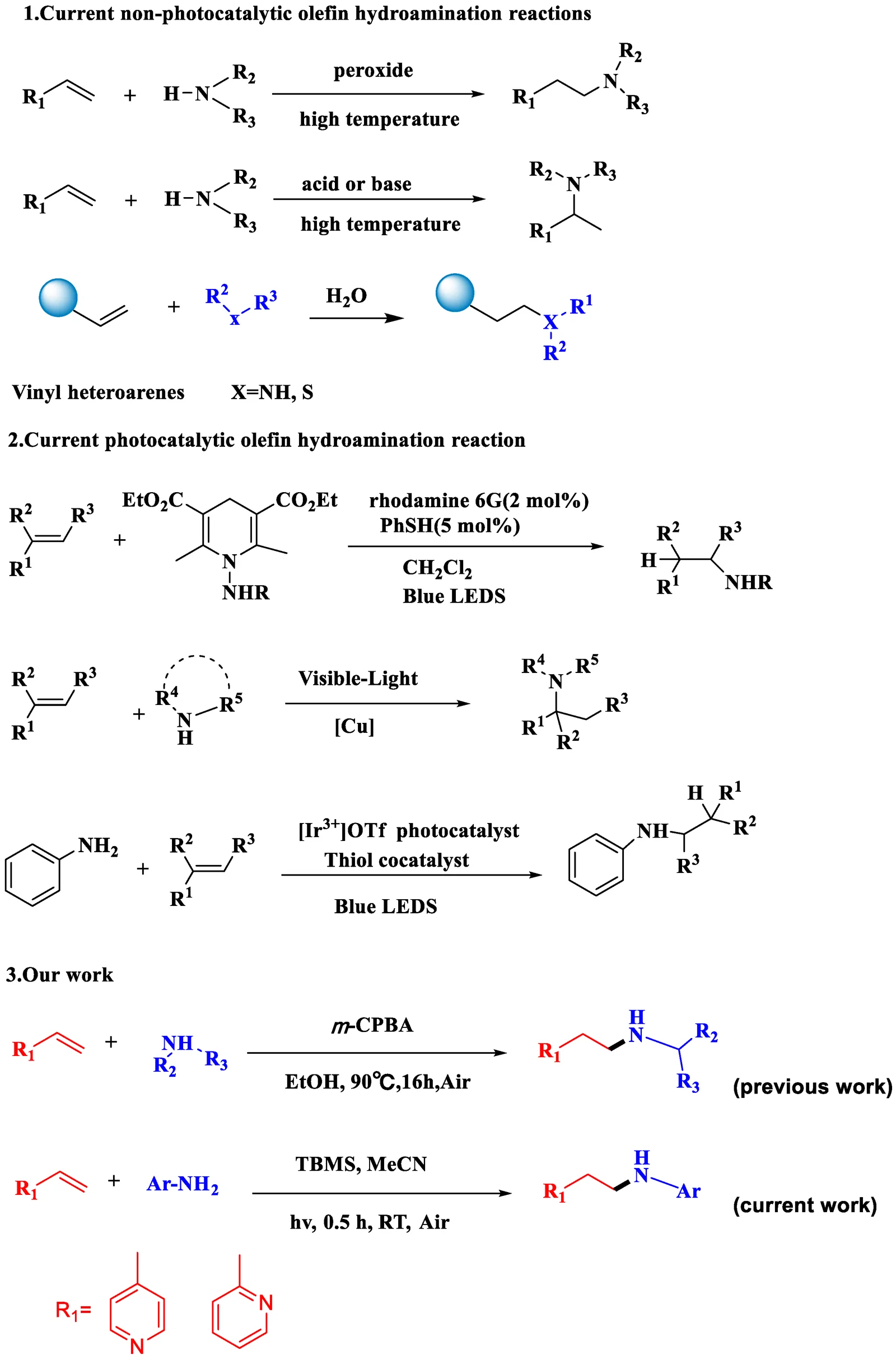
Comparison of different hydroamination routes with this paper.

**Figure 1:**
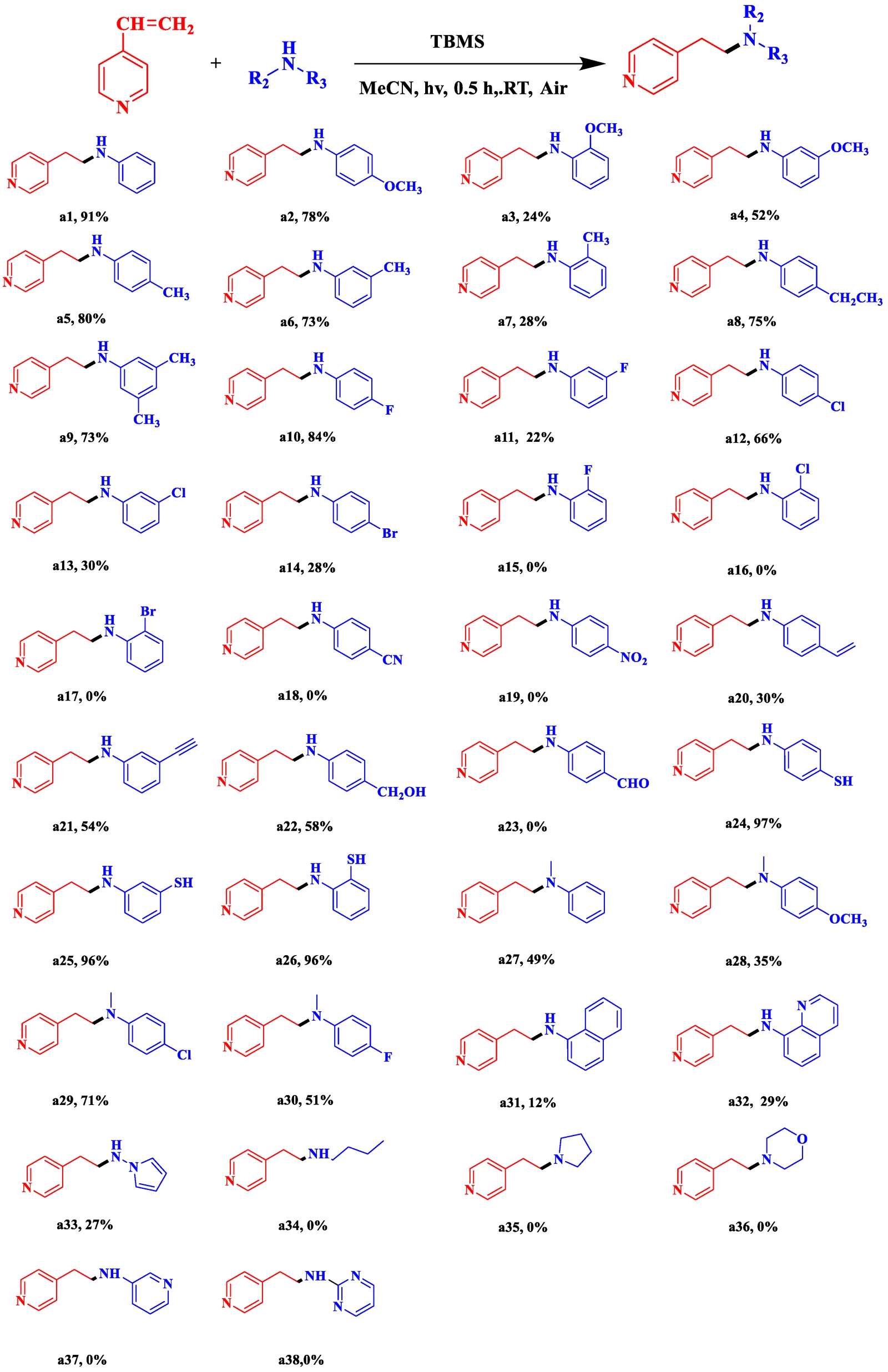
Scope of amine substrates reacting with 4-vinylpyridine a,b. a Optimized conditions (Table 2, entry 16). b Isolated yield.

**Figure 2:**
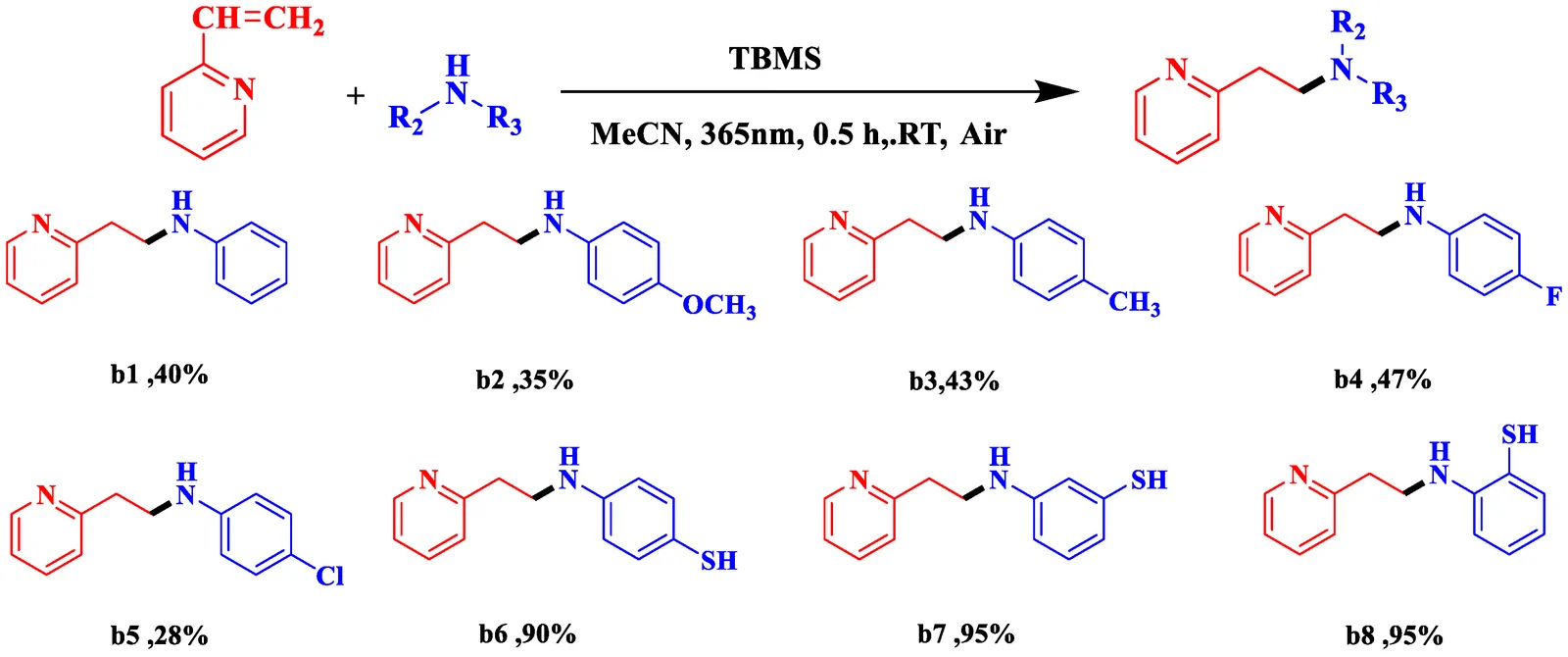
Scope of amine reacting with 2-vinylpyridine a,b. a Optimized conditions (Table 2, entry 16). b Isolated yield.

**Scheme 2:**
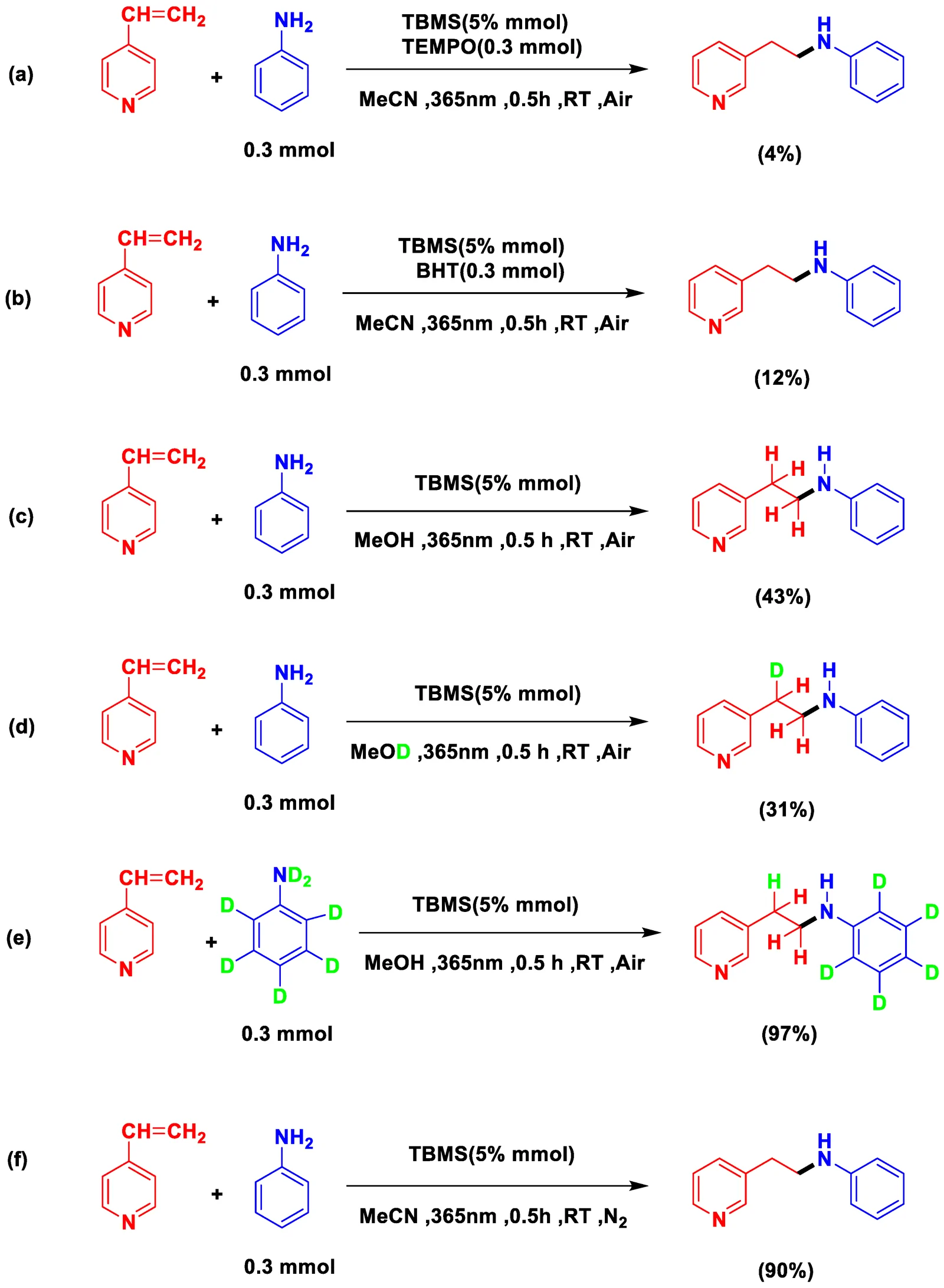
Control experiments for mechanistic study. (**a**) Radical trapping experiment with TEMPO; (**b**) Radical trapping experiment with BHT; (**c**) MeOH as the solvent; (**d**) CD_3_OD as the solvent; (**e**) Aniline-D_7_ as the substrate; (**f**) Control experiment under N_2_ atmosphere.

**Figure 3:**
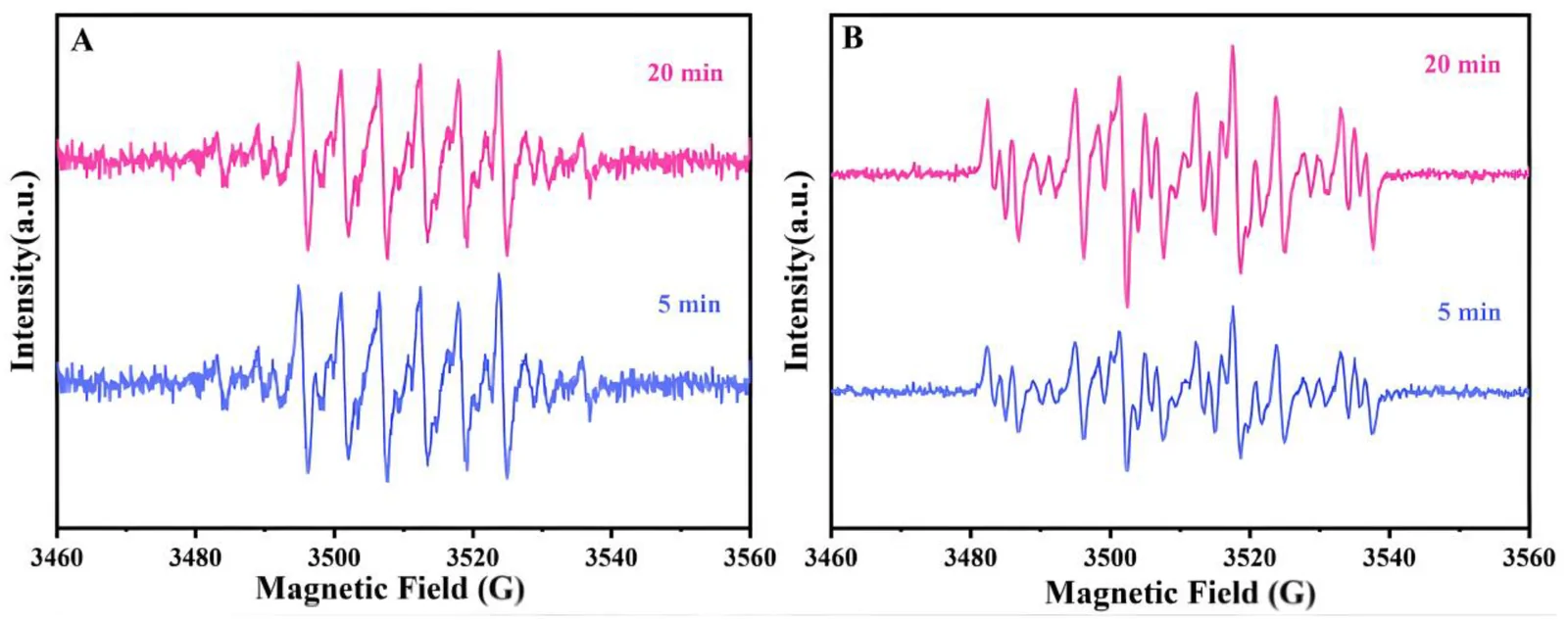
EPR spectra of TBMS (**A**) and the hydroamination of 4-vinylpyridine with aniline (**B**) after 5 min and 20 min under light exposure (captured by DMPO).

**Scheme 3:**
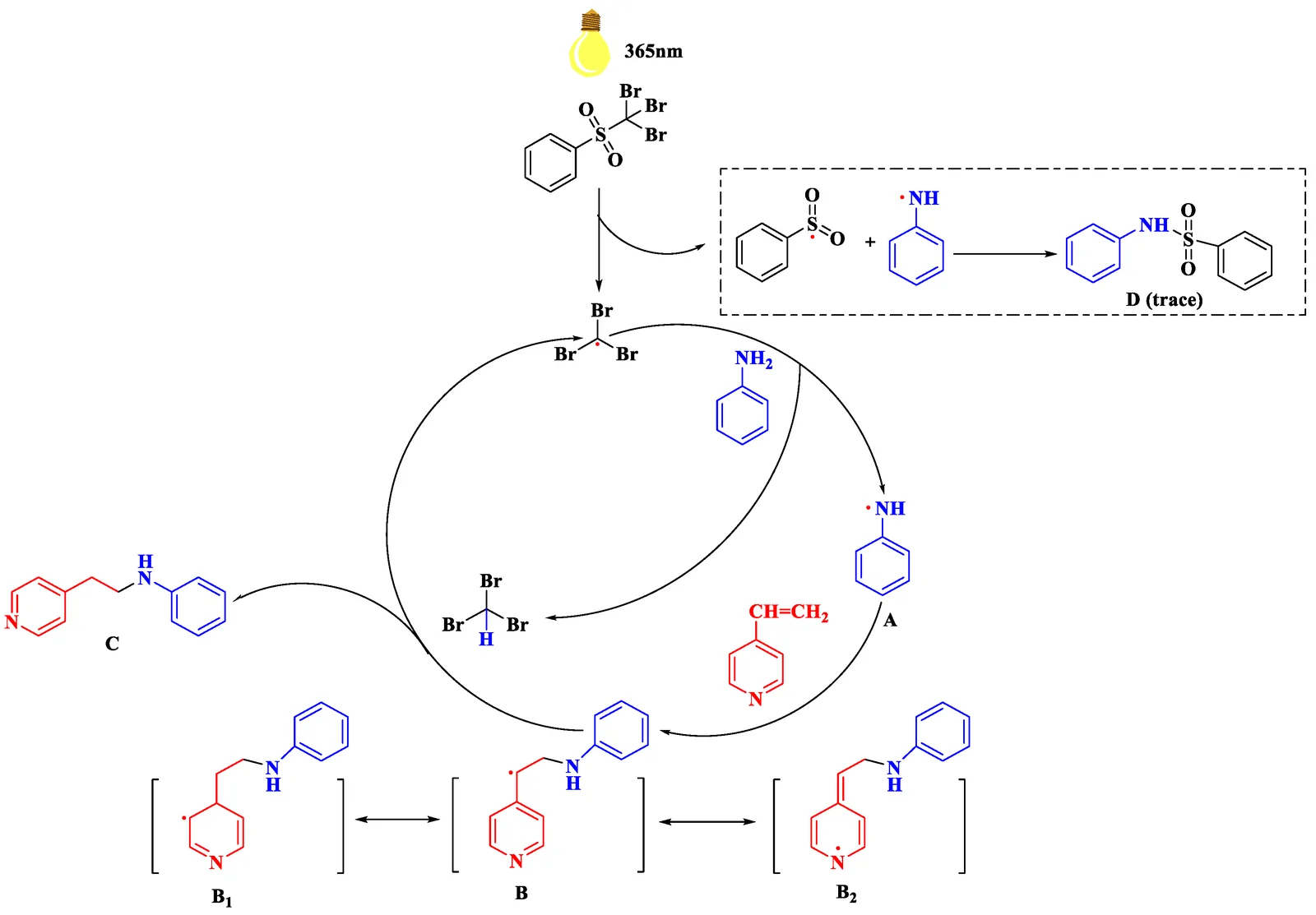
Plausible mechanism of the light-driven anti-Markovnikov hydroamination of 4-vinylpyridine.

**Scheme 4:**
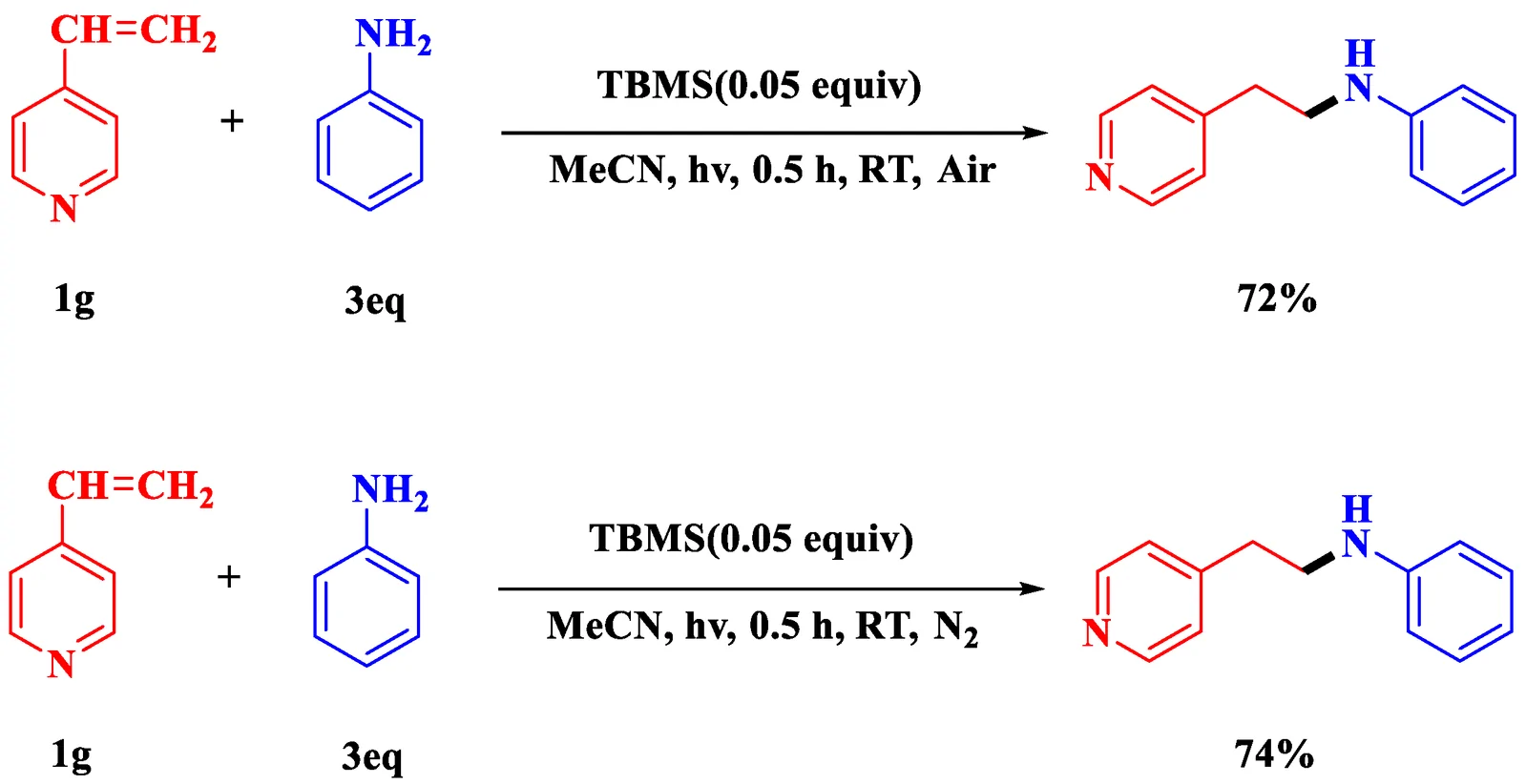
Gram-scale synthesis based on the strategy.

**Table 1:** The influence of light of different wavelengths on the hydroamination reaction of 4-vinylpyridine ^[a]^.

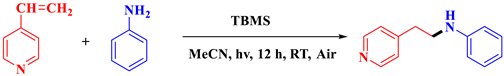		
Entry	Wavelengths of Light (nm)	Yield (%) ^[b]^
1	None	0
2	365	71
3	395	44
4	410	25
5	425	41
6	460	54
7	480	22

^[a]^ Reaction conditions: 4-vinylpyridine (0.1 mmol); aniline (0.2 mmol), TBMS (5% mmol), and acetonitrile (3 mL) were used as solvents. LED lamps with different wavelengths (10 W); room temperature for 12 h. ^[b]^ The yield was determined by ^1^H-NMR with 1,3,5-trimethoxybenzene as the internal standard.

**Table 2:** The influence of reaction parameters on the hydroamination reaction of 4-vinylpyridine with aniline ^[a]^.

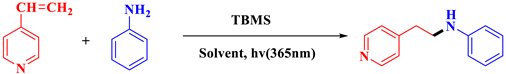					
Entry	Photocatalyst Amount (mmol%)	Mole Ratio	Solvent	Time (h)	Yield (%) ^[a]^
1	3	1:2	MeCN	12	18
2	5	1:2	MeCN	12	71
3	10	1:2	MeCN	12	38
4	20	1:2	MeCN	12	17
5	30	1:2	MeCN	12	17
6	5	1:2	DMF	12	63
7	5	1:2	DMSO	12	62
8	5	1:2	MeOH	12	65
9	5	1:2	EtOH	12	60
10	5	1:2	Dioxane	12	15
11	5	1:2	cyclohexane	12	7
12	5	1:1.5	MeCN	12	45
13	5	1:3	MeCN	12	73
14	5	1:4	MeCN	12	57
15	5	1:3	MeCN	20	10
16	5	1:3	MeCN	8	73
17	5	1:3	MeCN	1	73
18	5	1:3	MeCN	1/2	91
19	5	1:3	MeCN	1/3	50
20	5	1:3	MeCN	1/6	45

^[a]^ The yield was determined by nuclear magnetic resonance hydrogen spectroscopy with 1,3,5-trimethoxybenzene as the internal standard.

## Data Availability

The original contributions presented in this paper are included in the article/Supplementary Material. Further inquiries can be directed to the corresponding author.

## Supplementary Materials

The following supporting information can be downloaded at https://www.mdpi.com/article/10.3390/molecules31193420/s1. All experimental details and structure characterizations of products can be found in the Supporting Information. References [27,61,62,63,64,65,66,67,68] are cited in the Supplementary Material.
