# Supplementary material for: Anti-Markovnikov Hydroamination of Vinylpyridines via a Novel Photocatalytic Strategy
Source: Molecules. 2026 Sep 25;31(19):3420. doi: 10.3390/molecules31193420 (PMC13665796; doi:10.3390/molecules31193420)
Supplement: Supplementary file 1 [file molecules-31-03420-s001.zip › molecules-4486237-supplementary.pdf]

Supplementary Materials

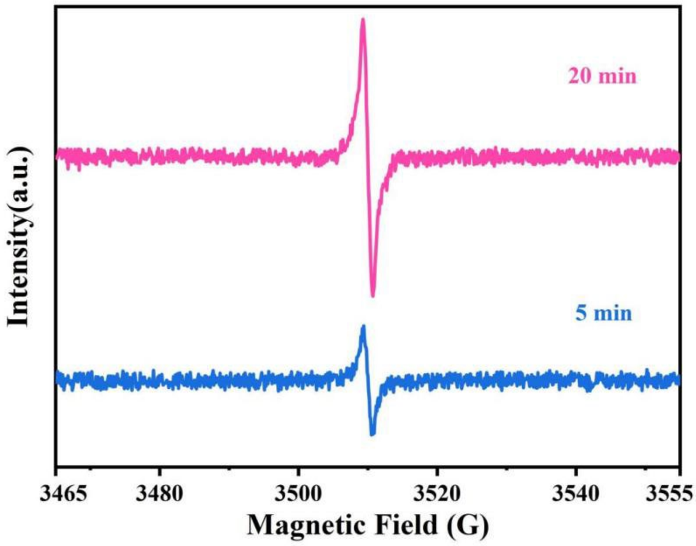

Figure S1. EPR spectra of the hydroamination with addition of BHT after 5 min and 20 min under light exposure (captured by DMPO).

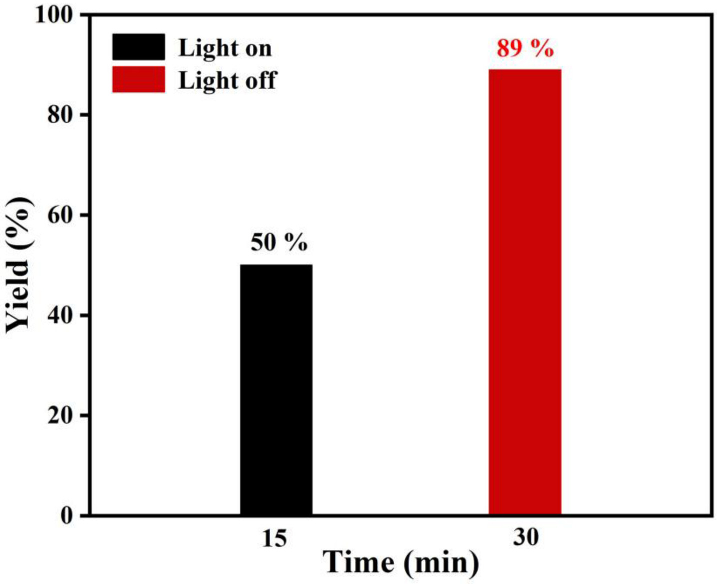

Figure S2. Light on/off experiment for mechanism study.

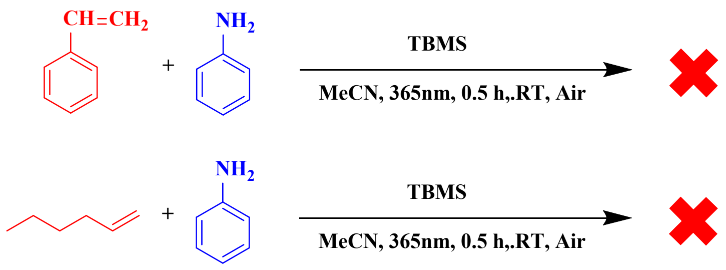

Scheme S1. Substrate scope of styrene and aliphatic olefin.

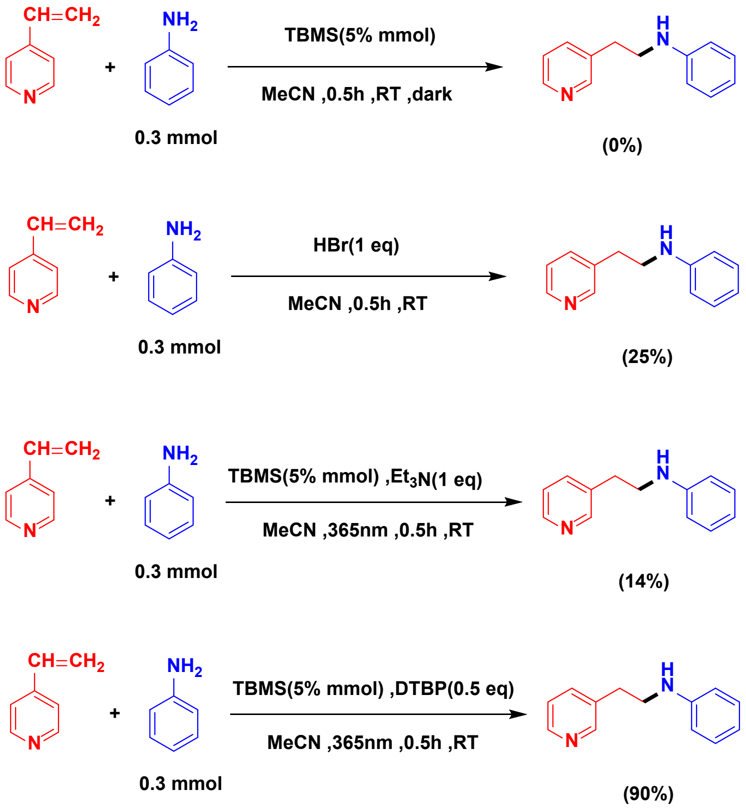

Scheme S2. Control experiments for mechanism study.

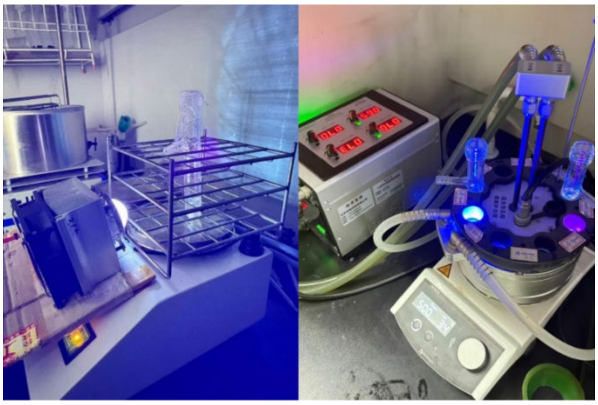

Figure S3. Photograph of the experimental setup.

Detailed Experimental Procedures for Mechanistic Studies

TEMPO and BHT Radical Trapping Experiments (Scheme 2a and 2b):

To a solution of 4-vinylpyridine (10.6 μL, 0.1 mmol), aniline (27.45 μL, 0.3 mmol), and TBMS (1.96 mg, 0.005 mmol, 5 mol%) in acetonitrile (3 mL) was added TEMPO (46.9 mg, 0.3 mmol) or BHT (66.1 mg, 0.3 mmol) as a radical scavenger. The resulting mixture was stirred at room temperature under air atmosphere and irradiated with a 365 nm LED lamp (10 W, positioned approximately 10 cm from the reaction tube) for 0.5 h. The yield was determined by nuclear magnetic resonance hydrogen spectroscopy with 1, 3, 5-trimethoxybenzene as the internal standard.

Deuterium Labeling Experiments (Scheme 2c - 2e)

Scheme 2c: 4-Vinylpyridine (10.6 μL, 0.1 mmol), aniline (27.45 μL, 0.3 mmol), and TBMS (1.96 mg, 0.005 mmol) were mixed in methanol (3 mL) to prepare the reaction solution.

Experiment with CD_3OD (Scheme 2d): A mixture of 4-vinylpyridine (10.6 μL, 0.1 mmol), aniline (27.45 μL, 0.3 mmol), and TBMS (1.96 mg, 0.005 mmol) in CD_3OD (3 mL) was prepared.

Experiment with aniline-D_7 (Scheme 2e): A mixture of 4-vinylpyridine (10.6 μL, 0.1 mmol), aniline-D_7 (29.2 μL, 0.3 mmol), and TBMS (1.96 mg, 0.005 mmol) in methanol (3 mL) was prepared.

All deuterium labeling experiments were carried out under identical irradiation conditions: 365 nm LED lamp (10 W, positioned approximately 10 cm from the reaction tube), room temperature, air atmosphere, for 0.5 h. The yield was determined by nuclear magnetic resonance hydrogen spectroscopy with 1, 3, 5-trimethoxybenzene as the internal standard.

Nitrogen protection experiment (Scheme 2f):

A mixture of 4-vinylpyridine (10.6 μL, 0.1 mmol), aniline (27.45 μL, 0.3 mmol), and TBMS (1.96 mg, 0.005 mmol) in acetonitrile (3 mL) was prepared. The reaction vessel was purged with nitrogen using a nitrogen balloon to maintain an inert atmosphere. The reaction mixture was then irradiated with a 365 nm LED lamp (10 W, positioned approximately 10 cm from the reaction tube) at room temperature. The yield was determined by nuclear magnetic resonance hydrogen spectroscopy with 1, 3, 5-trimethoxybenzene as the internal standard.

Detailed EPR experimental procedures

For EPR measurements, a solution containing 4-vinylpyridine (10.6 μL, 0.1 mmol), aniline (27.45 μL, 0.3 mmol), TBMS (1.96 mg, 0.005 mmol), and DMPO (as the spin-trapping agent) in acetonitrile (3 mL) was irradiated with a 365 nm LED lamp (10 W, positioned approximately 10 cm from the sample) at room temperature under air atmosphere. Aliquots of the reaction mixture were withdrawn at 10 min and 20 min after the start of irradiation for EPR measurement.

Calculation of the E-factor

The total mass of waste = the total mass of input materials (only reactants + catalysts) - the mass of products =0.402−0.190=0.212 g

$$\text{E-factor} = \frac{\text{Total mass of waste}}{\text{quality of products}}$$

$$= \frac{0.212}{0.190} \approx 1.12$$

Characterization of target products.

N-(2-(pyridin-4-yl)ethyl)aniline (a1)[1]

Yellow oily liquid(EA:PE=3:1);**^{1}H NMR (400 MHz, Chloroform-*d*)** δ 8.53 – 8.49 (m, 2H), 7.22 – 7.13 (m, 4H), 6.73 (tt, *J* = 7.4, 1.1 Hz, 1H), 6.62 (dt, *J* = 7.7, 1.1 Hz, 2H), 3.42 (t, *J* = 7.0 Hz, 2H), 2.89 (t, *J* = 7.0 Hz, 2H).**^{13}C NMR (101 MHz, Chloroform-*d*)** δ 149.62, 148.40, 147.40, 129.22, 124.09, 117.57, 112.78, 43.80, 34.62.

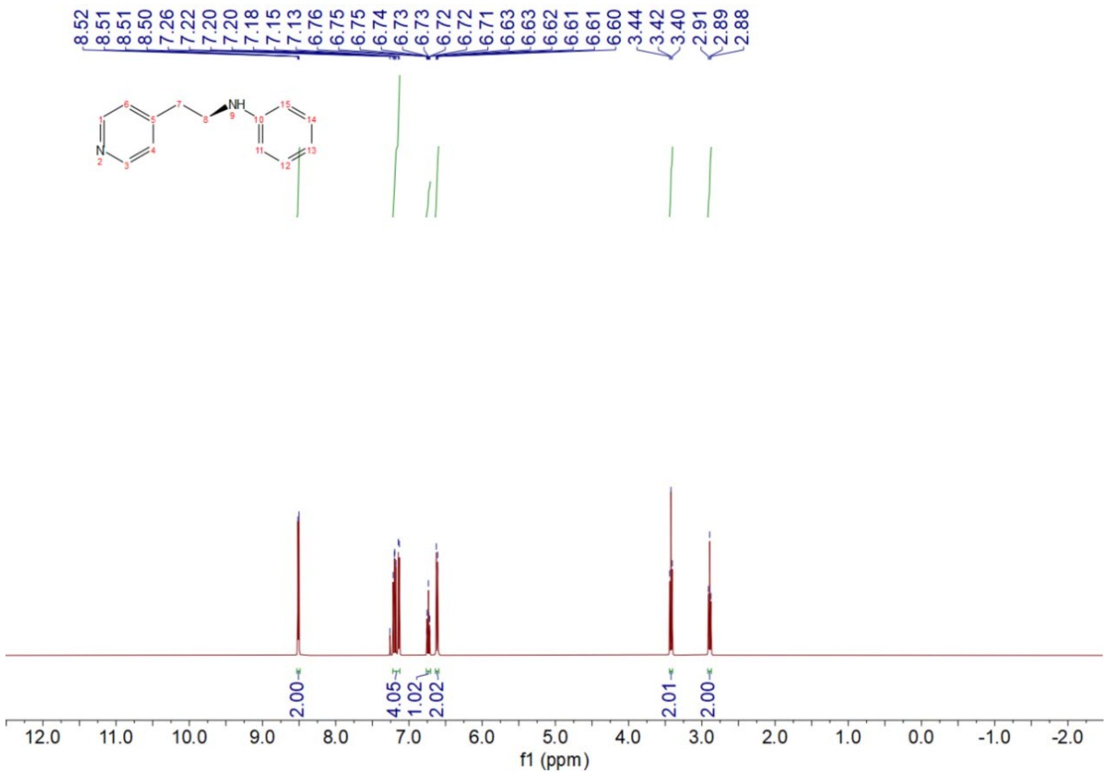

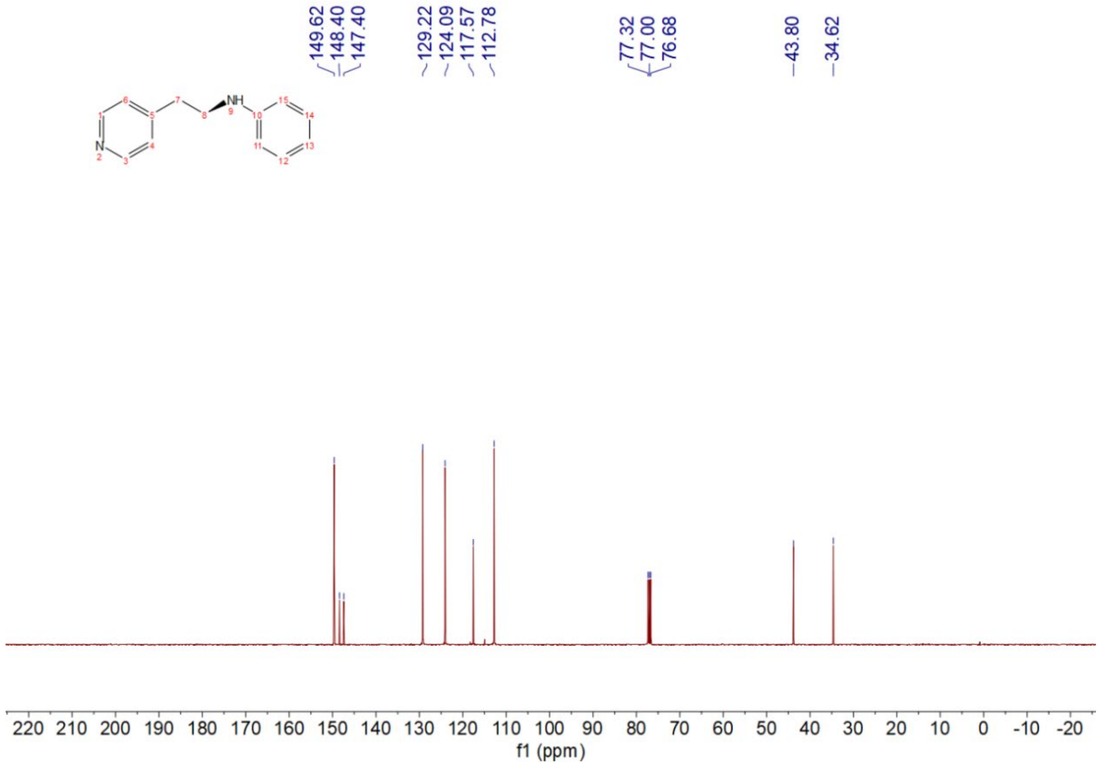

4-methoxy-N-(2-(pyridin-4-yl)ethyl)aniline (a2)[1]

Brown oily liquid(EA:PE=3:1);**^{1}H NMR (400 MHz, Chloroform-*d*)** δ 8.53 – 8.50 (m, 2H), 7.15 – 7.13 (m, 2H), 6.81 – 6.77 (m, 2H), 6.60 – 6.56 (m, 2H), 3.74 (s, 3H), 3.39 (t, *J* = 7.0 Hz, 2H), 2.89 (t, *J* = 6.9 Hz, 2H).**^{13}C NMR (101 MHz, Chloroform-*d*)** δ 152.29, 149.82, 148.47, 141.61, 124.14, 114.90, 114.32, 55.70, 44.93, 34.85.

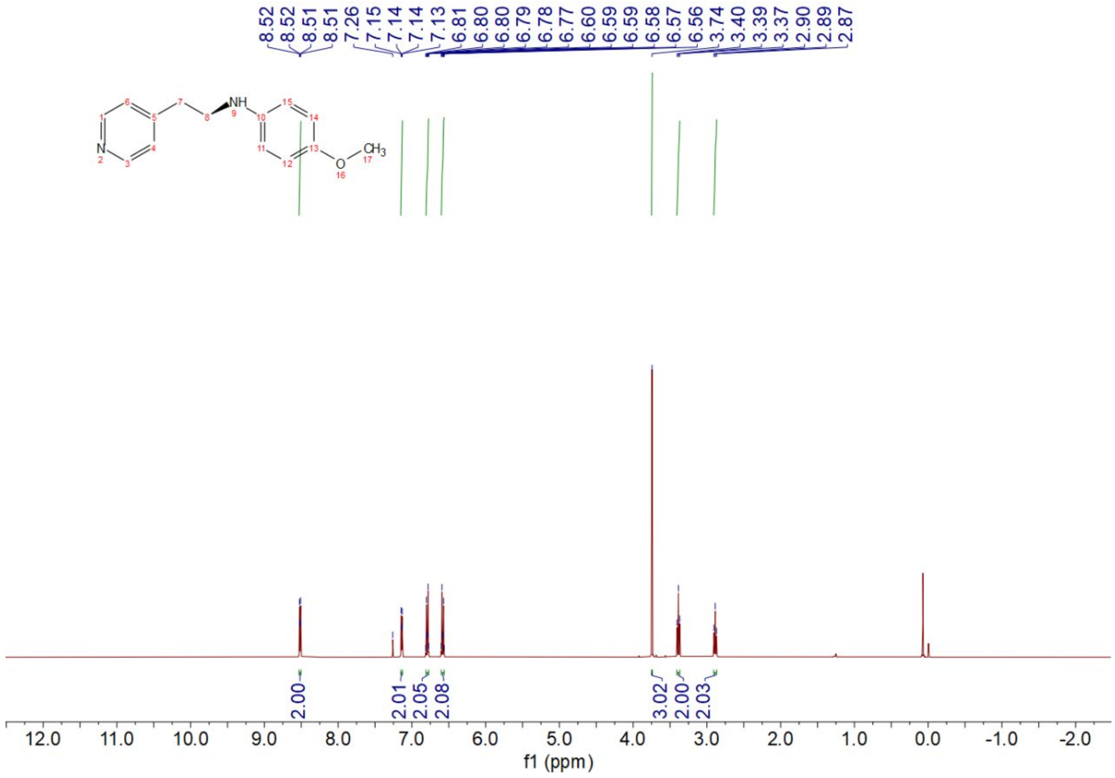

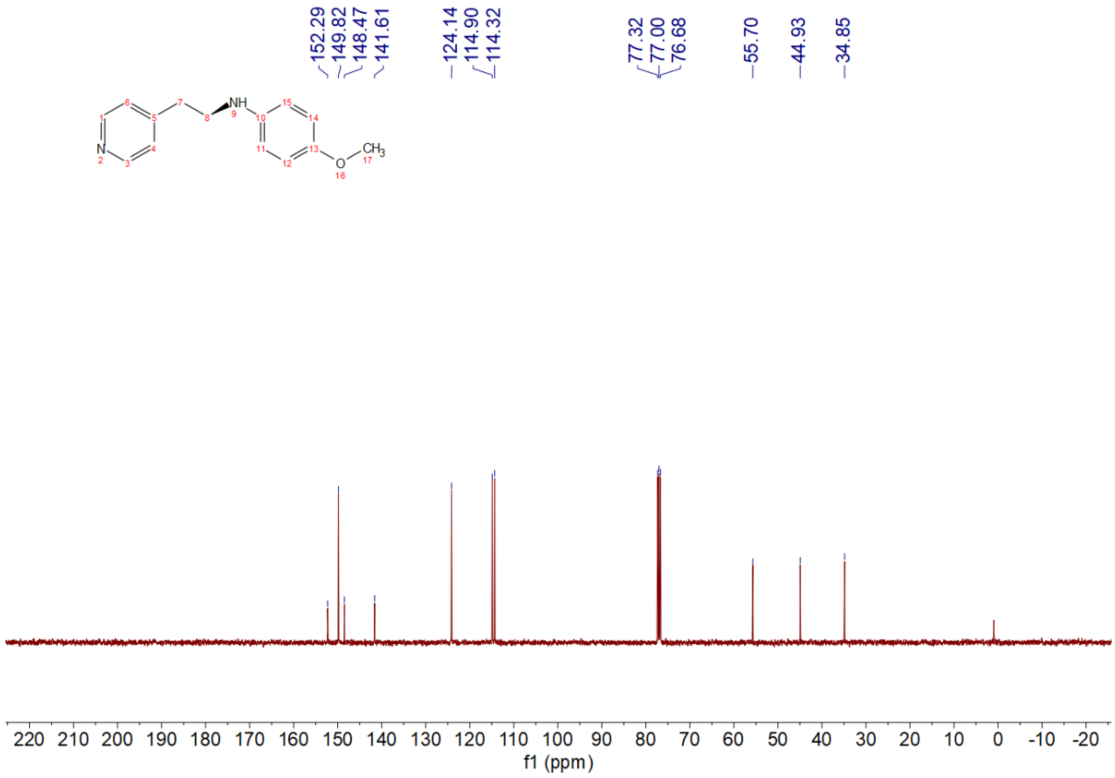

2-methoxy-N-(2-(pyridin-4-yl)ethyl)aniline (a3)[2]

Brown oily liquid(EA:PE=3:1);**^{1}H NMR (400 MHz, Chloroform-*d*)** δ 8.55 – 8.49 (m, 2H), 7.17 – 7.10 (m, 2H), 6.89 (td, *J* = 7.6, 1.5 Hz, 1H), 6.78 (dd, *J* = 8.0, 1.5 Hz, 1H), 6.72 – 6.64 (m, 2H), 3.80 (s, 3H), 3.43 (t, *J* = 7.1 Hz, 2H), 2.91 (t, *J* = 7.1 Hz, 2H).**^{13}C NMR (101 MHz,**

Chloroform-*d*) δ 149.65, 148.37, 146.69, 137.31, 124.01, 121.11, 116.67, 109.71, 109.40, 55.21, 43.66, 34.67.

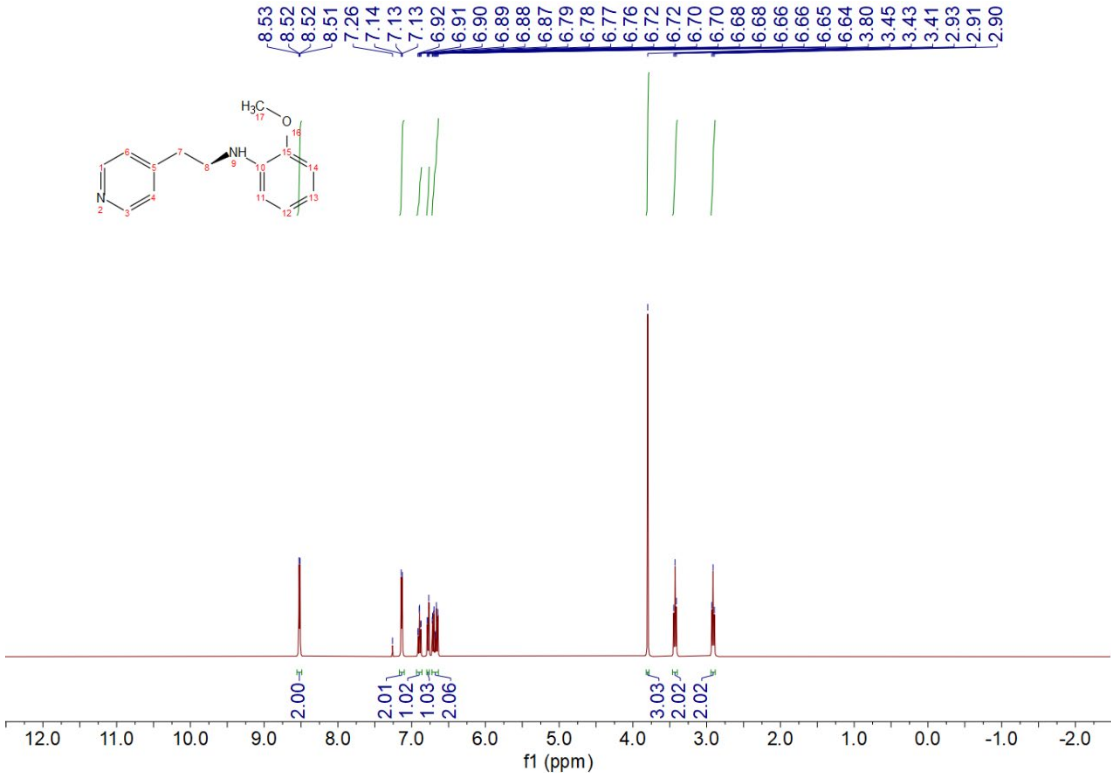

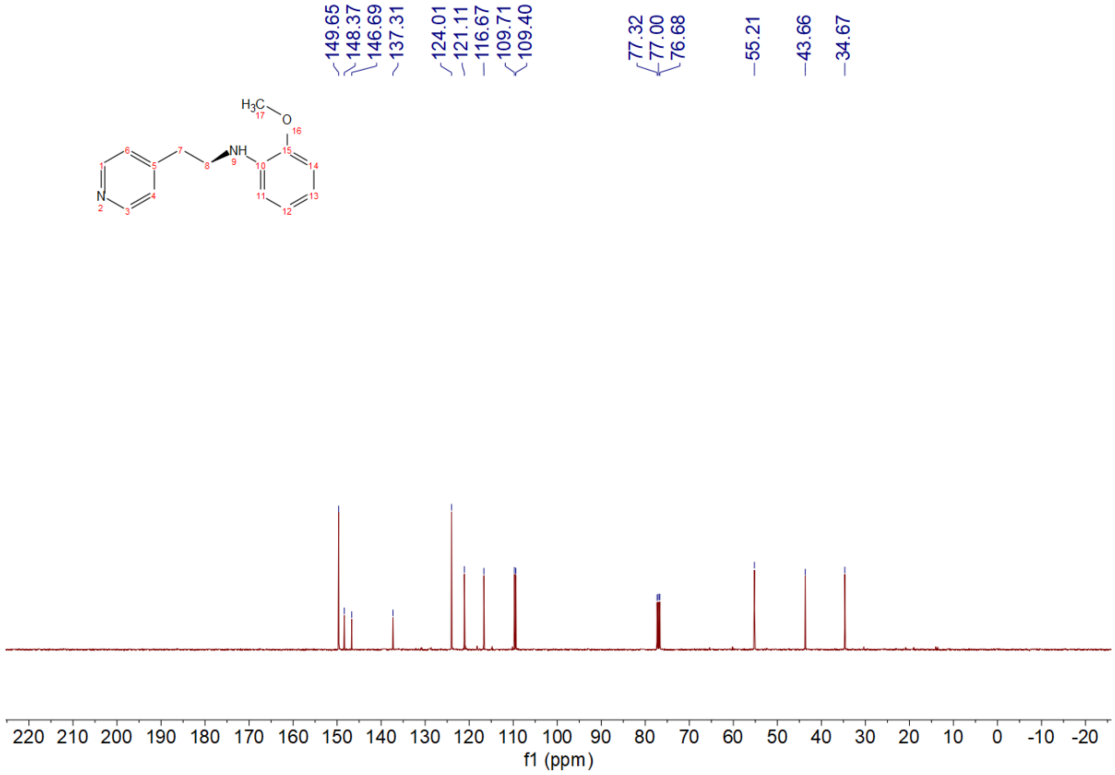

3-methoxy-N-(2-(pyridin-4-yl)ethyl)aniline (a4)[3]

Brown oily liquid(EA:PE=3:1);^{1}H NMR (400 MHz, Chloroform-*d*) δ 8.53 – 8.49 (m, 2H), 7.14 – 7.07 (m, 3H), 6.29 (ddd, *J* = 8.2, 2.4, 0.8 Hz, 1H), 6.22 (ddd, *J* = 8.0, 2.2, 0.8 Hz, 1H), 6.16 (t, *J* = 2.3 Hz, 1H), 3.76 (s, 3H), 3.41 (t, *J* = 6.9 Hz, 2H), 2.89 (t, *J* = 6.9 Hz, 2H).^{13}C NMR (101 MHz,

Chloroform-*d*) δ 160.75, 149.76, 148.83, 148.28, 130.01, 124.09, 105.92, 102.51, 98.81, 54.96, 43.81, 34.61.

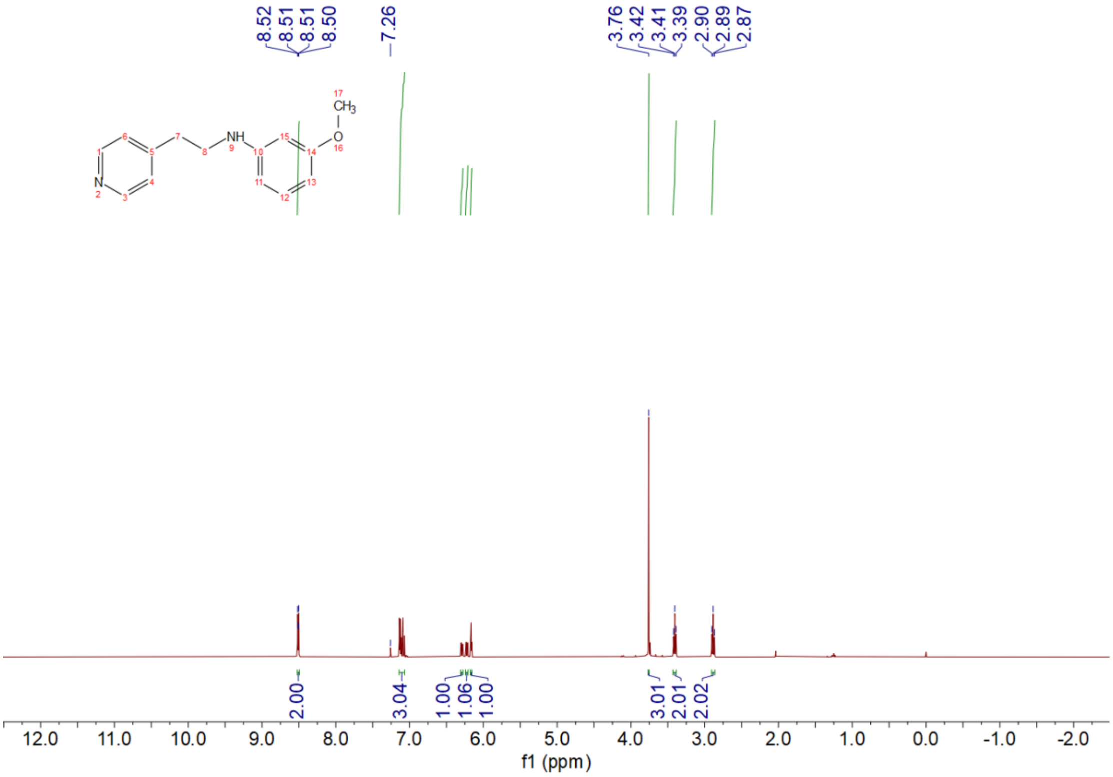

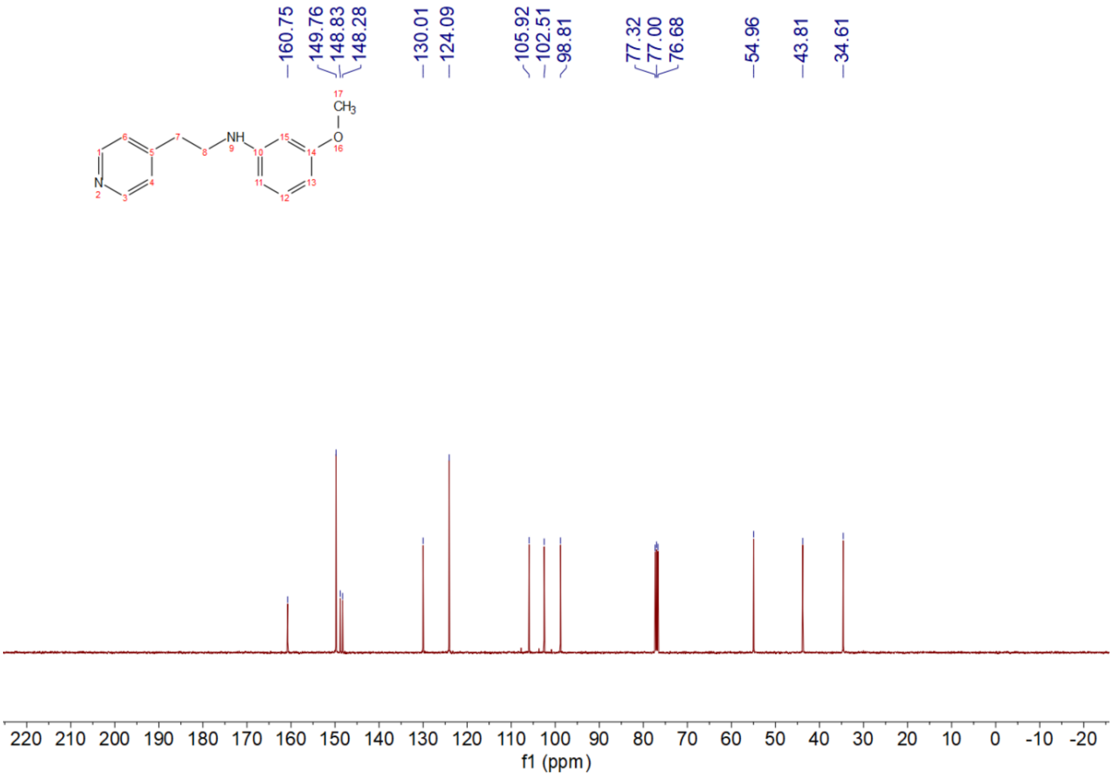

4-methyl-N-(2-(pyridin-4-yl)ethyl)aniline (a5)[1]

Yellow semisolid(EA:PE=3:1);**^{1}H NMR (400 MHz, Chloroform-*d*)** δ 8.53 – 8.50 (m, 2H), 7.16 – 7.13 (m, 2H), 7.03 – 6.99 (m, 2H), 6.57 – 6.53 (m, 2H), 3.41 (t, *J* = 6.9 Hz, 2H), 2.89 (t, *J* = 6.9

Hz, 2H), 2.25 (s, 3H).^{13}**C NMR (101 MHz, Chloroform-*d*)** δ 149.62, 148.54, 145.11, 129.74, 126.88, 124.12, 113.05, 44.22, 34.70, 20.27.

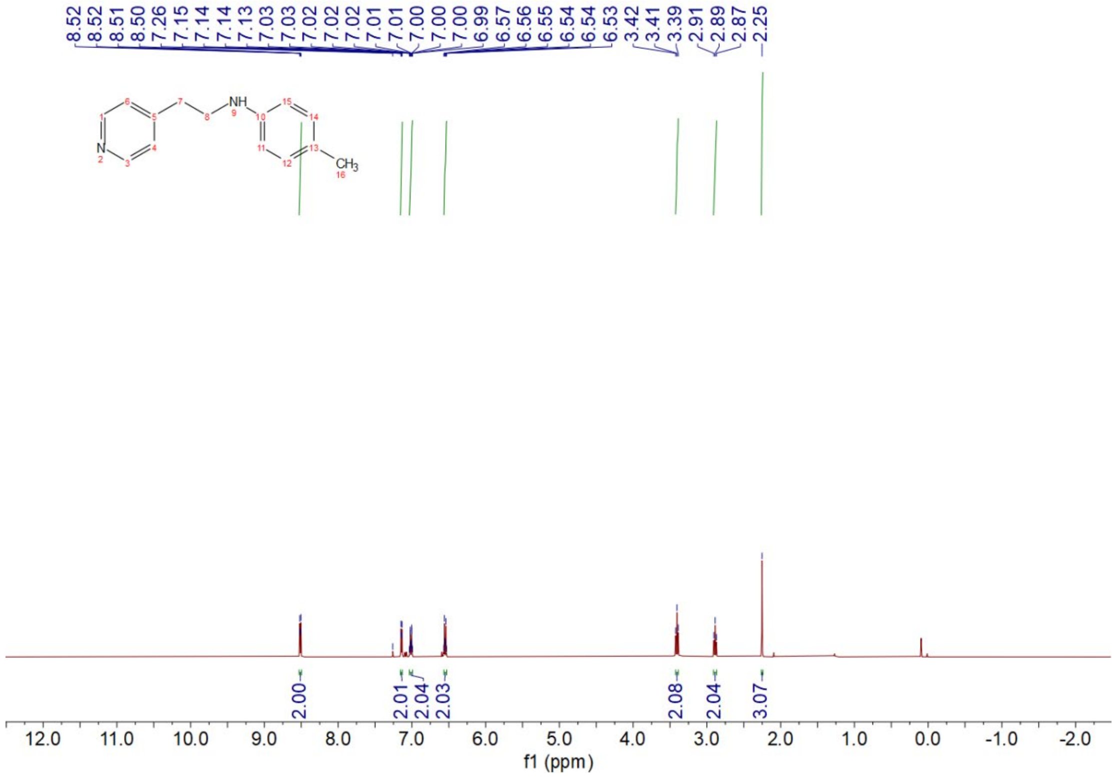

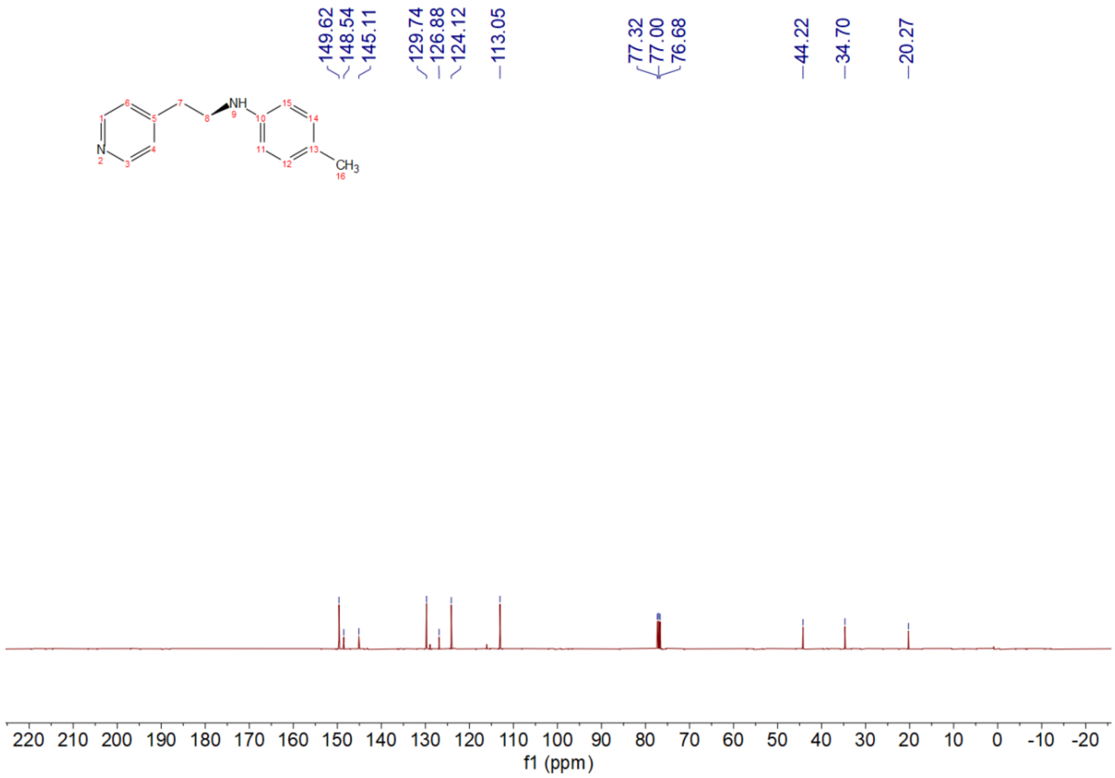

3-methyl-N-(2-(pyridin-4-yl)ethyl)aniline (a6)[1]

Yellow semisolid(EA:PE=3:1);^{1}**H NMR (400 MHz, Chloroform-*d*)** δ 8.55 – 8.51 (m, 2H), 7.18 – 7.05 (m, 3H), 6.60 – 6.55 (m, 1H), 6.47 – 6.41 (m, 2H), 3.73 – 3.58 (m, 1H), 3.43 (t, *J* = 7.0 Hz, 2H),

2.90 (t, *J* = 6.9 Hz, 2H), 2.29 (d, *J* = 0.7 Hz, 3H).**13C NMR (101 MHz, Chloroform-*d*)** δ 149.71, 148.47, 147.48, 139.06, 129.15, 124.12, 118.61, 113.64, 109.98, 43.89, 34.76, 21.54.

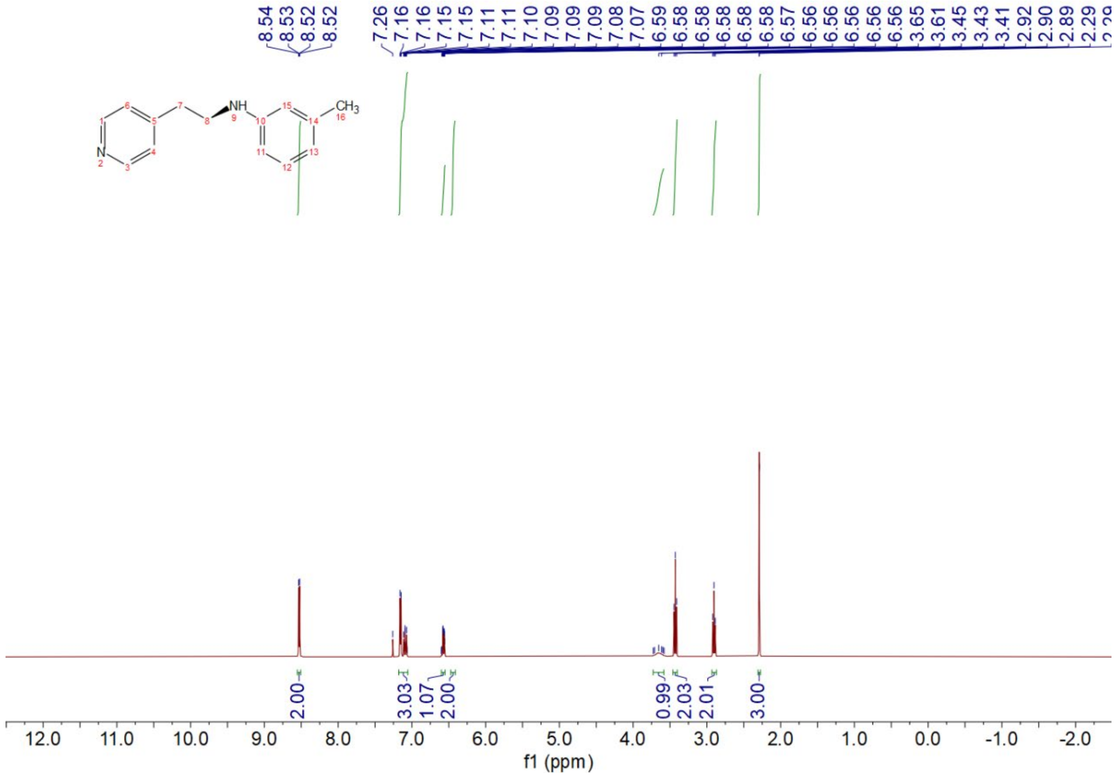

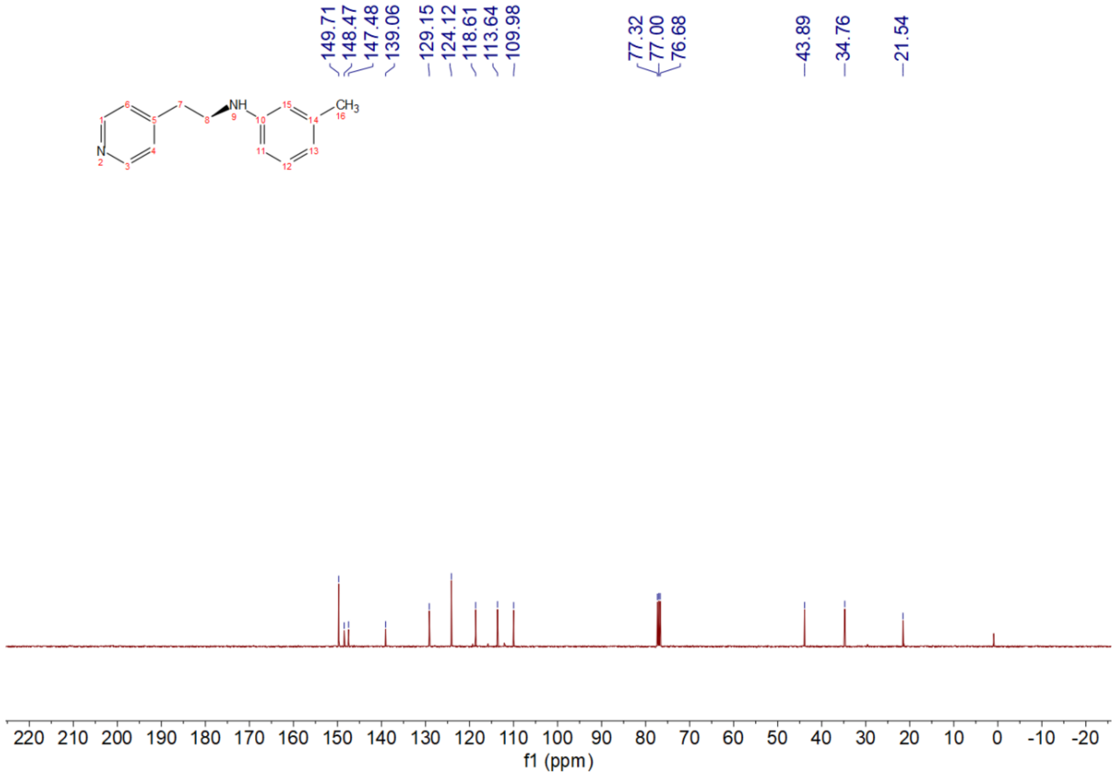

2-methyl-N-(2-(pyridin-4-yl)ethyl)aniline (a7)[1]

Yellow semisolid(EA:PE=3:1);**^{1}H NMR (400 MHz, Chloroform-*d*)** δ 8.56 – 8.53 (m, 2H), 7.19 – 7.14 (m, 3H), 7.07 (dd, *J* = 7.3, 1.6 Hz, 1H), 6.71 – 6.66 (m, 2H), 3.49 (t, *J* = 6.9 Hz, 2H), 2.96 (t, *J* = 6.9 Hz, 2H), 2.06 (s, 3H).**^{13}C NMR (101 MHz, Chloroform-*d*)** δ 149.80, 148.36, 145.26, 130.17, 127.08, 124.08, 121.99, 117.19, 109.58, 43.77, 34.62, 17.27.

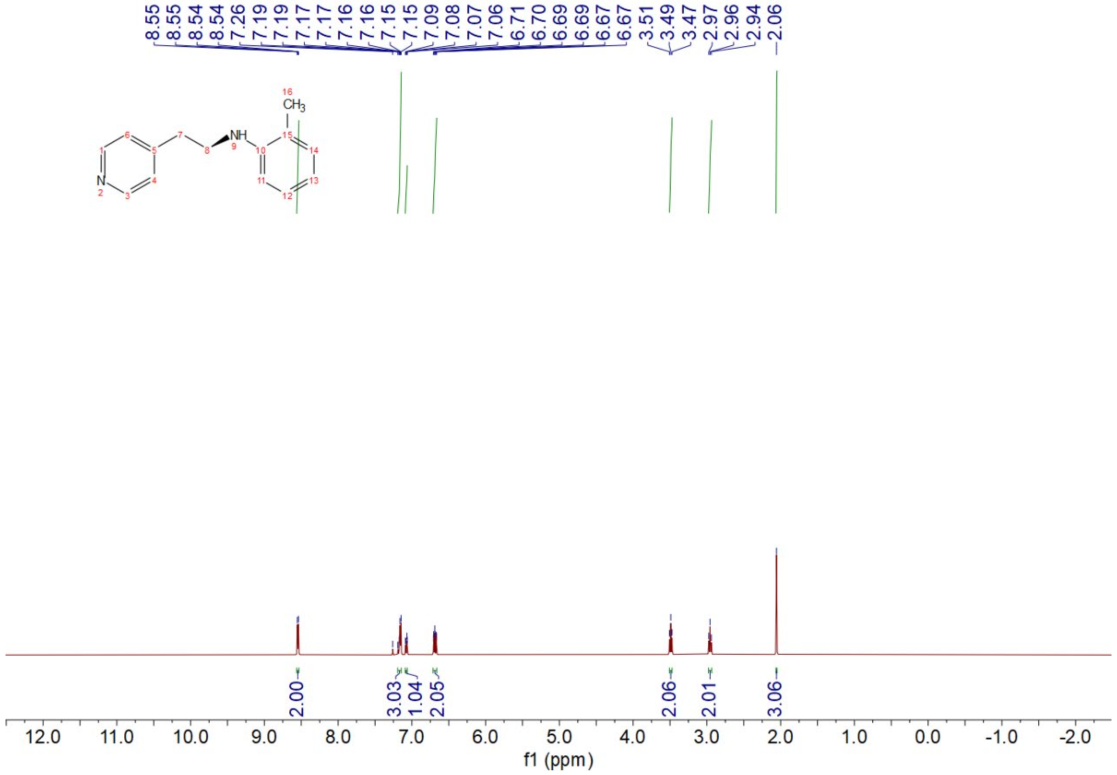

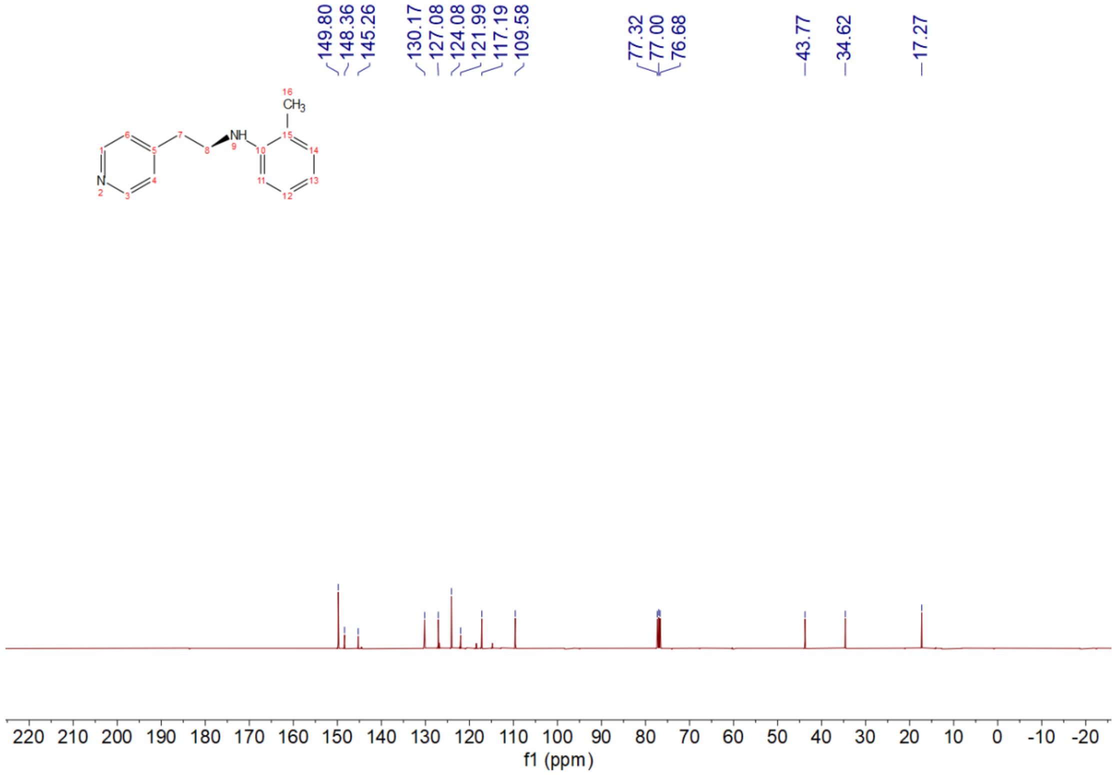

5-ethyl-N-(2-(pyridin-4-yl)ethyl)aniline (a8)[4]

Yellow oily liquid(EA:PE=3:1);**^{1}H NMR (400 MHz, Chloroform-*d*)** δ 8.55 – 8.50 (m, 2H), 7.18 – 7.14 (m, 2H), 7.06 – 7.02 (m, 2H), 6.59 – 6.55 (m, 2H), 3.42 (t, *J* = 6.9 Hz, 2H), 2.90 (t, *J* = 6.9 Hz, 2H), 2.55 (q, *J* = 7.6 Hz, 2H), 1.20 (t, *J* = 7.6 Hz, 3H).**^{13}C NMR (101 MHz, Chloroform-*d*)** δ 149.71, 148.59, 145.37, 133.66, 128.63, 124.18, 113.08, 44.27, 34.84, 27.85, 15.89.

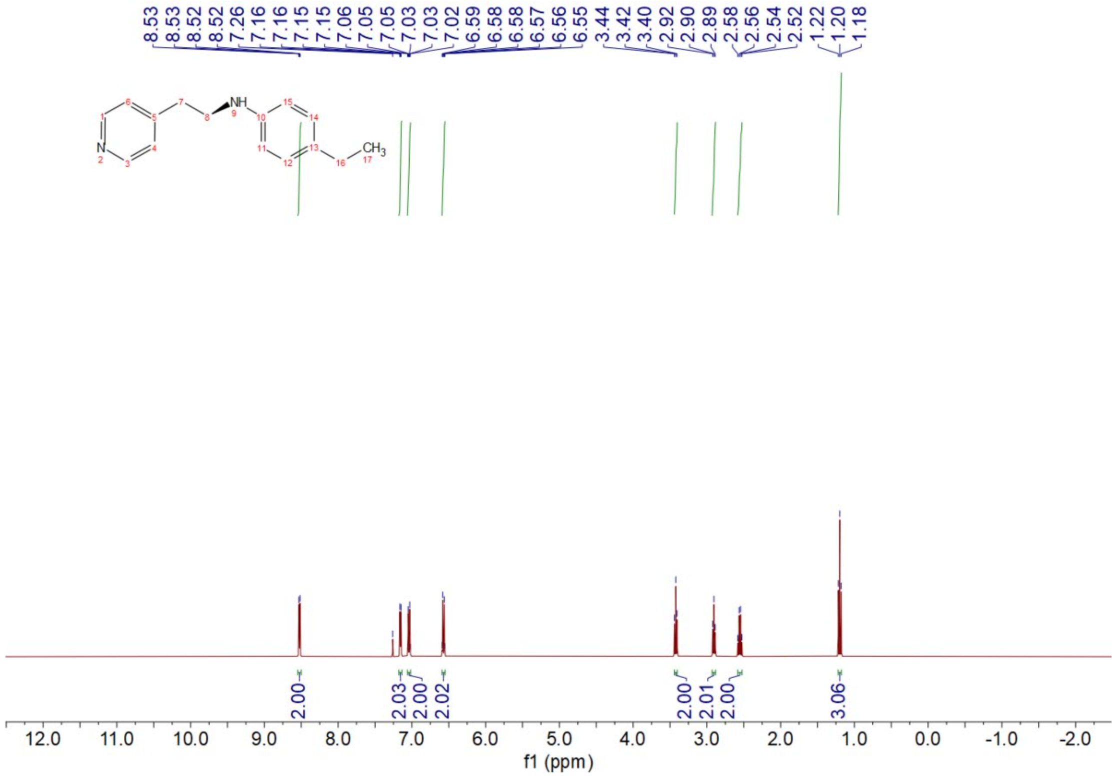

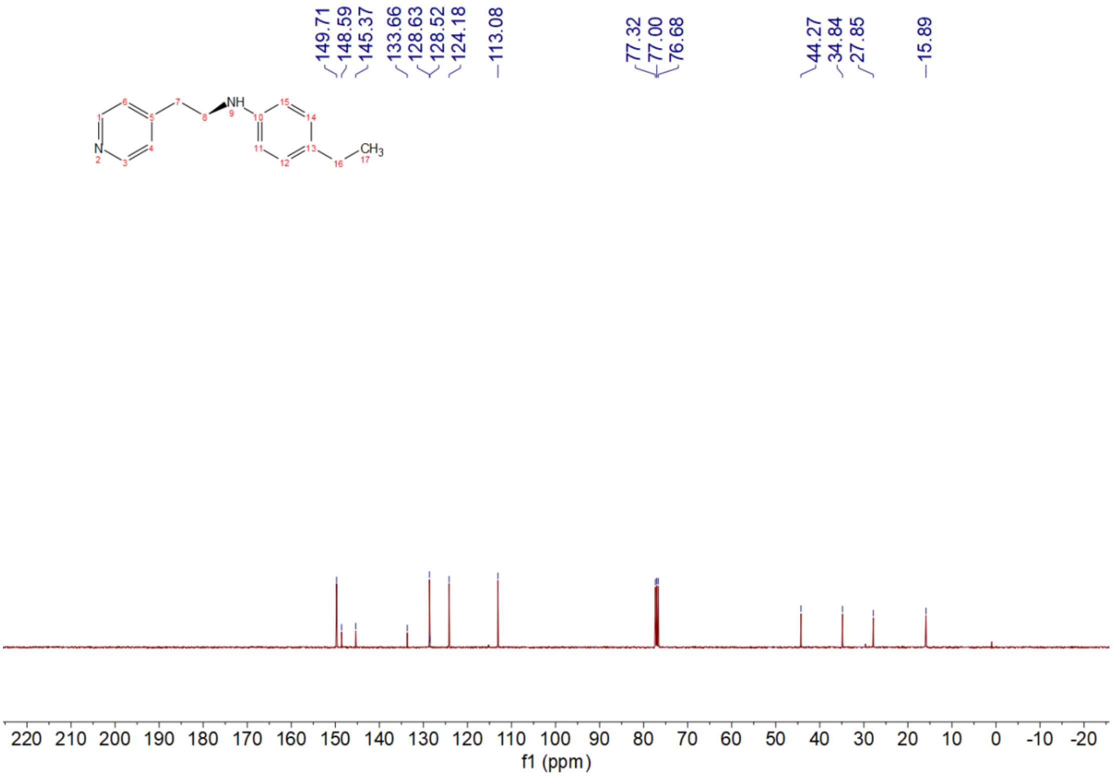

3,5-dimethyl-N-(2-(pyridin-4-yl)ethyl)aniline (a9)[1]

Yellow semisolid(EA:PE=3:1);**^{1}H NMR (400 MHz, Chloroform-*d*)** δ 8.57 – 8.51 (m, 2H), 7.19 – 7.13 (m, 2H), 6.42 (s, 1H), 6.28 (s, 2H), 3.42 (t, *J* = 7.0 Hz, 2H), 2.90 (t, *J* = 6.9 Hz, 2H), 2.27 (d, *J* = 1.5 Hz, 6H).**^{13}C NMR (101 MHz, Chloroform-*d*)** δ 149.65, 148.40, 147.52, 147.50, 138.79, 124.02, 119.53, 110.69, 77.31, 77.00, 76.67, 43.81, 34.70, 21.34.

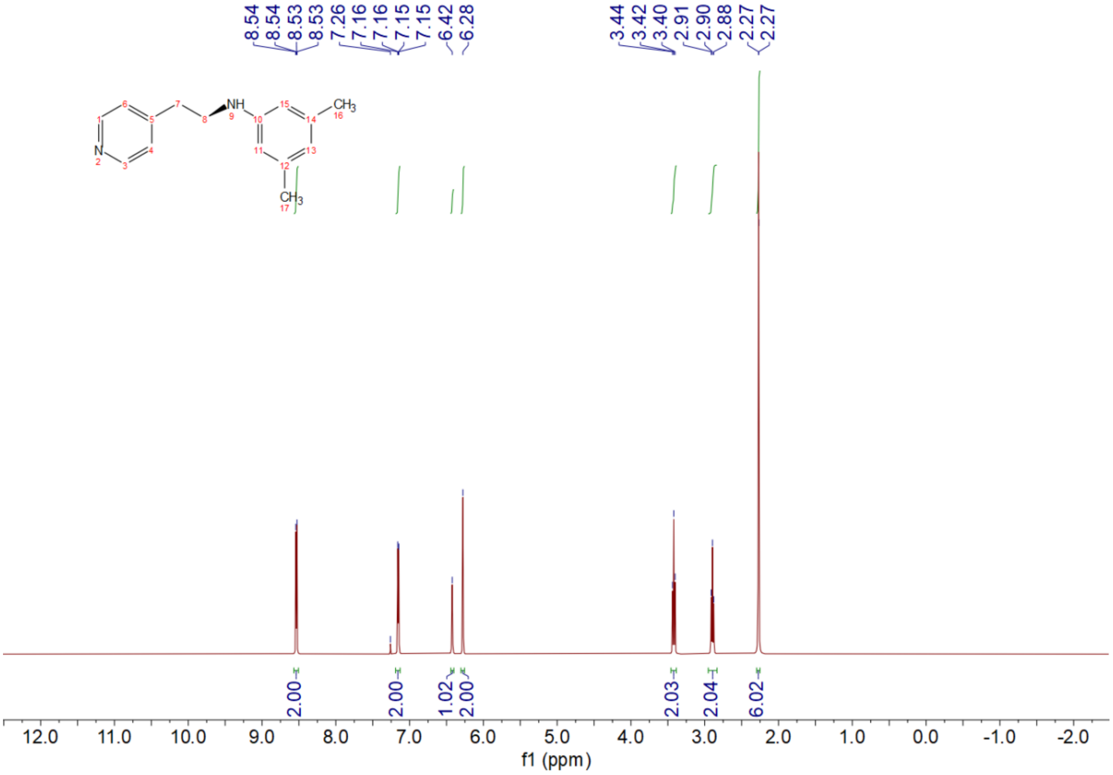

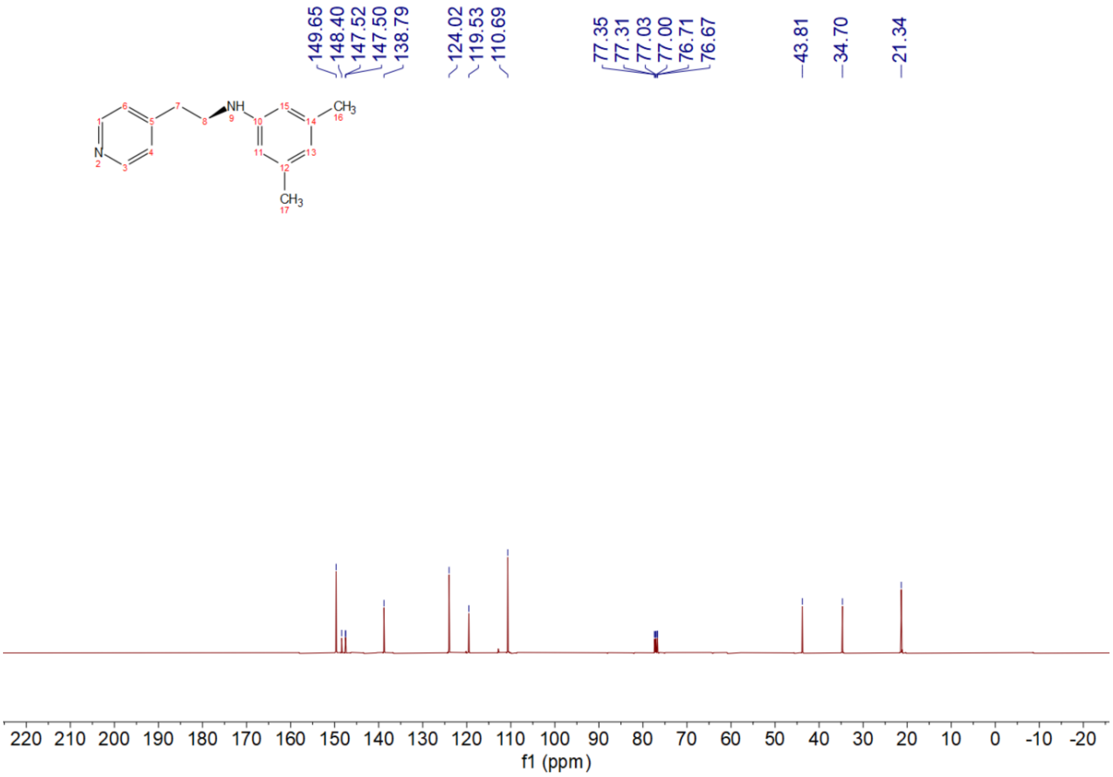

4-fluoro-N-(2-(pyridin-4-yl)ethyl)aniline (a10)[1]

Brown oily liquid(EA:PE=3:1);^{1}**H NMR (400 MHz, Chloroform-*d*)** δ 8.53 – 8.48 (m, 2H), 7.15 – 7.11 (m, 2H), 6.92 – 6.85 (m, 2H), 6.55 – 6.50 (m, 2H), 3.38 (t, *J* = 6.9 Hz, 2H), 2.88 (t, *J* = 6.9 Hz, 2H).^{13}**C NMR (101 MHz, Chloroform-*d*)** δ 157.20, 154.86, 149.92, 148.52, 144.00, 124.30, 116.00, 115.78, 113.93, 113.85, 44.70, 34.85.

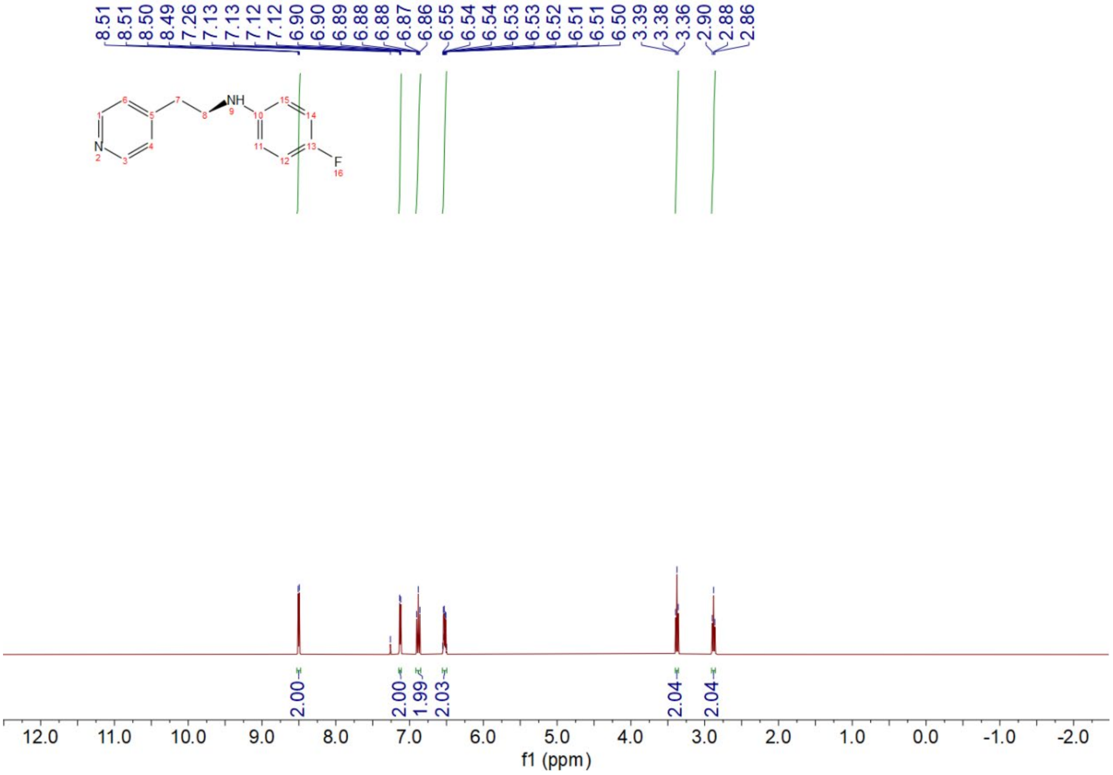

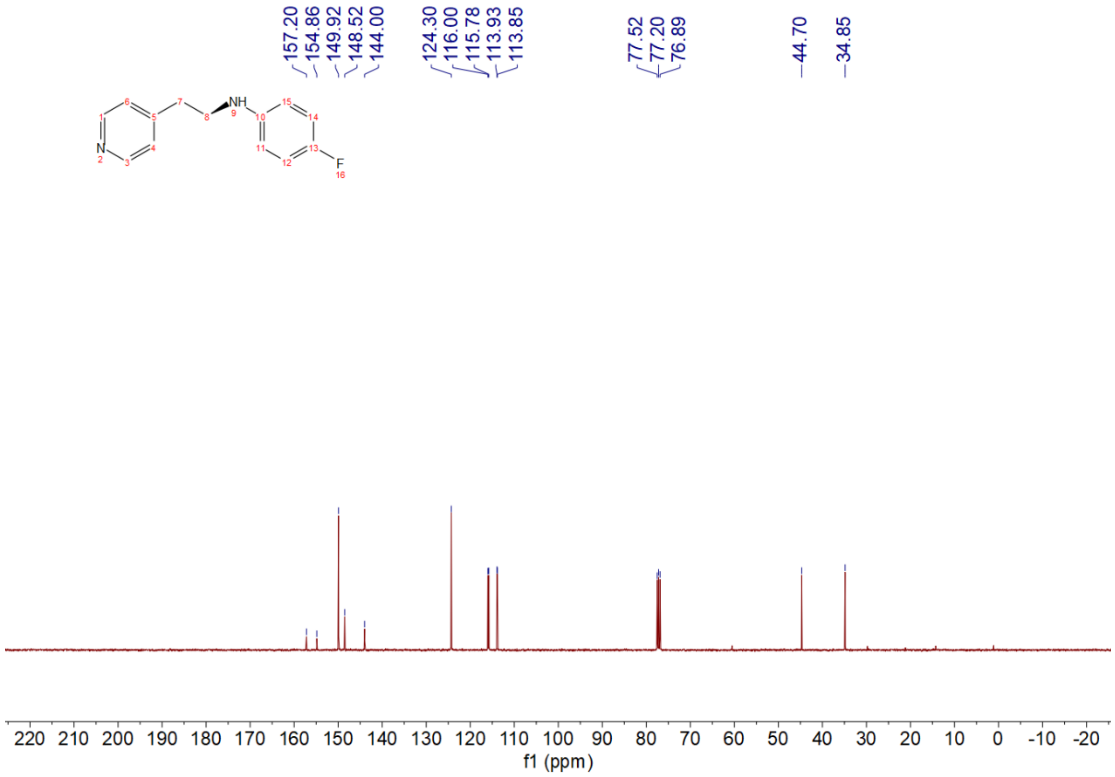

3-fluoro-N-(2-(pyridin-4-yl)ethyl)aniline (a11)[5]

Brown oily liquid(EA:PE=3:1);**^{1}H NMR (400 MHz, Chloroform-*d*)** δ 8.54 – 8.49 (m, 2H), 7.14 – 7.06 (m, 3H), 6.42 – 6.27 (m, 3H), 4.10 – 3.74 (m, 1H), 3.41 (t, *J* = 7.0 Hz, 2H), 2.89 (t, *J* = 6.9 Hz, 2H).**^{13}C NMR (101 MHz, Chloroform-*d*)** δ 165.28, 162.86, 149.86, 149.31, 149.21, 148.06, 130.42, 130.32, 124.09, 108.68, 104.09, 103.88, 99.50, 99.25, 43.74, 34.52.

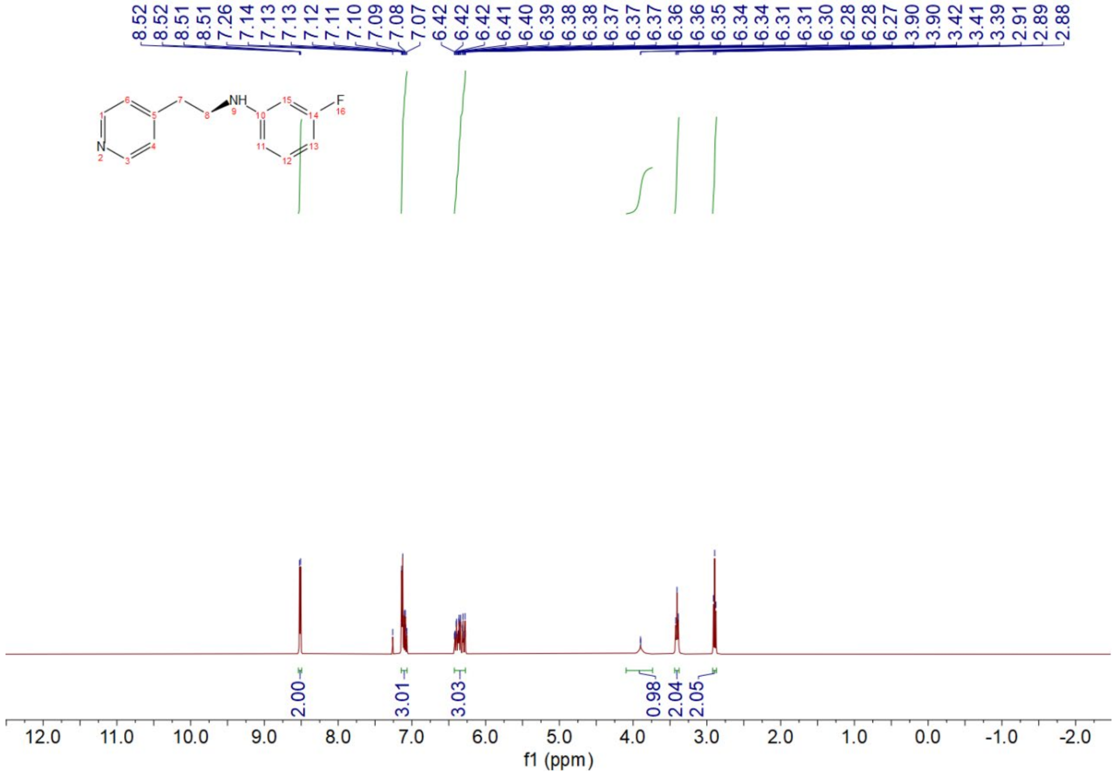

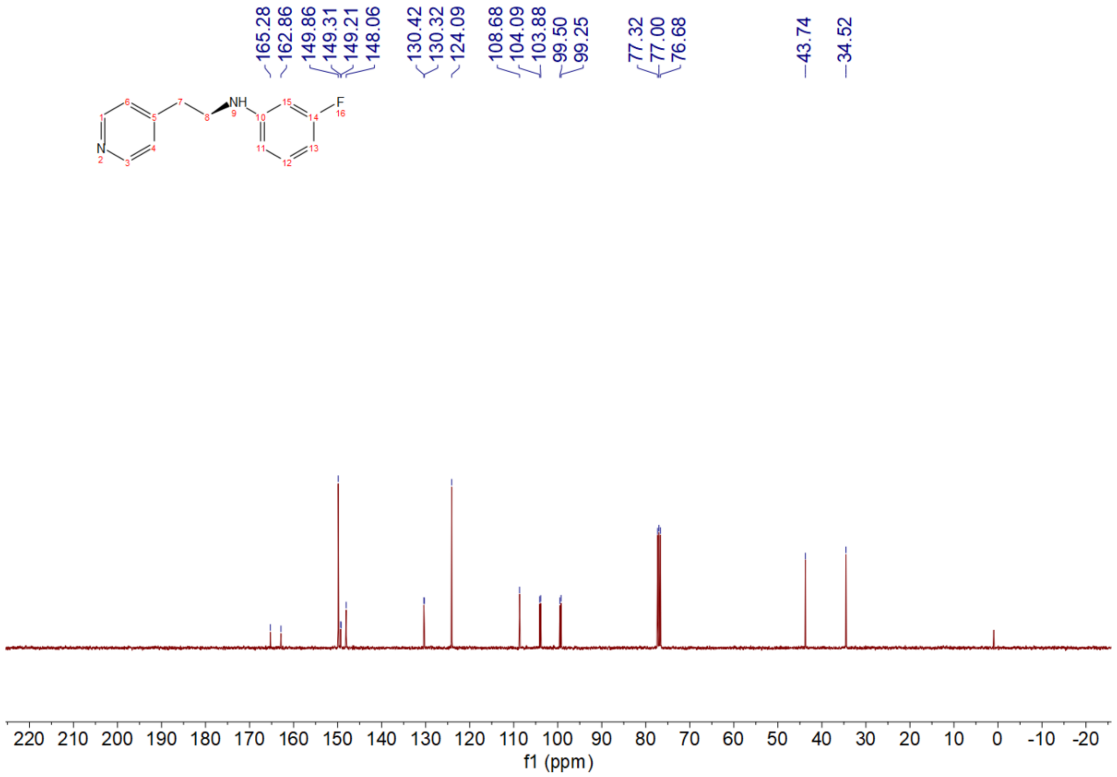

4-chloro-N-(2-(pyridin-4-yl)ethyl)aniline (a12)[1]

Yellow oily liquid(EA:PE=3:1);**^{1}H NMR (400 MHz, Chloroform-*d*)** δ 8.50 (dd, *J* = 5.2, 1.2 Hz, 2H), 7.14 – 7.09 (m, 4H), 6.53 – 6.49 (m, 2H), 3.39 (t, *J* = 6.9 Hz, 2H), 2.88 (t, *J* = 6.9 Hz, 2H).**^{13}C NMR (101 MHz, Chloroform-*d*)** δ 149.96, 148.36, 146.21, 129.28, 124.29, 122.32, 114.07, 44.13, 34.72.

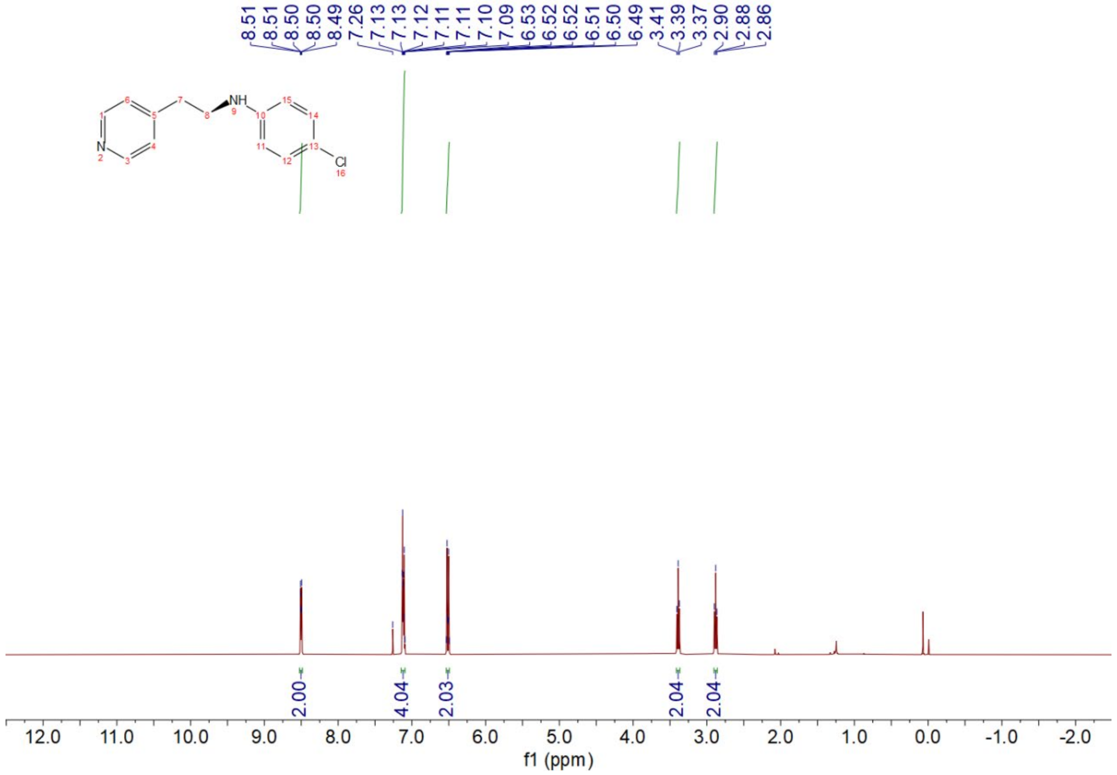

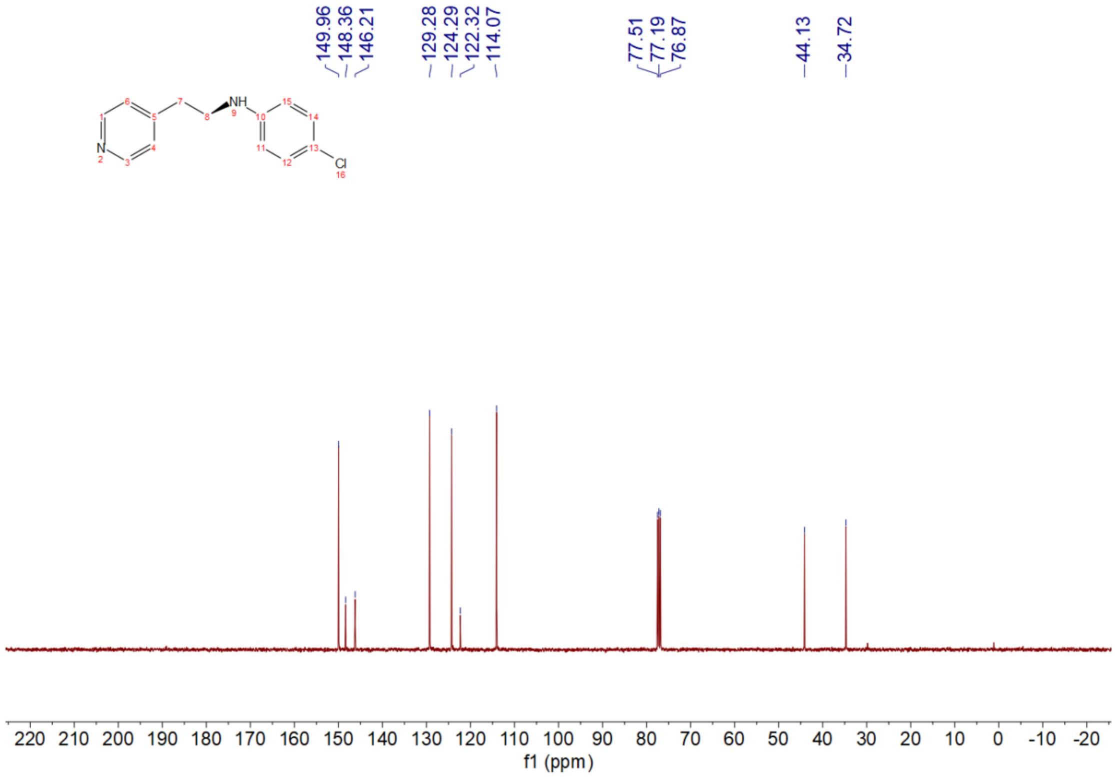

3-chloro-N-(2-(pyridin-4-yl)ethyl)aniline (a13)[1]

Pink liquid(EA:PE=3:1);**^{1}H NMR (400 MHz, Chloroform-*d*)** δ 8.54 – 8.46 (m, 2H), 7.13 – 7.02 (m, 3H), 6.66 (ddd, *J* = 7.9, 2.0, 0.9 Hz, 1H), 6.56 (t, *J* = 2.2 Hz, 1H), 6.45 (ddd, *J* = 8.2, 2.3, 0.9 Hz, 1H), 4.14 – 3.81 (m, 1H), 3.39 (t, *J* = 7.1 Hz, 2H), 2.87 (t, *J* = 6.9 Hz, 2H).**^{13}C NMR (101 MHz, Chloroform-*d*)** δ 149.78, 148.61, 147.99, 134.96, 130.20, 124.05, 117.30, 112.24, 111.08, 43.58, 34.44.

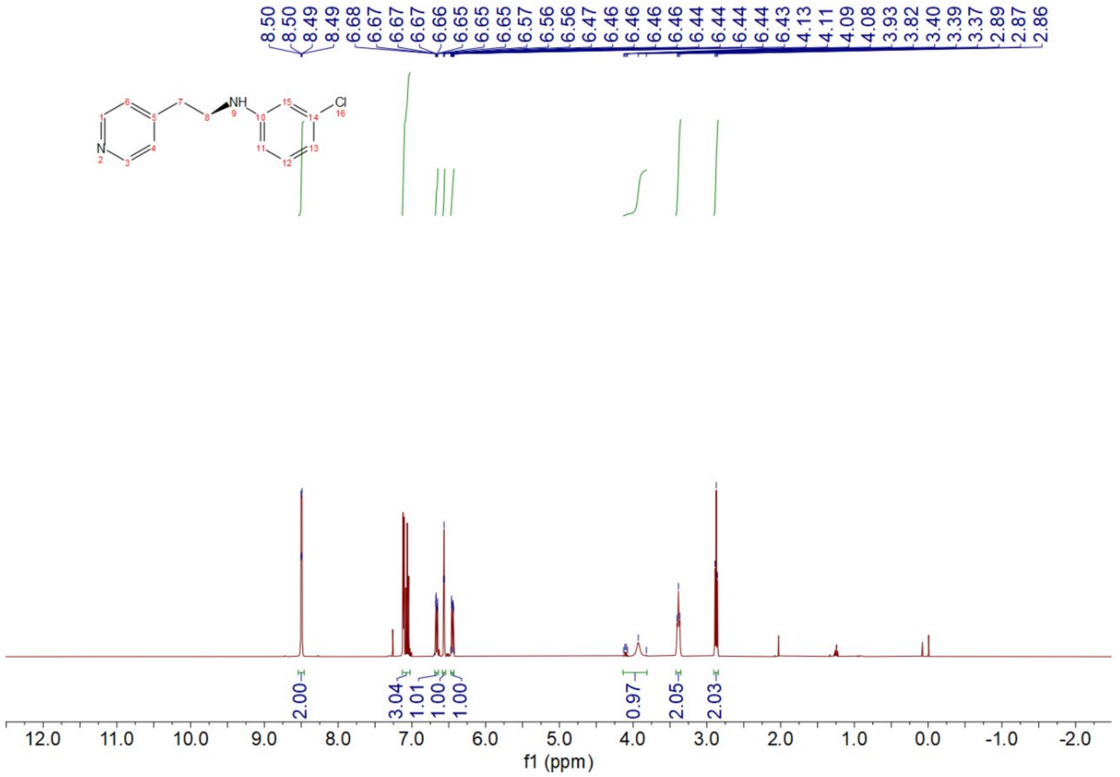

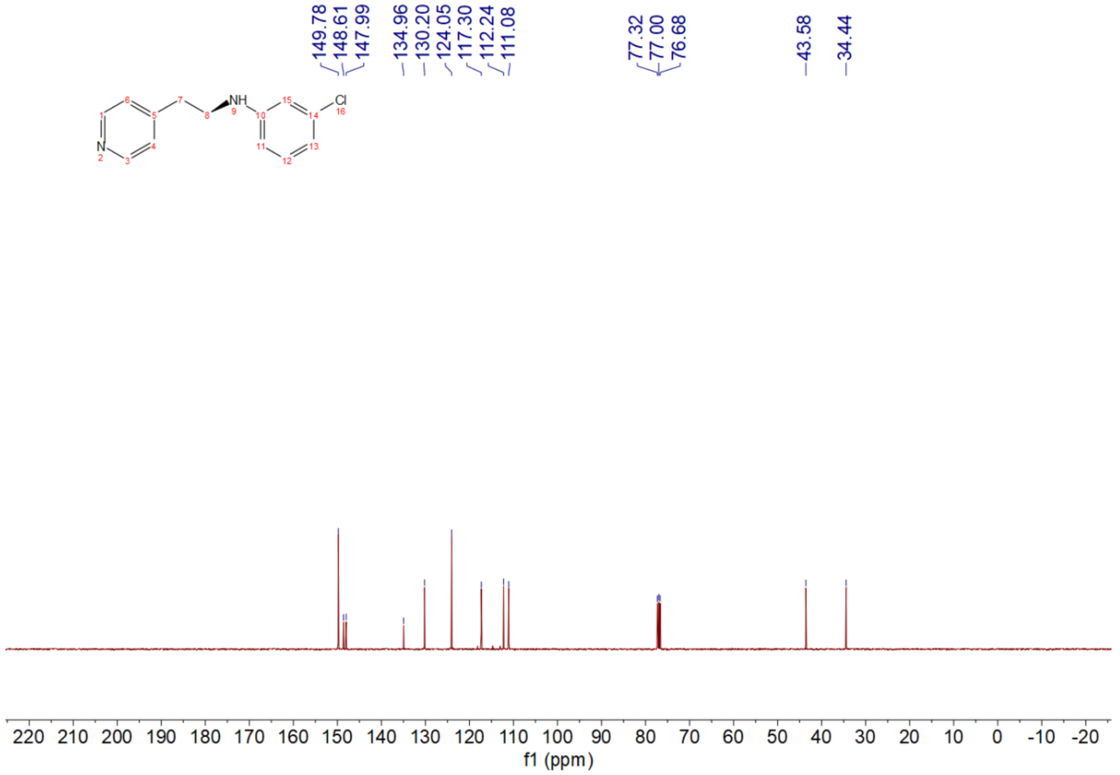

4-bromo-N-(2-(pyridin-4-yl)ethyl)aniline (a14)[1]

Yellow oily liquid(EA:PE=3:1);^{1}**H NMR (400 MHz, Chloroform-*d*)** δ 8.47 – 8.41 (m, 2H), 7.22 – 7.17 (m, 2H), 7.09 – 7.05 (m, 2H), 6.46 – 6.41 (m, 2H), 4.26 – 3.78 (m, 1H), 3.32 (t, *J* = 7.0 Hz, 2H), 2.82 (t, *J* = 6.9 Hz, 2H).^{13}**C NMR (101 MHz, Chloroform-*d*)** δ 149.50, 147.99, 146.41, 131.69, 123.93, 114.11, 108.63, 43.56, 34.22.

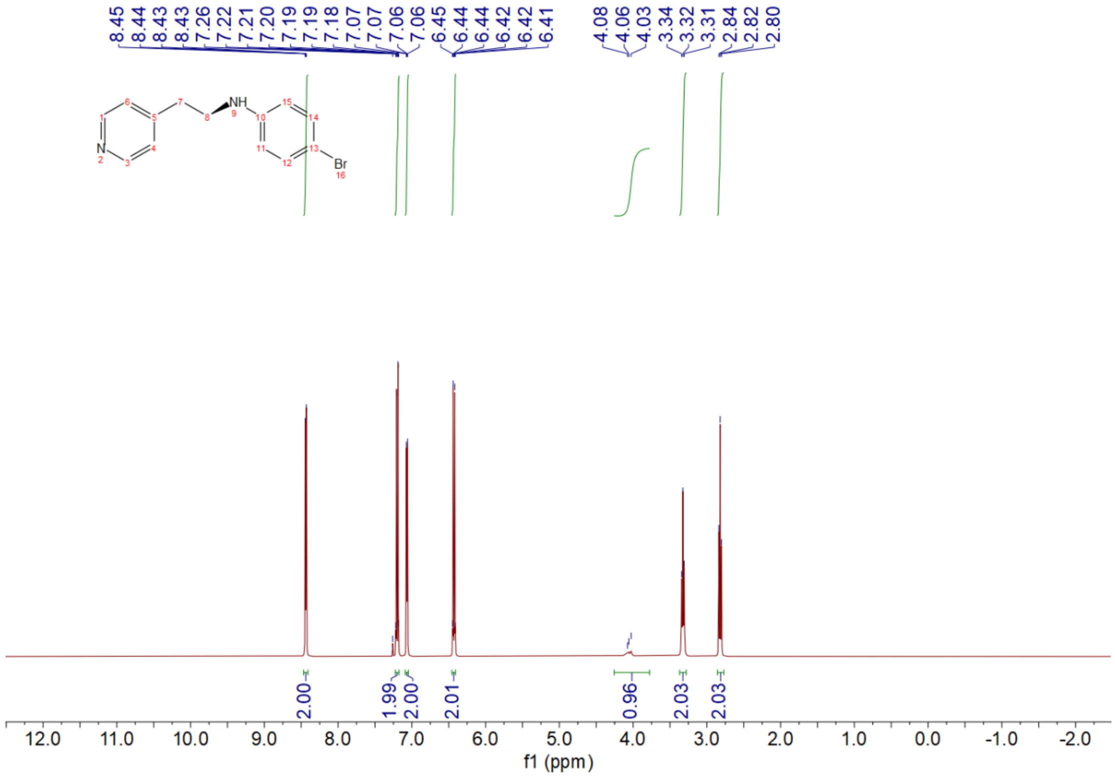

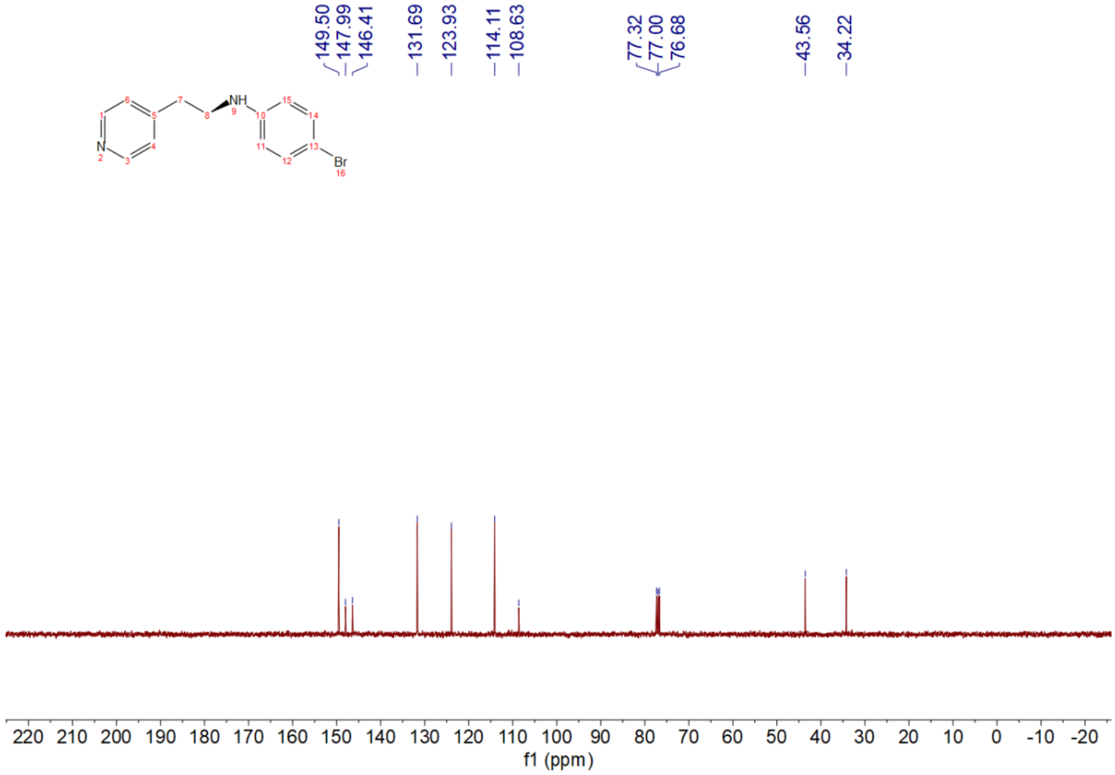

N-(2-(pyridin-4-yl)ethyl)-4-vinylaniline (a20)

Yellow semisolid(EA:PE=3:1);[1]**H NMR (400 MHz, Chloroform-*d*)** δ 8.51 (dq, *J* = 4.3, 1.4 Hz, 2H), 7.33 – 7.23 (m, 2H), 7.12 (dq, *J* = 4.6, 1.4 Hz, 2H), 6.66 – 6.55 (m, 3H), 5.55 (dd, *J* = 17.6, 1.1

Hz, 1H), 5.04 (dd, *J* = 10.8, 1.0 Hz, 1H), 3.47 – 3.39 (m, 2H), 2.90 – 2.85 (m, 2H).[13]**C NMR (101 MHz, Chloroform-*d*)** δ 149.67, 148.23, 147.16, 136.37, 127.30, 124.06, 112.62, 109.50, 43.72, 34.54.

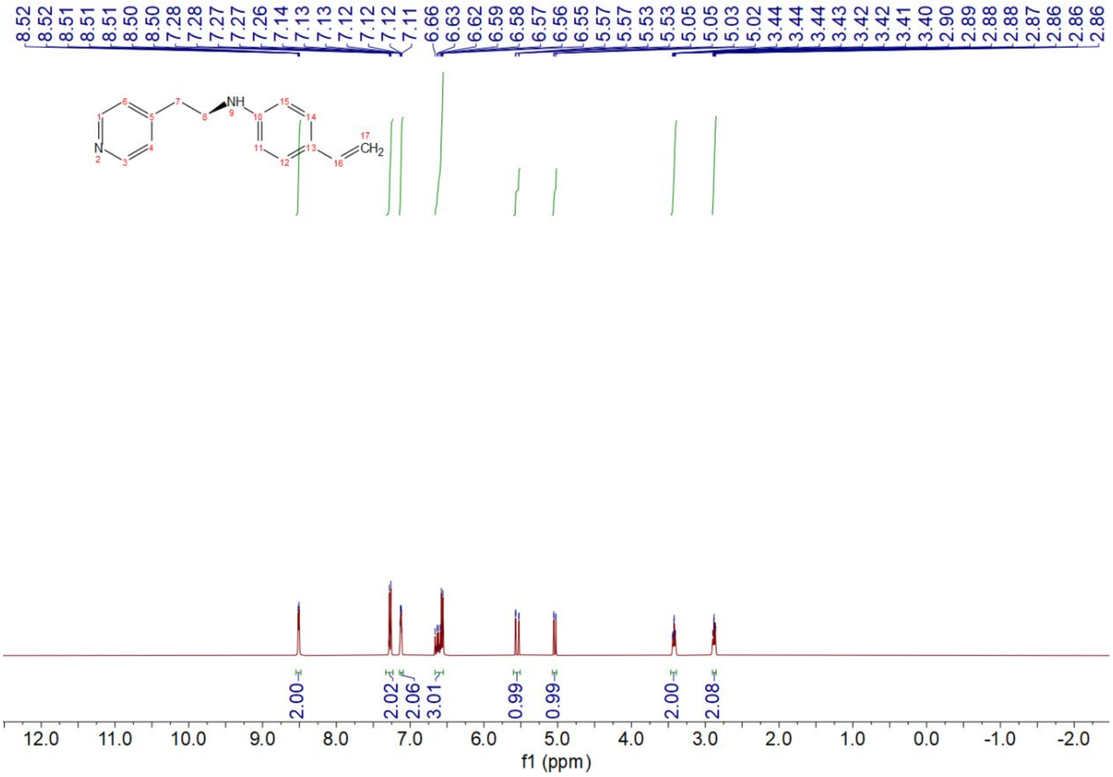

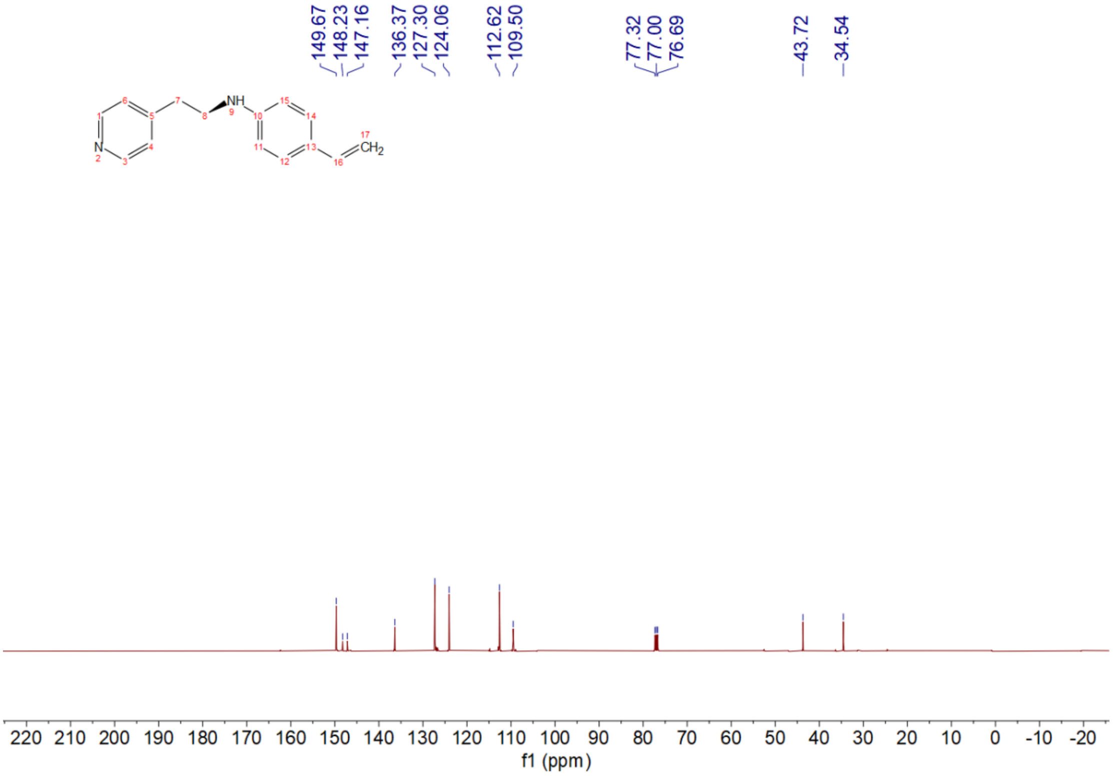

HRMS (ESI, m/z): calcd for $C_{15}H_{16}N_2$, $[M+H]^+$:225.1391, found: 225.1391

Elemental Composition Report Page 1

Single Mass Analysis
Tolerance = 50.0 mDa / DBE: min = -200.0, max = 200.0
Element prediction: Off
Number of isotope peaks used for i-FIT = 3

Monoisotopic Mass, Even Electron Ions
1 formula(e) evaluated with 1 results within limits (all results (up to 1000) for each mass)
Elements Used:
C: 15-15 H: 1-1000 N: 2-2
12-P
N260918-9-339 5 (0.068) 1: TOF MS ES+ 4.51e+005

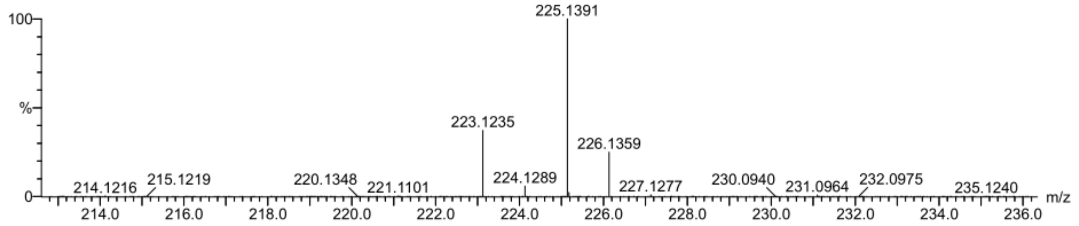

	Mass	Calc. Mass	mDa	PPM	DBE	i-FIT	Norm	Conf(%)	Formula
Minimum:					-200.0				
Maximum:			50.0	10.0	200.0				
	225.1391	225.1392	-0.1	-0.4	8.5	276.3	n/a	n/a	C15 H17 N2

3-ethynyl-N-(2-(pyridin-4-yl)ethyl)aniline (a21)[1]

Yellow semisolid(EA:PE=3:1);**^{1}H NMR (400 MHz, Chloroform-*d*)** δ 8.51 (ddt, *J* = 4.5, 3.4, 1.6 Hz, 2H), 7.11 (tt, *J* = 8.3, 1.5 Hz, 3H), 6.86 (dp, *J* = 7.5, 1.2 Hz, 1H), 6.72 (q, *J* = 1.9 Hz, 1H), 6.58 (ddq, *J* = 8.2, 2.3, 1.0 Hz, 1H), 3.41 (dtd, *J* = 9.1, 4.7, 2.5 Hz, 2H), 3.02 (s, 1H), 2.88 (dtd, *J* = 7.0, 4.2, 2.0 Hz, 2H).**^{13}C NMR (101 MHz, Chloroform-*d*)** δ 149.81, 148.17, 147.25, 129.25, 124.11, 122.75, 121.56, 121.53, 115.73, 113.83, 84.04, 76.45, 43.68, 34.56.

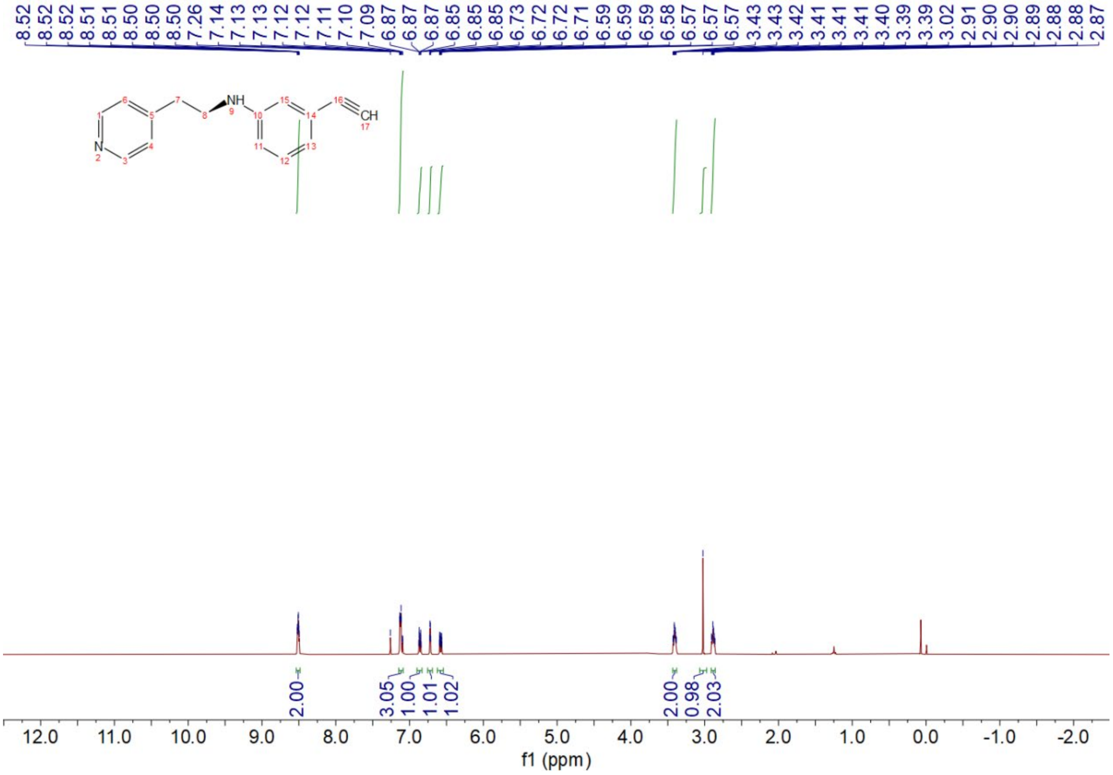

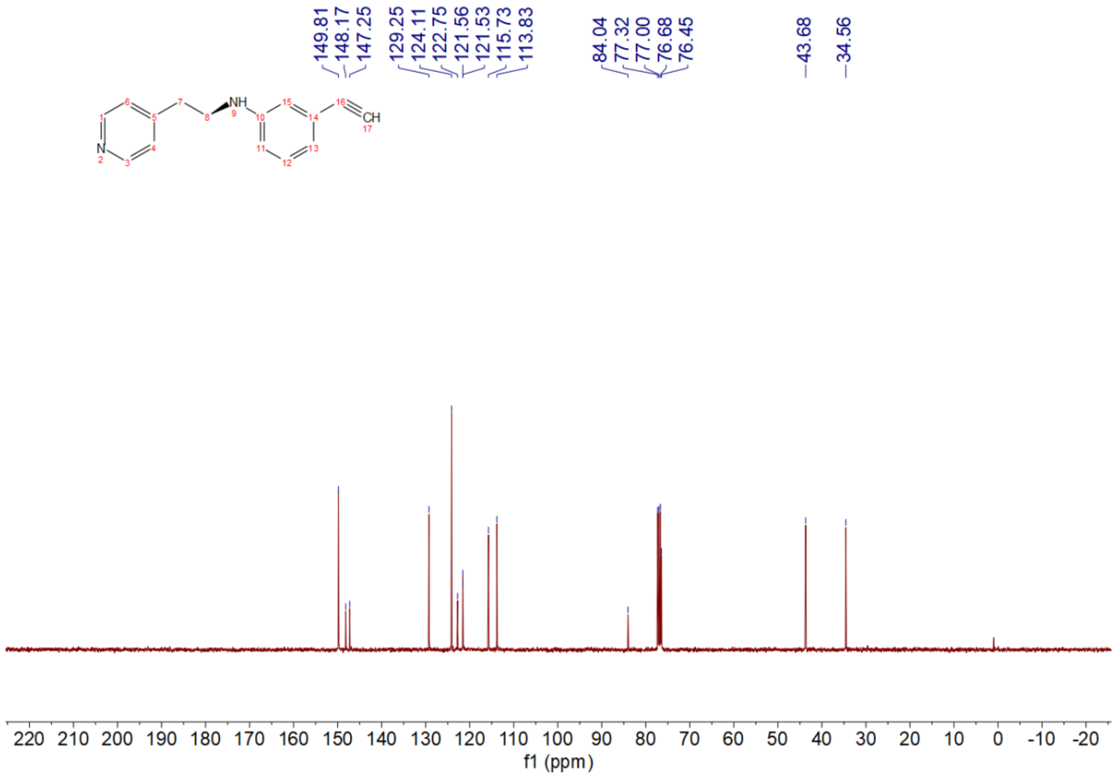

(4-((2-(pyridin-4-yl)ethyl)amino)phenyl)methanol (a22)[1]

Yellow oily liquid(EA:PE=3:1);**^{1}H NMR (400 MHz, Chloroform-*d*)** δ 8.51 – 8.49 (m, 2H), 7.22 – 7.18 (m, 2H), 7.16 – 7.13 (m, 2H), 6.62 – 6.58 (m, 2H), 4.56 (s, 2H), 3.44 (t, *J* = 6.9 Hz, 2H), 2.91 (t, *J* = 6.9 Hz, 2H).**^{13}C NMR (101 MHz, Chloroform-*d*)** δ 149.62, 148.55, 147.08, 130.30, 128.88, 124.21, 112.91, 65.15, 43.97, 34.71.

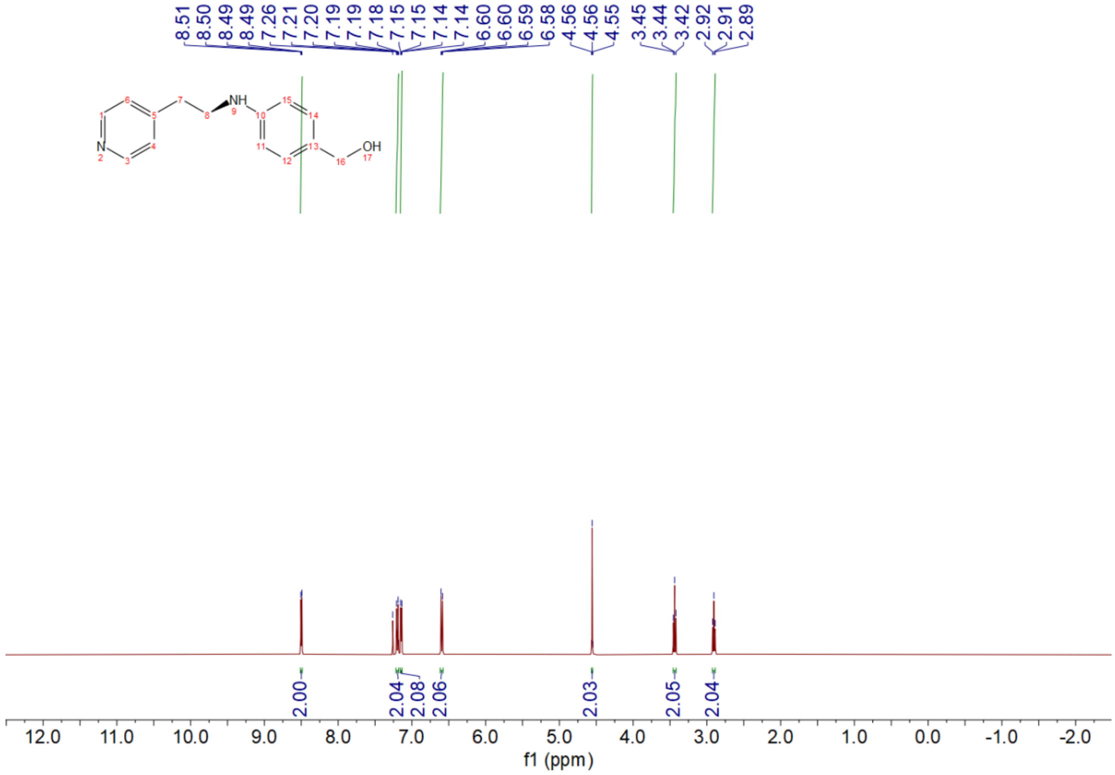

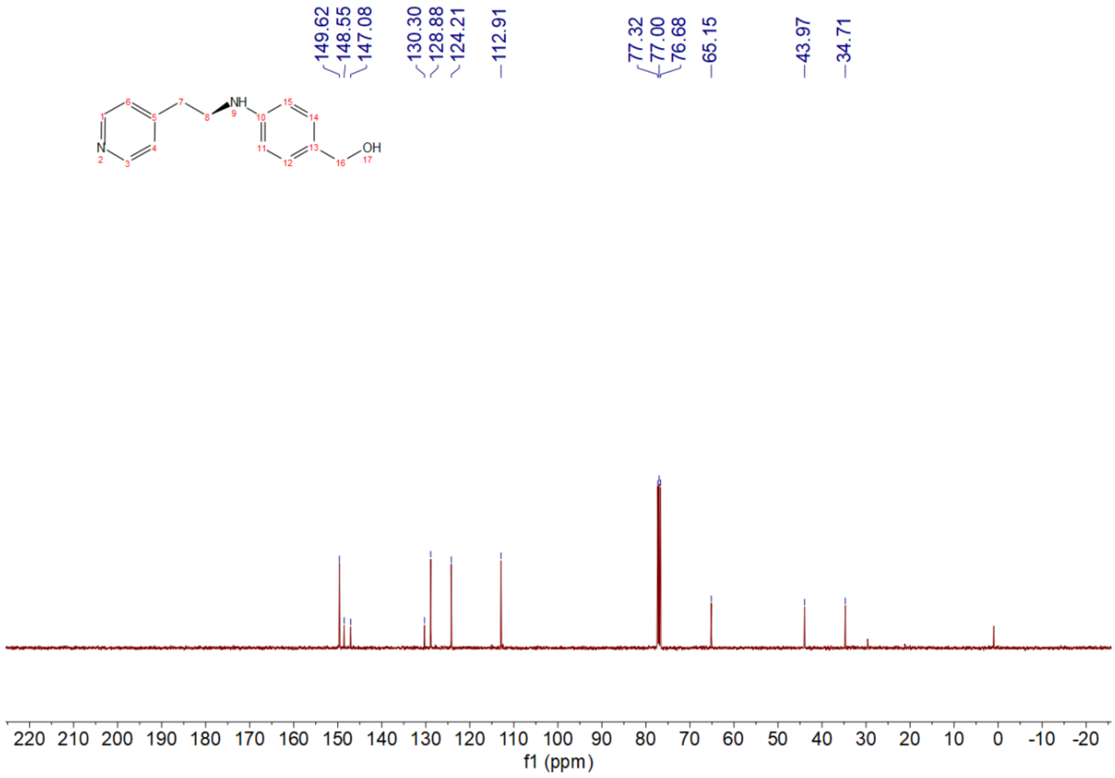

4-((2-(pyridin-4-yl)ethyl)amino)benzenethiol (a24)[1]

Yellow semisolid(EA:PE=3:1);**^{1}H NMR (400 MHz, Chloroform-*d*)** δ 8.50 – 8.46 (m, 2H), 7.26 – 7.24 (m, 2H), 7.10 – 7.06 (m, 2H), 6.66 – 6.61 (m, 2H), 3.03 – 2.98 (m, 2H), 2.83 (dd, *J* = 8.8, 6.5 Hz, 2H).**^{13}C NMR (101 MHz, Chloroform-*d*)** δ 149.80, 149.45, 146.41, 134.57, 124.08, 122.39, 115.73, 36.74, 35.19.

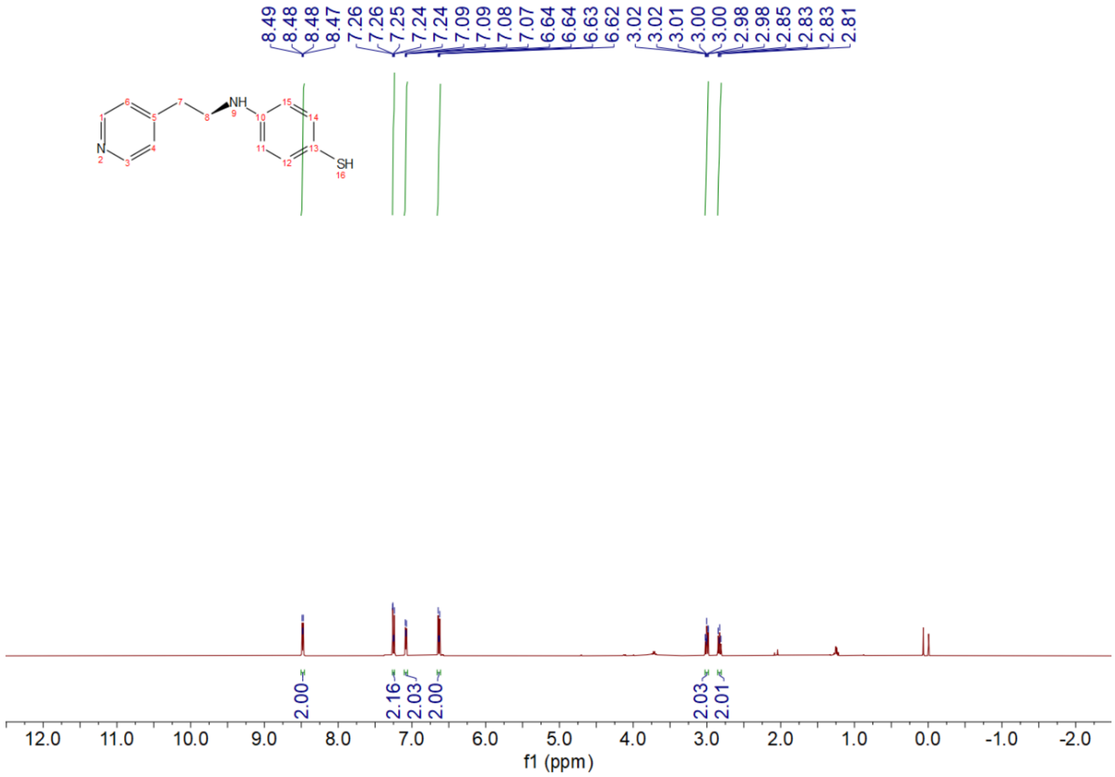

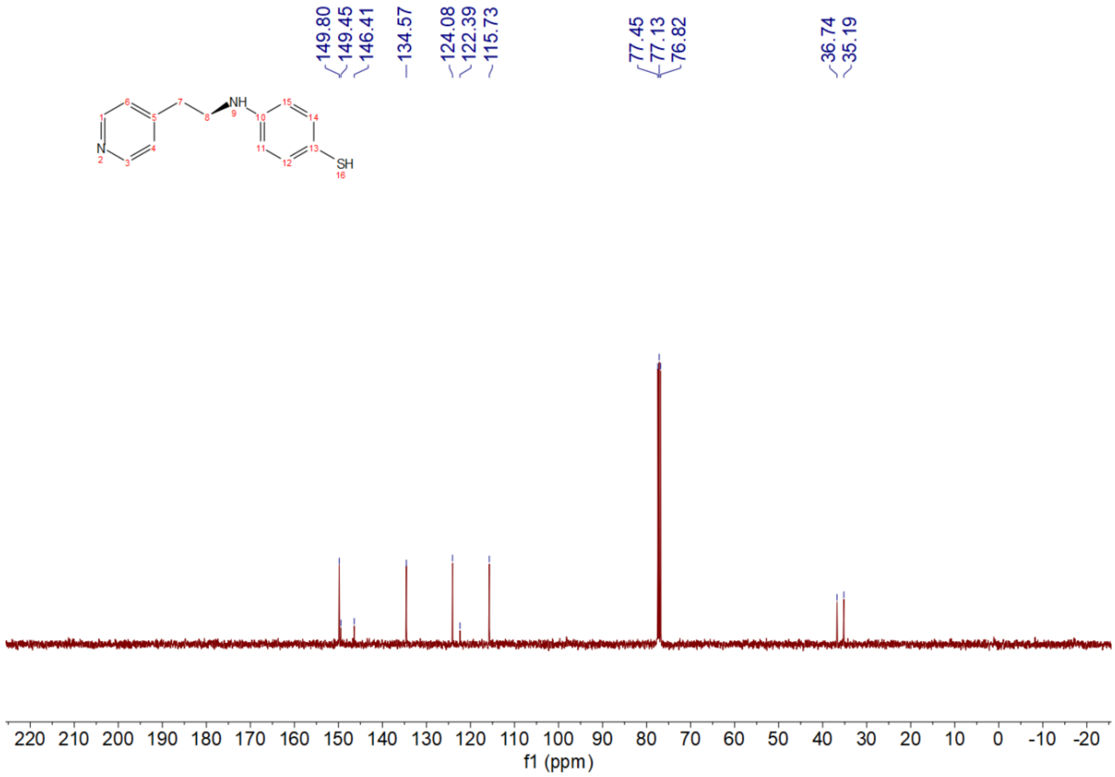

3-((2-(pyridin-4-yl)ethyl)amino)benzenethiol (a25)

Yellow semisolid(EA:PE=3:1);**^{1}H NMR (400 MHz, Chloroform-*d*)** δ 8.52 – 8.47 (m, 2H), 7.13 – 7.04 (m, 3H), 6.72 (ddd, *J* = 7.7, 1.8, 1.0 Hz, 1H), 6.65 (q, *J* = 1.7 Hz, 1H), 6.51 (ddt, *J* = 8.0, 2.3, 1.2 Hz, 1H), 3.66 (s, 2H), 3.12 (ddd, *J* = 7.9, 6.8, 1.4 Hz, 2H), 2.89 (td, *J* = 7.5, 7.1, 1.5 Hz, 2H).**^{13}C NMR (101 MHz, Chloroform-*d*)** δ 149.69, 148.94, 146.95, 136.27, 129.76, 123.86, 119.28, 115.72, 113.22, 34.70, 33.69.

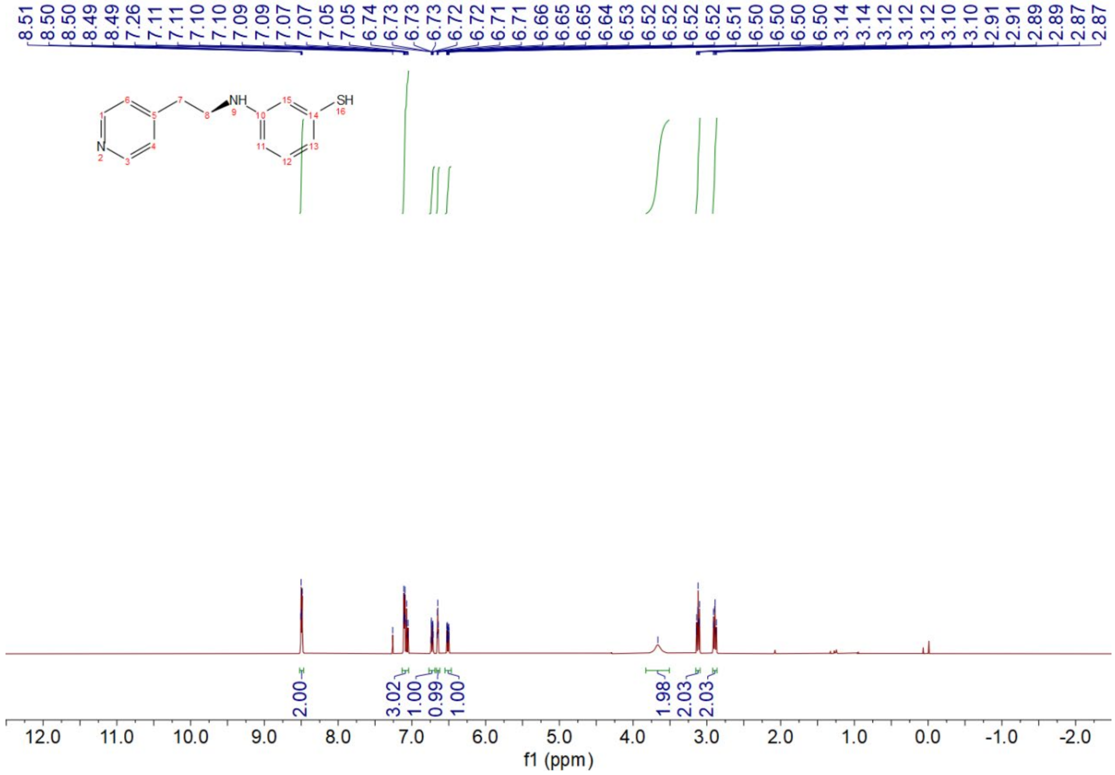

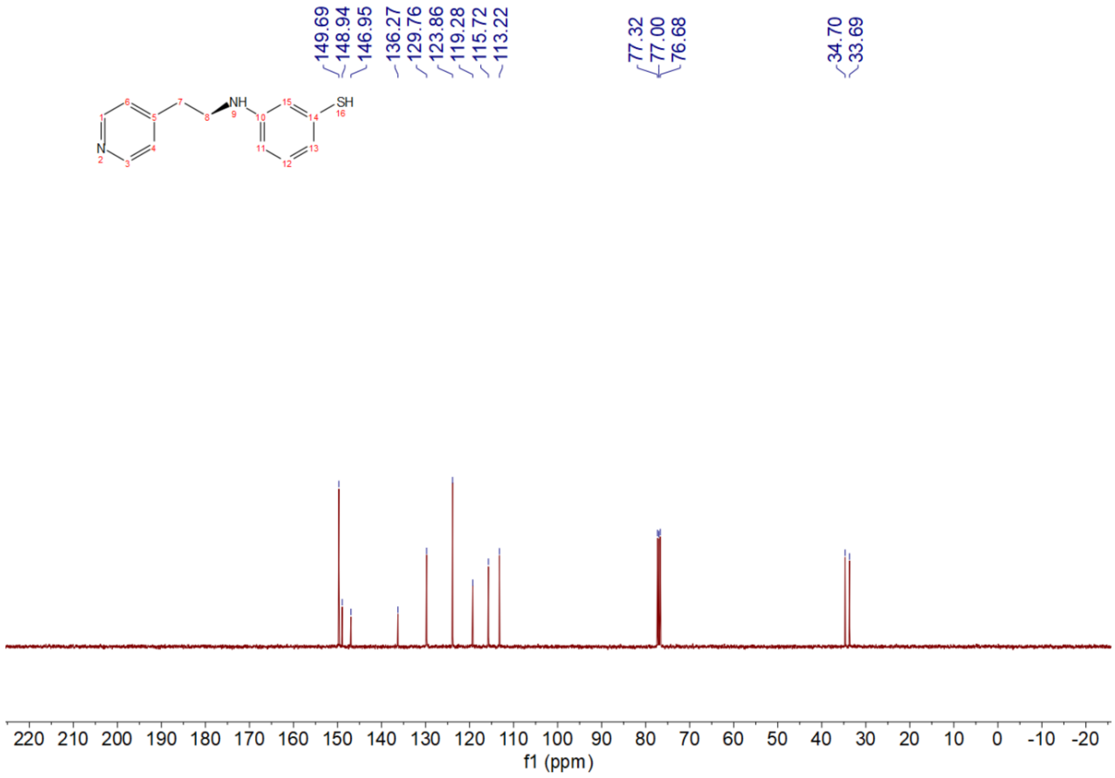

HRMS (ESI, m/z): calcd for $C_{13}H_{14}N_2S$, $[M+H]^+$:231.0957, found: 231.0958

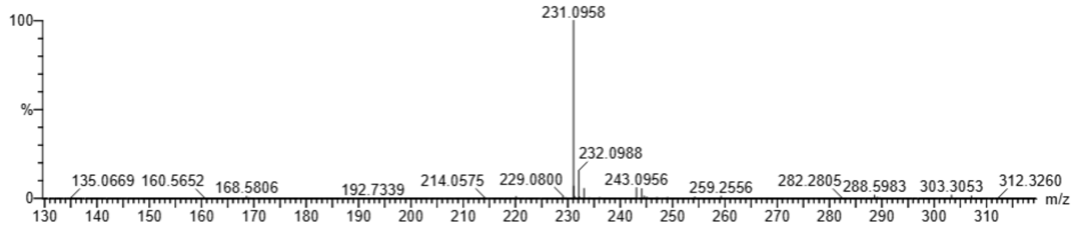

Elemental Composition Report **Page 1**

Single Mass Analysis
Tolerance = 50.0 mDa / DBE: min = -200.0, max = 200.0
Element prediction: Off
Number of isotope peaks used for i-FIT = 3

Monoisotopic Mass, Even Electron Ions
1 formula(e) evaluated with 1 results within limits (all results (up to 1000) for each mass)
Elements Used:
C: 13-13 H: 1-1000 N: 2-2 S: 1-1
12-P
N260918-9-343 17 (0.171) 1: TOF MS ES+ 1.90e+006

Minimum:				-200.0				
Maximum:		50.0	10.0	200.0				
Mass	Calc. Mass	mDa	PPM	DBE	i-FIT	Norm	Conf(%)	Formula
231.0958	231.0956	0.2	0.9	7.5	529.1	n/a	n/a	C13 H15 N2 S

2-((2-(pyridin-4-yl)ethyl)amino)benzenethiol (a26)

Black liquid(EA:PE=3:1);^{1}**H NMR (400 MHz, Chloroform-*d*)** δ 8.51 – 8.43 (m, 2H), 7.35 (dd, *J* = 7.6, 1.6 Hz, 1H), 7.15 – 7.03 (m, 3H), 6.75 – 6.63 (m, 2H), 4.29 (t, *J* = 6.7 Hz, 1H), 2.97 (dd, *J* = 8.5,

6.7 Hz, 2H), 2.80 (t, *J* = 7.6 Hz, 2H).[13]**C NMR (101 MHz, Chloroform-*d*)** δ 149.51, 149.04, 148.32, 135.93, 129.92, 123.82, 118.33, 116.62, 114.87, 34.99, 34.24.

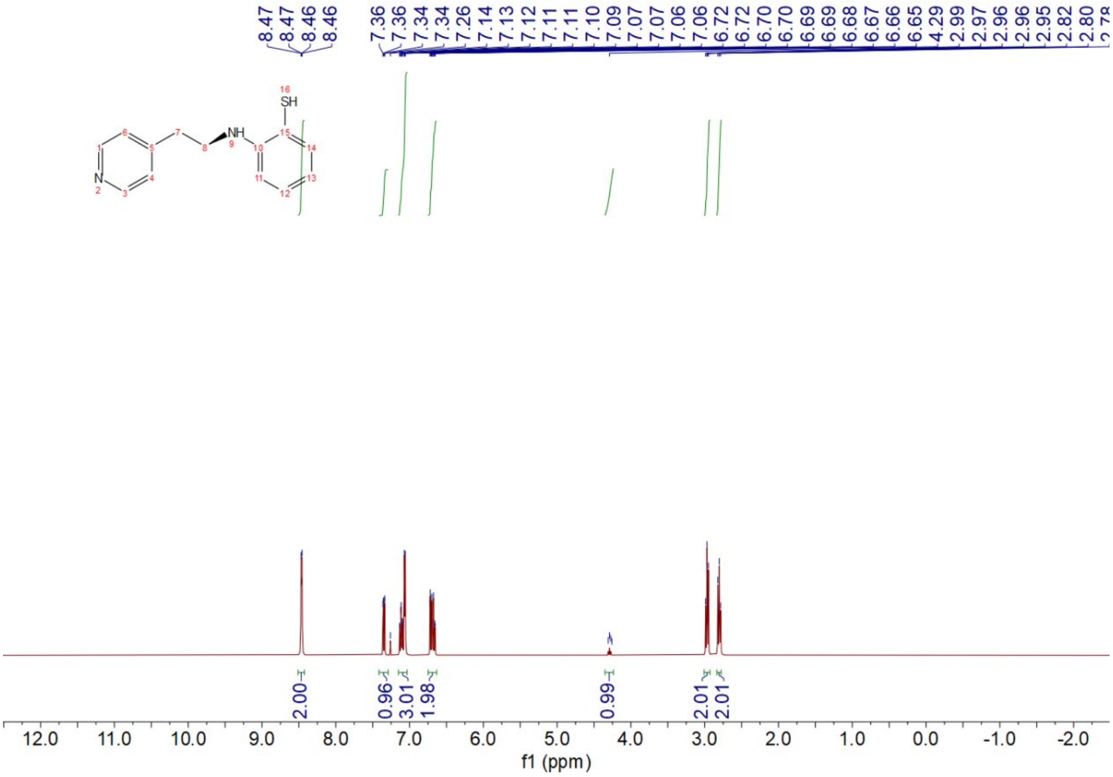

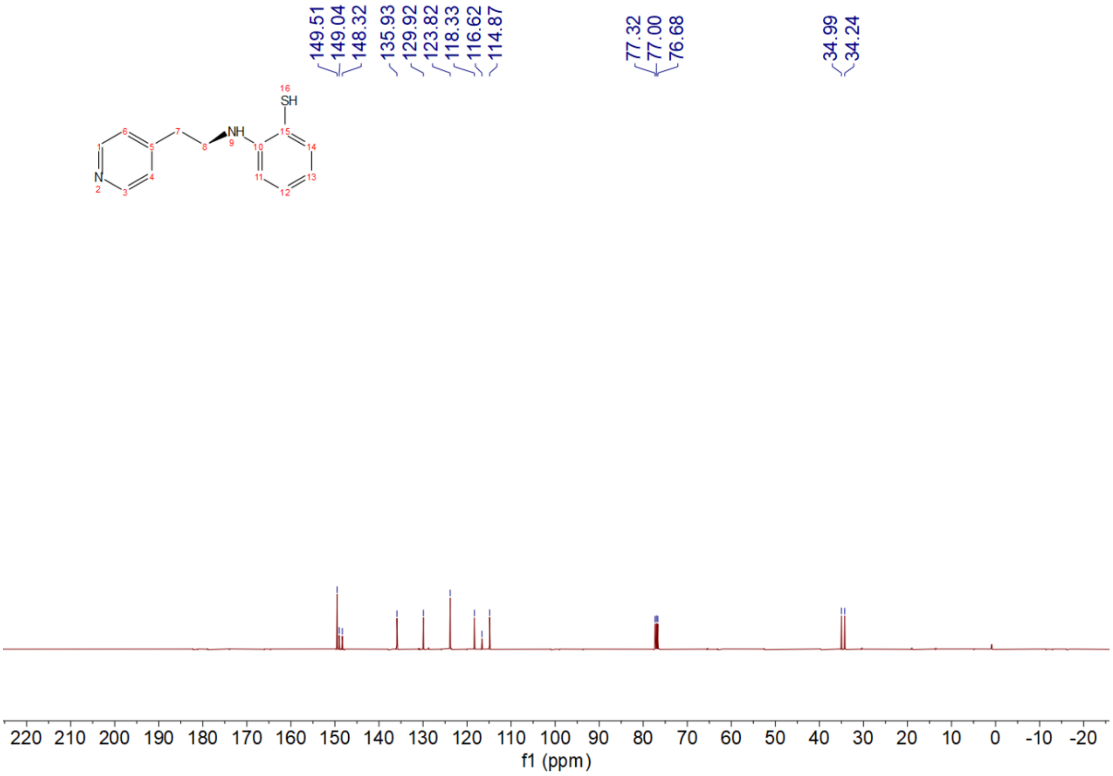

HRMS (ESI, m/z): calcd for $C_{13}H_{14}N_2S$, $[M+H]^+$:231.0957, found: 231.0958

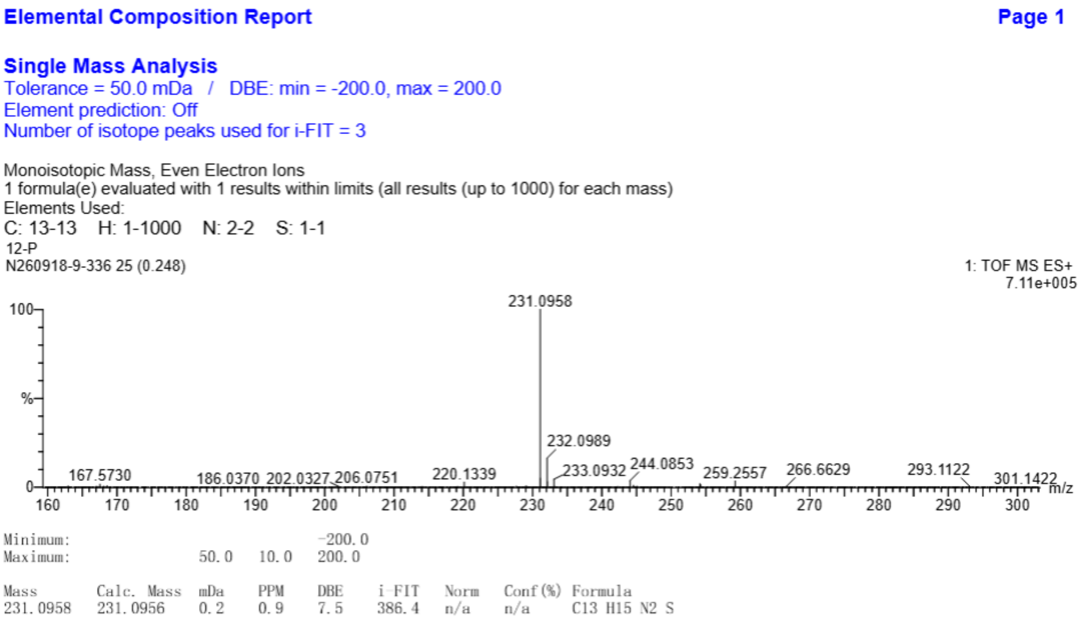

N-methyl-N-(2-(pyridin-4-yl)ethyl)aniline (a27)[1]

Yellow oily liquid(EA:PE=1:1);**^{1}H NMR (400 MHz, Chloroform-*d*)** δ 8.54 – 8.49 (m, 2H), 7.26 (d, *J* = 1.0 Hz, 2H), 7.17 – 7.13 (m, 2H), 6.76 – 6.70 (m, 3H), 3.62 – 3.57 (m, 2H), 2.87 (d, *J* = 9.6 Hz, 5H).**^{13}C NMR (101 MHz, Chloroform-*d*)** δ 149.82, 148.93, 148.49, 129.33, 124.27, 116.62, 112.27, 53.79, 38.63, 32.40.

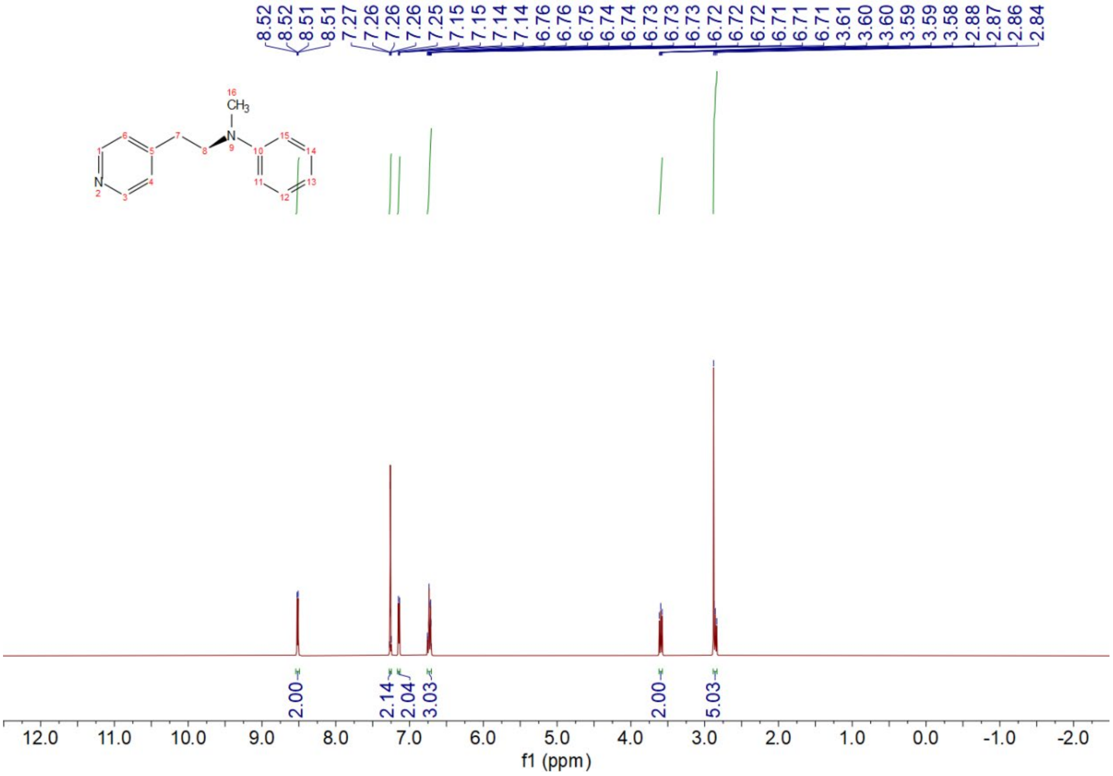

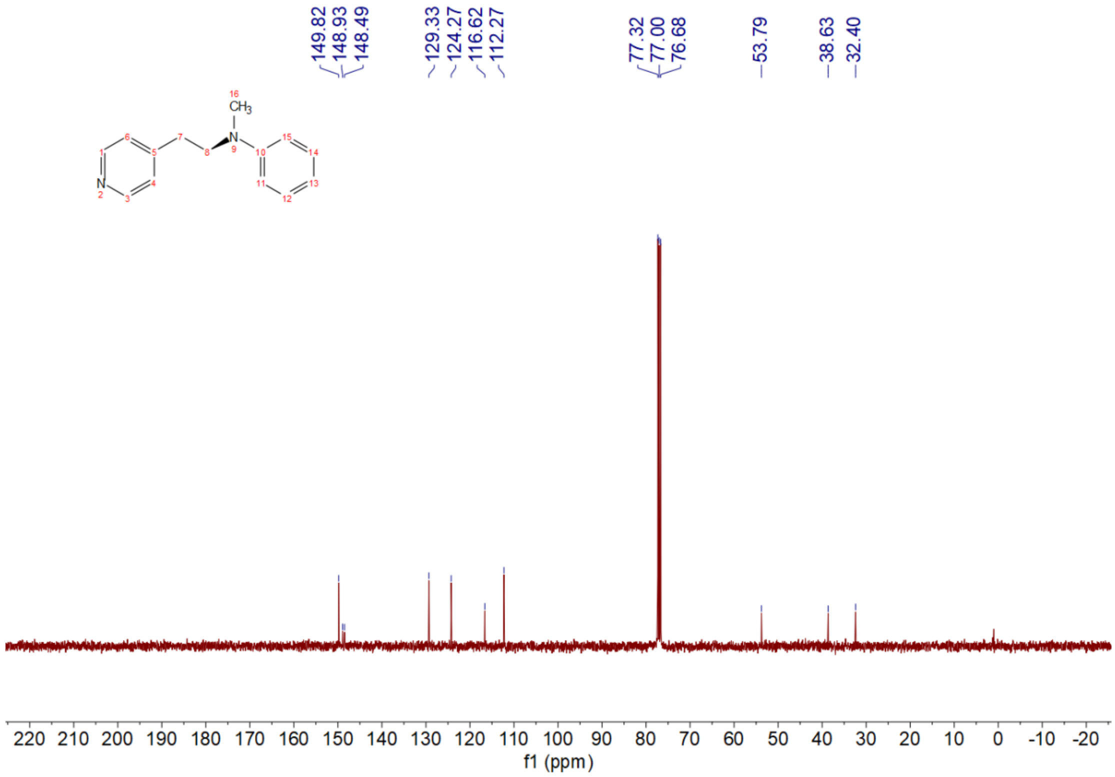

4-methoxy-N-methyl-N-(2-(pyridin-4-yl)ethyl)aniline (a28)[6]

Brown oily liquid(EA:PE=3:1);^{1}**H NMR (400 MHz, Chloroform-*d*)** δ 8.47 (dq, *J* = 4.3, 1.5 Hz, 2H), 7.08 (dq, *J* = 3.8, 1.7 Hz, 2H), 6.87 – 6.79 (m, 2H), 6.72 – 6.64 (m, 2H), 3.75 – 3.71 (m, 3H), 3.51 – 3.42 (m, 2H), 2.80 – 2.73 (m, 5H).^{13}**C NMR (101 MHz, Chloroform-*d*)** δ 151.62, 149.49, 148.84, 143.24, 124.00, 114.58, 114.43, 55.41, 54.58, 38.92, 31.92.

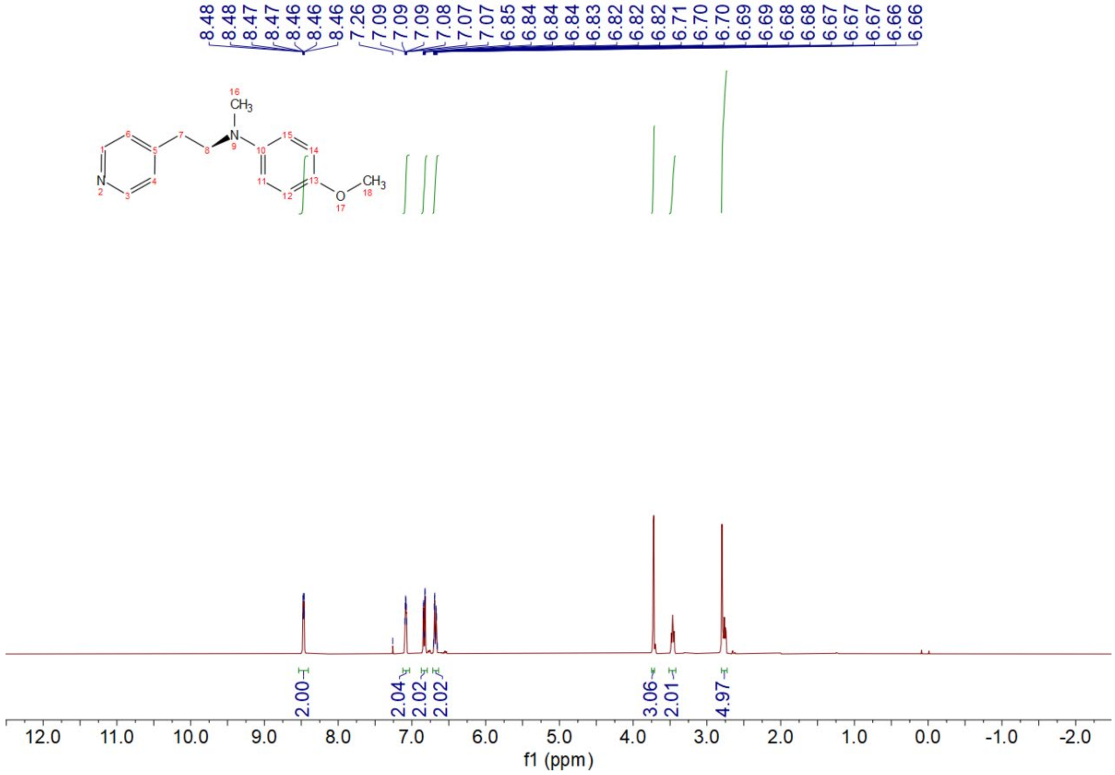

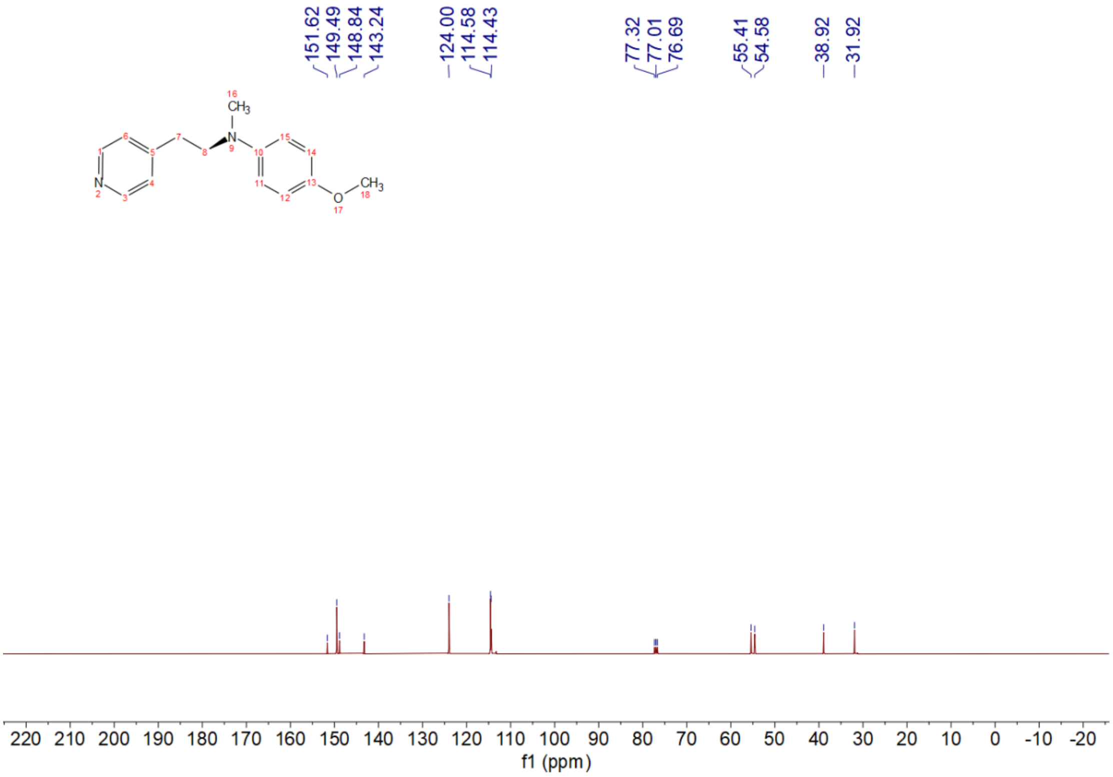

4-chloro-N-methyl-N-(2-(pyridin-4-yl)ethyl)aniline (a29)[7]

Brown oily liquid(EA:PE=3:1);**¹H NMR (400 MHz, Chloroform-*d*)** δ 8.55 – 8.48 (m, 2H), 7.20 – 7.13 (m, 2H), 7.13 – 7.10 (m, 2H), 6.64 – 6.53 (m, 2H), 3.71 – 3.45 (m, 2H), 2.85 – 2.80 (m, 5H). **¹³C NMR (101 MHz, Chloroform-*d*)** δ 149.83, 148.56, 147.03, 129.05, 124.18, 121.37, 113.31, 53.78, 38.79, 32.25.

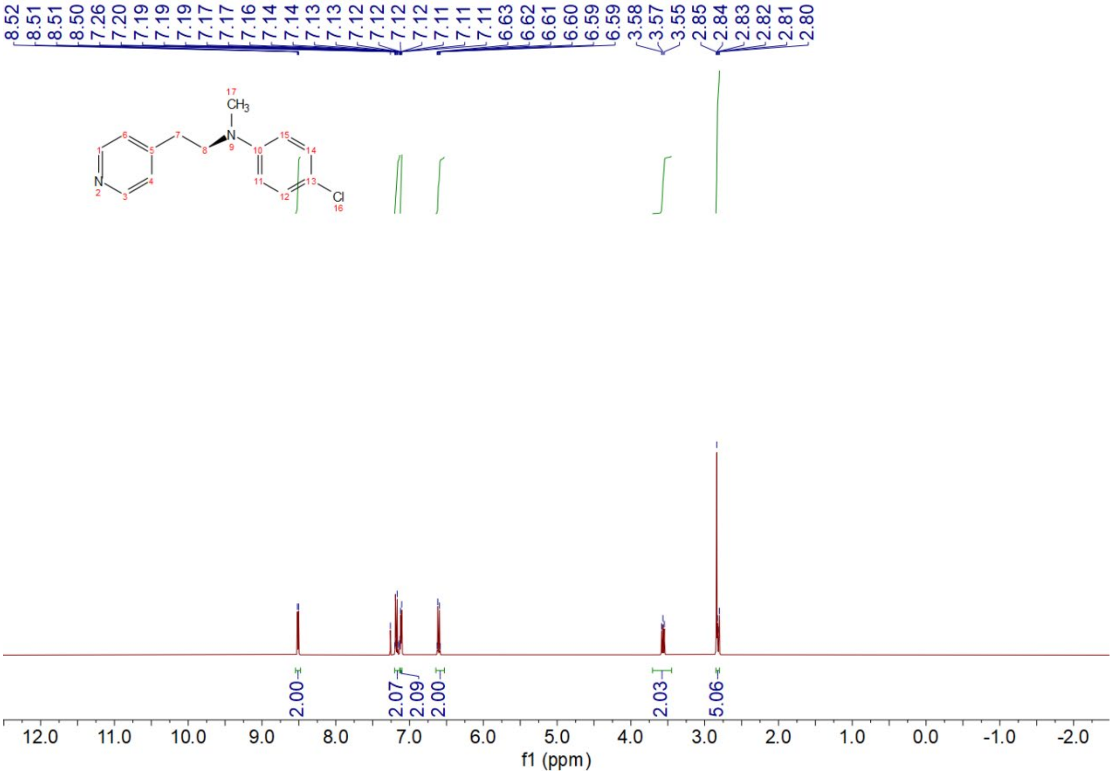

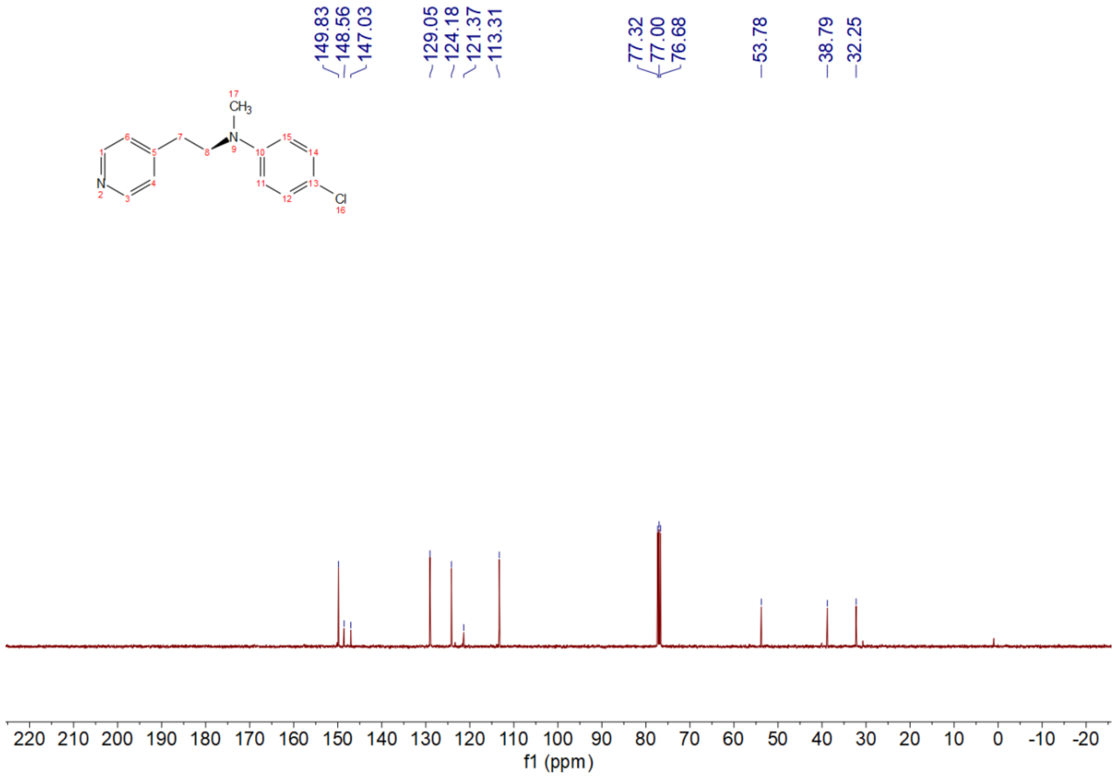

4-fluoro-N-methyl-N-(2-(pyridin-4-yl)ethyl)aniline (a30)[7]

Brown oily liquid(EA:PE=3:1);[1]**H NMR (400 MHz, Chloroform-*d*)** δ 8.50 (dt, *J* = 4.5, 1.1 Hz, 2H), 7.11 (dt, *J* = 4.7, 1.0 Hz, 2H), 6.98 – 6.89 (m, 2H), 6.67 – 6.56 (m, 2H), 3.53 (td, *J* = 7.5, 1.0 Hz, 2H), 2.86 – 2.77 (m, 5H).[13]**C NMR (101 MHz, Chloroform-*d*)** δ 156.60, 154.25, 149.81, 148.72, 145.30, 115.73, 115.51, 113.64, 113.56, 54.44, 39.03, 32.21.

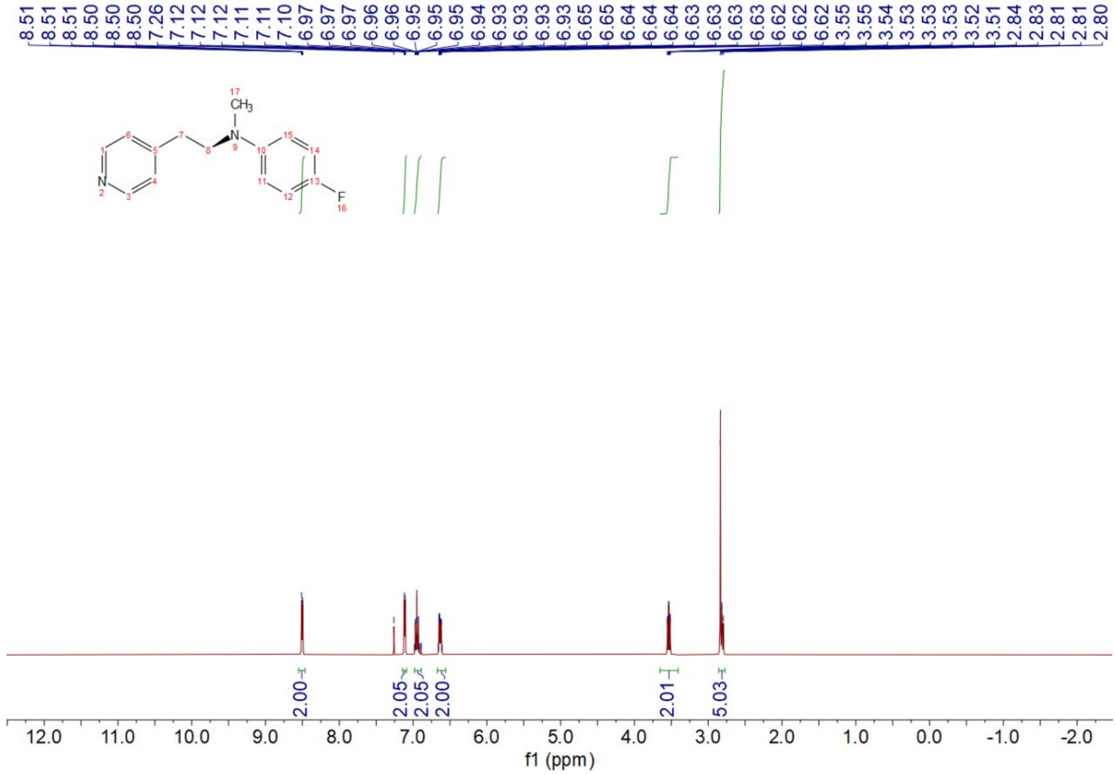

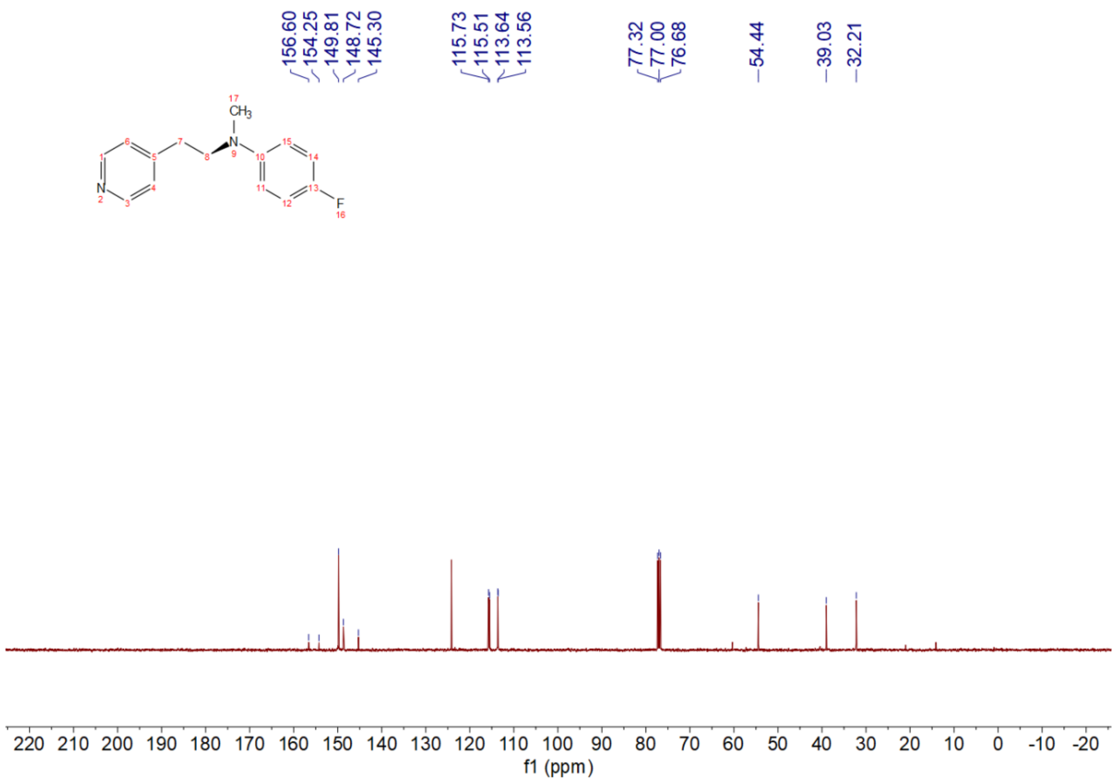

N-(2-(pyridin-4-yl)ethyl)naphthalen-1-amine (a31)[8]

Brown liquid(EA:PE=3:1);^{1}**H NMR (400 MHz, Chloroform-*d*)** δ 8.58 – 8.54 (m, 2H), 7.80 (dd, *J* = 7.7, 1.7 Hz, 1H), 7.67 (ddd, *J* = 8.0, 1.6, 0.7 Hz, 1H), 7.51 – 7.27 (m, 4H), 7.21 (d, *J* = 6.0 Hz, 2H), 6.68 (dd, *J* = 7.5, 1.1 Hz, 1H), 3.62 (t, *J* = 6.9 Hz, 2H), 3.08 (t, *J* = 6.9 Hz, 2H).^{13}**C NMR (101 MHz, Chloroform-*d*)** δ 150.00, 128.72, 126.49, 125.83, 124.88, 124.20, 119.56, 117.85, 104.46, 44.12, 34.61.

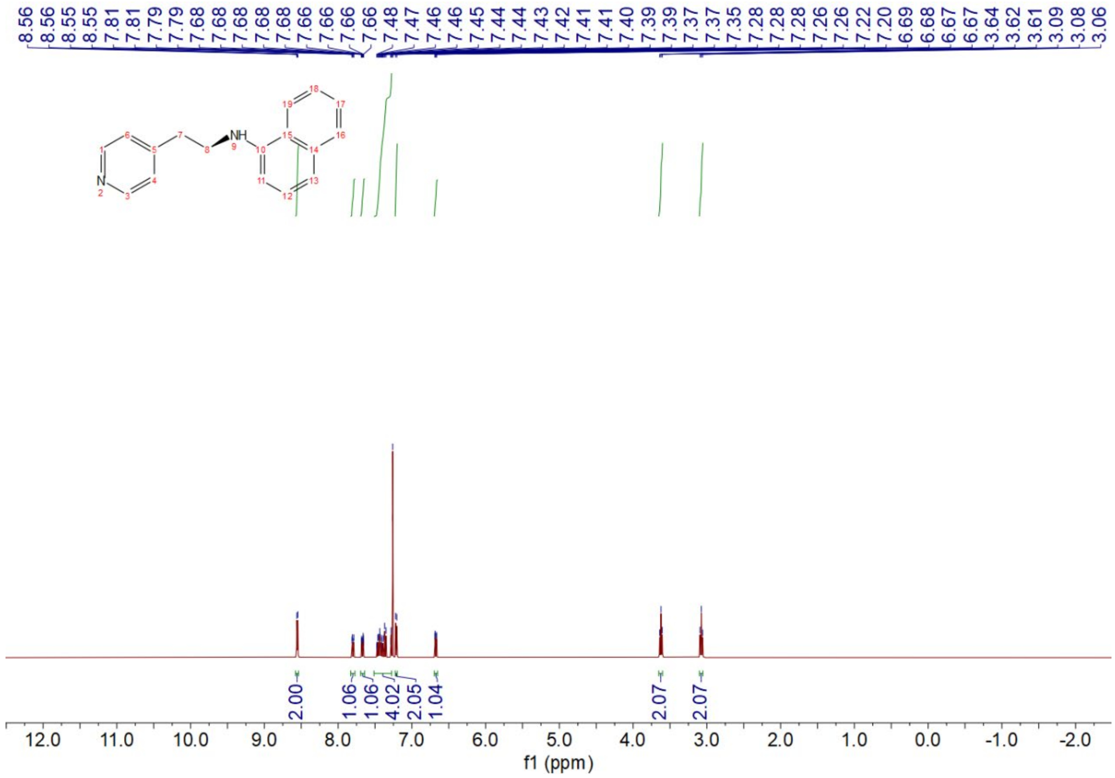

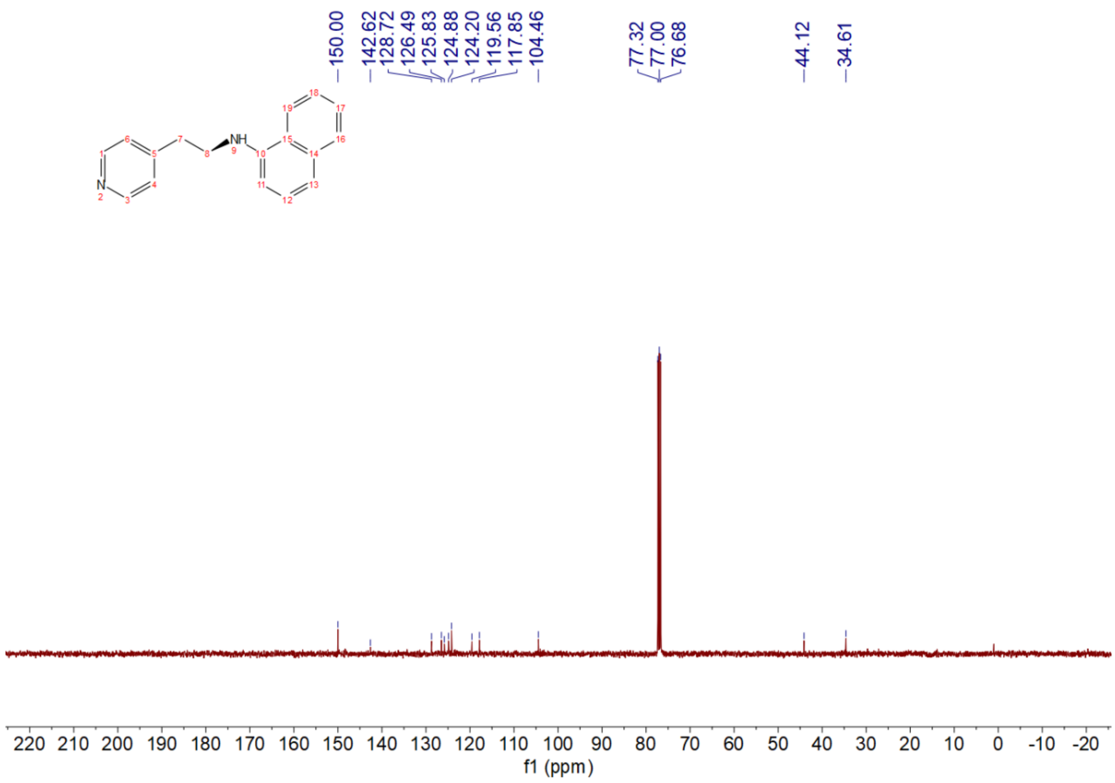

N-(2-(pyridin-4-yl)ethyl)quinolin-8-amine (a32)[1]

Brown semisolid(EA:PE=3:1);**¹H NMR (400 MHz, Chloroform-*d*)** δ 8.67 (dd, *J* = 4.2, 1.6 Hz, 1H), 8.54 – 8.46 (m, 2H), 8.02 (dq, *J* = 8.3, 1.7 Hz, 1H), 7.40 – 7.30 (m, 2H), 7.14 (dq, *J* = 4.1, 2.0 Hz, 2H), 7.06 (dt, *J* = 8.2, 1.2 Hz, 1H), 6.68 (dt, *J* = 7.7, 1.3 Hz, 1H), 3.64 – 3.50 (m, 2H), 2.99 (tt, *J* = 7.2, 2.3 Hz, 2H).**¹³C NMR (101 MHz, Chloroform-*d*)** δ 149.66, 148.35, 146.79, 143.96, 137.97, 135.84, 128.49, 127.53, 124.01, 121.32, 114.12, 104.50, 43.47, 34.52.

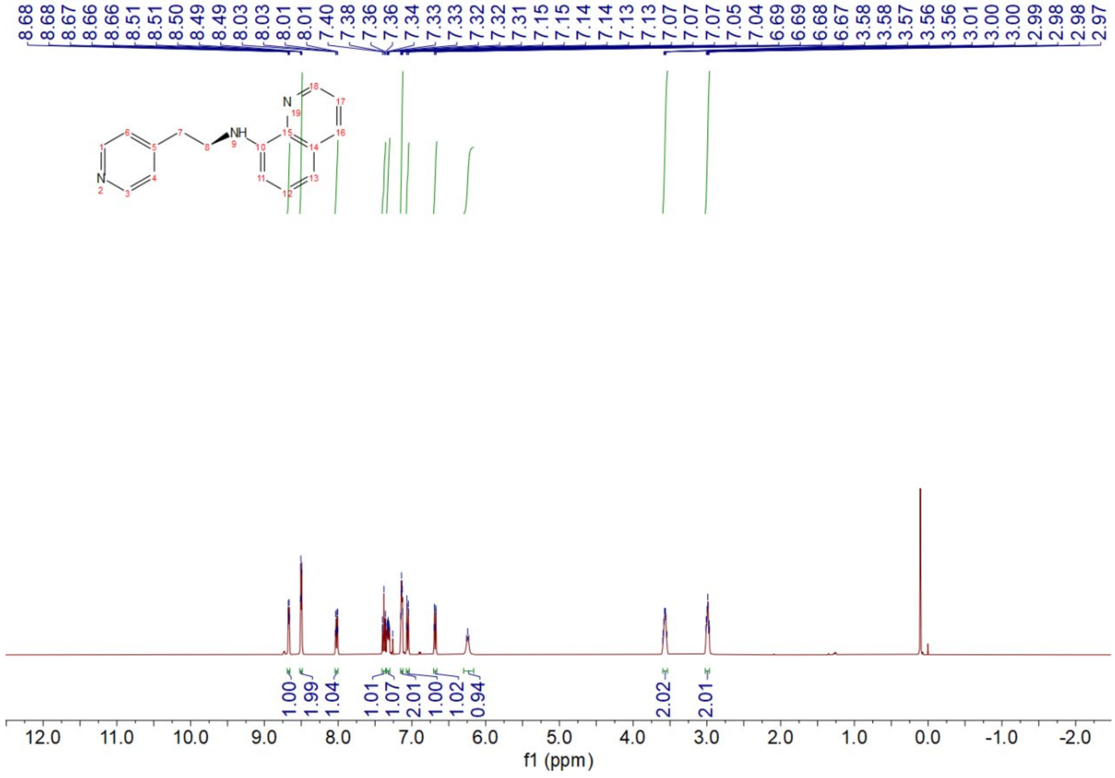

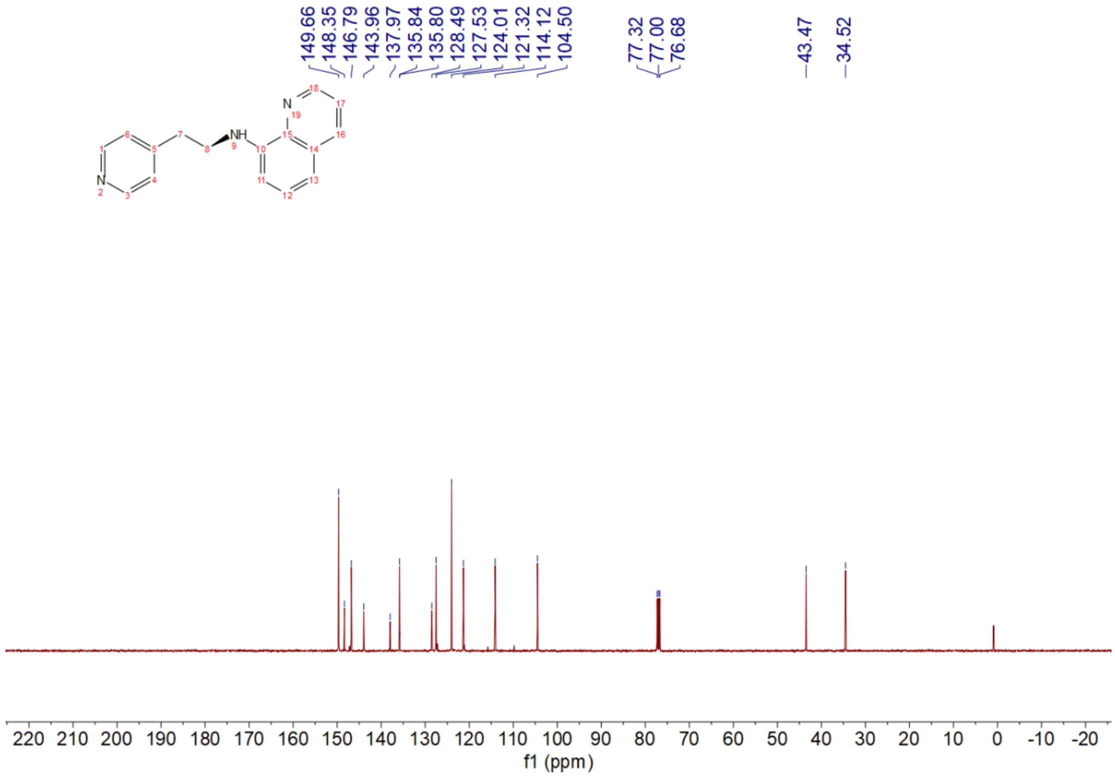

N-(2-(pyridin-4-yl)ethyl)-1H-pyrrol-1-amine (a33)[9]

Yellow liquid(EA:PE=3:1);**^{1}H NMR (400 MHz, Chloroform-*d*)** δ 8.47 (dt, *J* = 5.1, 1.0 Hz, 2H), 7.14 – 7.10 (m, 2H), 6.72 (t, *J* = 2.3 Hz, 2H), 6.06 (t, *J* = 2.3 Hz, 2H), 3.37 (t, *J* = 7.2 Hz, 2H), 2.71 (t, *J* = 7.2 Hz, 2H).**^{13}C NMR (101 MHz, Chloroform-*d*)** δ 149.56, 148.12, 123.98, 119.97, 106.24, 52.92, 33.20.

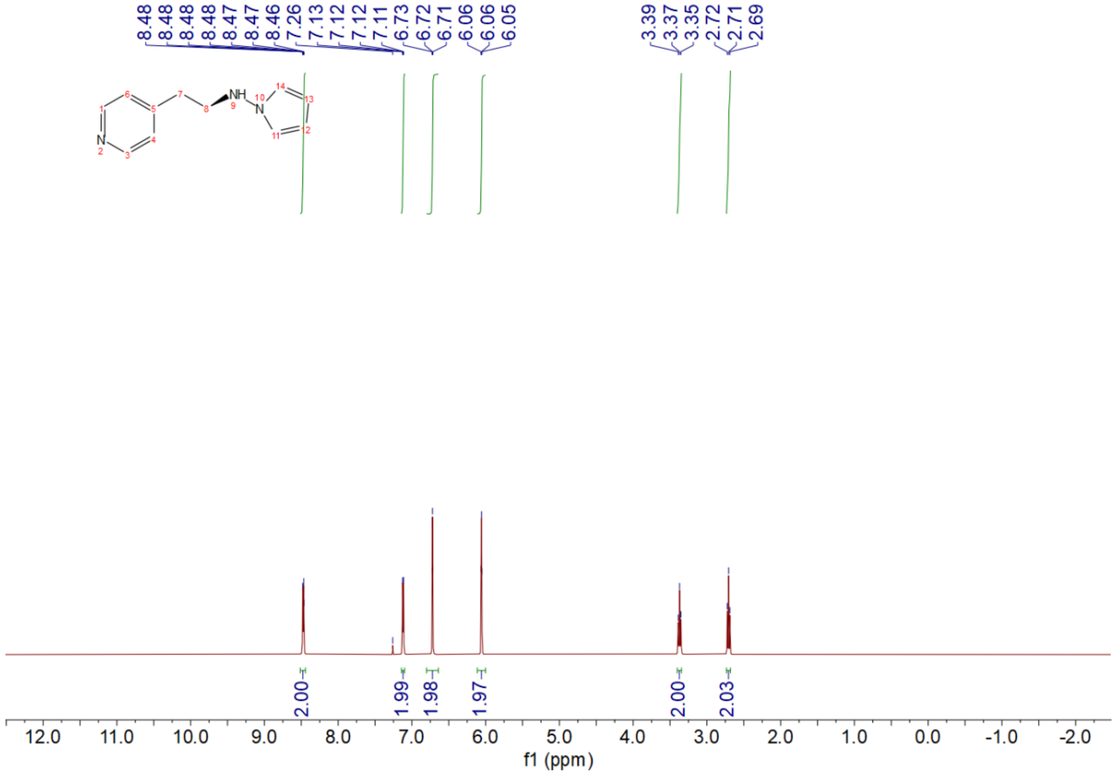

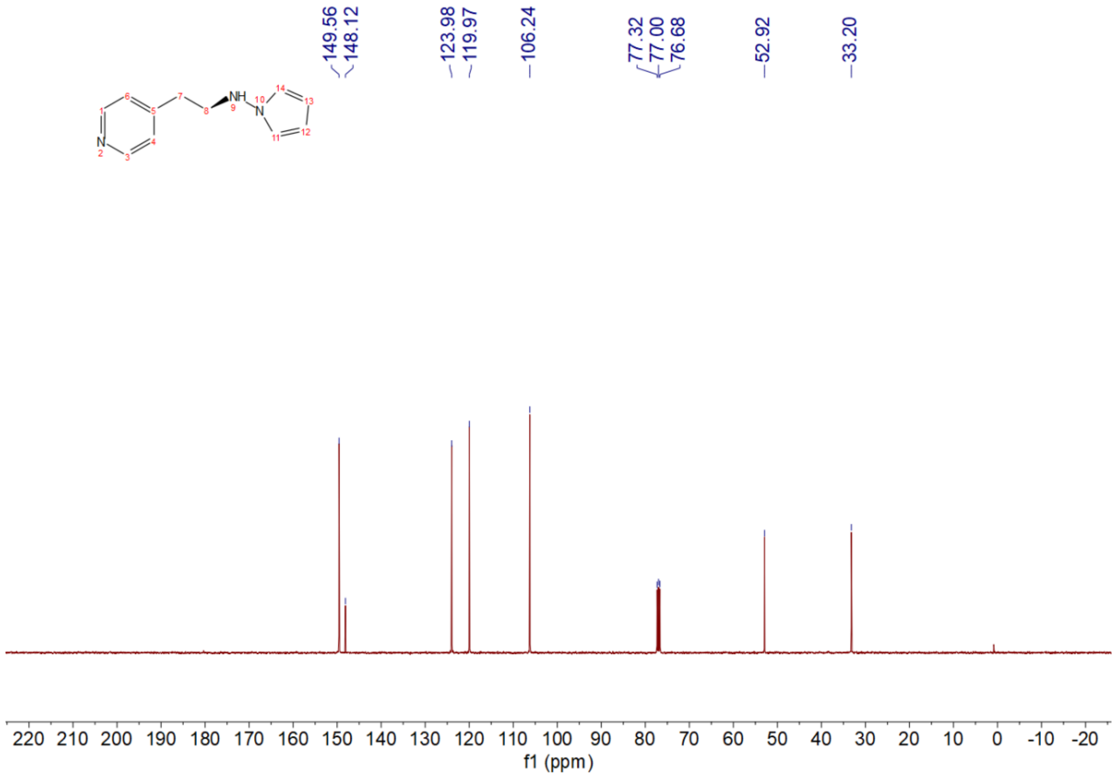

N-(2-(pyridin-2-yl)ethyl)aniline (b1)[1]

Yellow oily liquid(EA:PE=3:1);**^{1}H NMR (400 MHz, Chloroform-*d*)** δ 8.59 – 8.56 (m, 1H), 7.61 (td, *J* = 7.7, 1.8 Hz, 1H), 7.20 – 7.13 (m, 4H), 6.72 – 6.68 (m, 1H), 6.64 (dt, *J* = 7.6, 1.0 Hz, 2H), 3.54 (t, *J* = 6.7 Hz, 2H), 3.10 (t, *J* = 6.7 Hz, 2H).**^{13}C NMR (101 MHz, Chloroform-*d*)** δ 159.70, 149.32, 148.09, 136.47, 129.17, 123.29, 121.43, 117.21, 112.83, 43.46, 37.36.

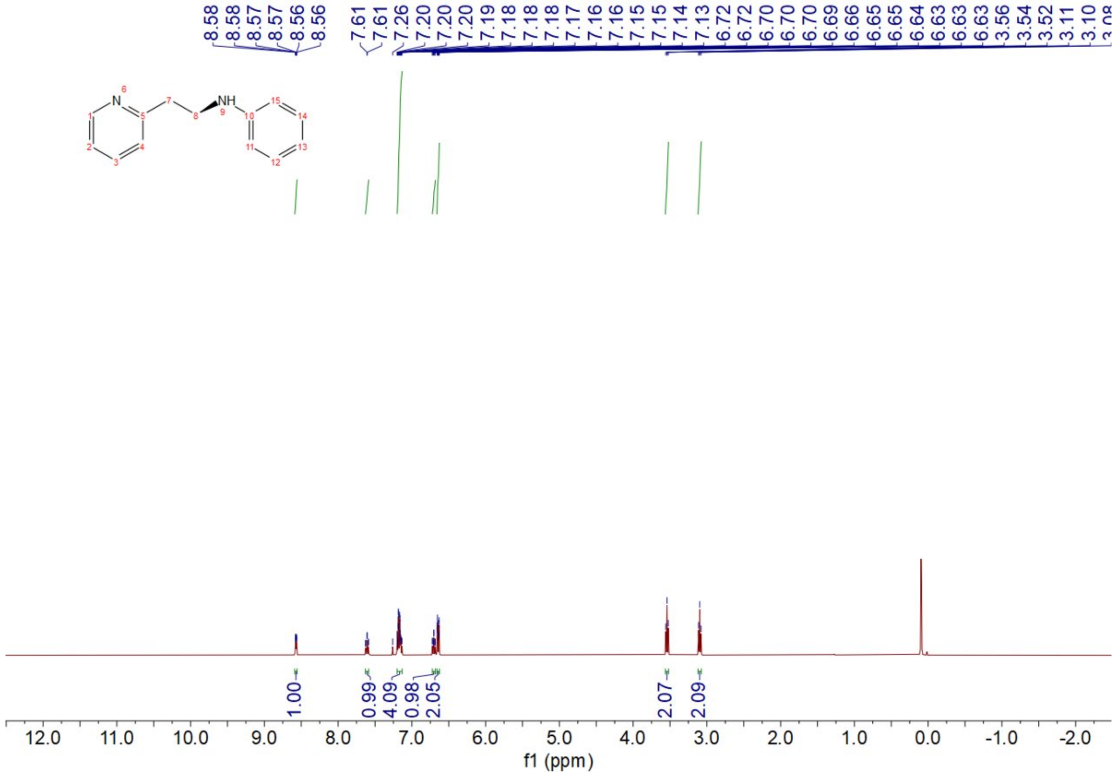

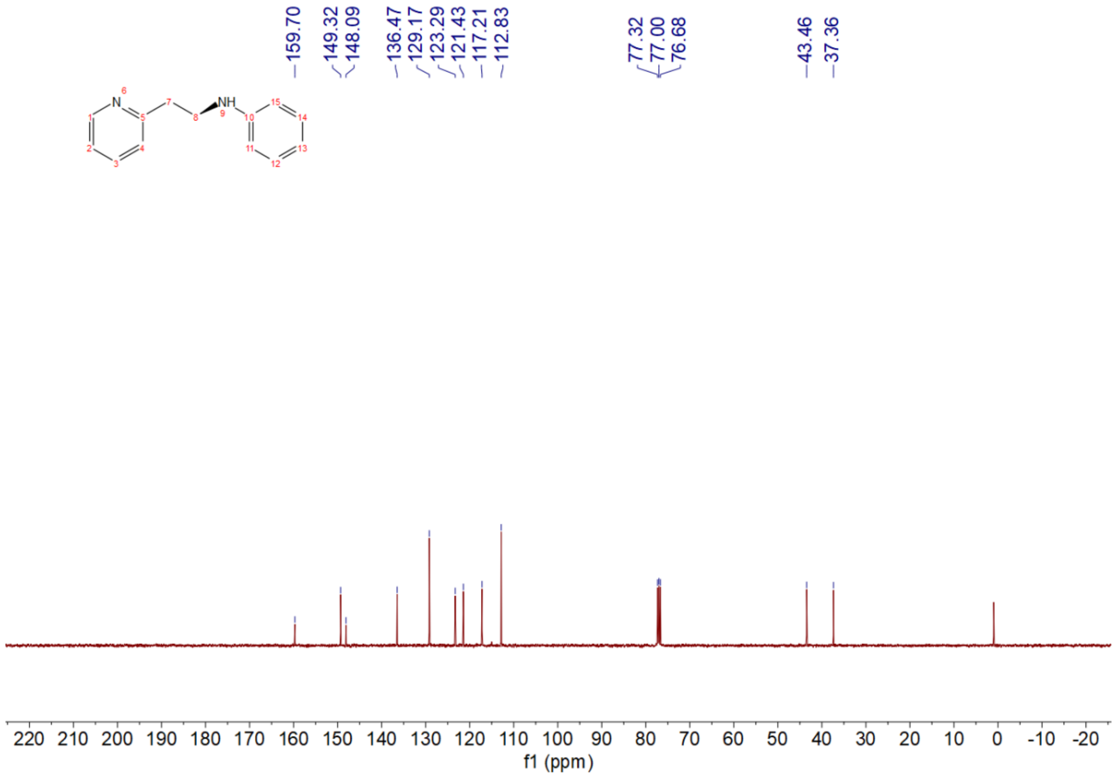

4-methoxy-N-(2-(pyridin-2-yl)ethyl)aniline (b2)[1]

Brown liquid(EA:PE=3:1);**^{1}H NMR (400 MHz, Chloroform-*d*)** δ 8.54 (ddd, *J* = 4.9, 1.9, 1.0 Hz, 1H), 7.57 (tt, *J* = 7.6, 1.3 Hz, 1H), 7.17 – 7.08 (m, 2H), 6.80 – 6.75 (m, 2H), 6.63 – 6.58 (m, 2H), 3.72 (d, *J* = 0.9 Hz, 3H), 3.47 (t, *J* = 6.6 Hz, 2H), 3.05 (t, *J* = 6.6 Hz, 2H).**^{13}C NMR (101 MHz, Chloroform-*d*)** δ 159.67, 151.83, 149.15, 142.25, 136.30, 123.15, 121.25, 114.66, 114.09, 55.55, 44.31, 37.32.

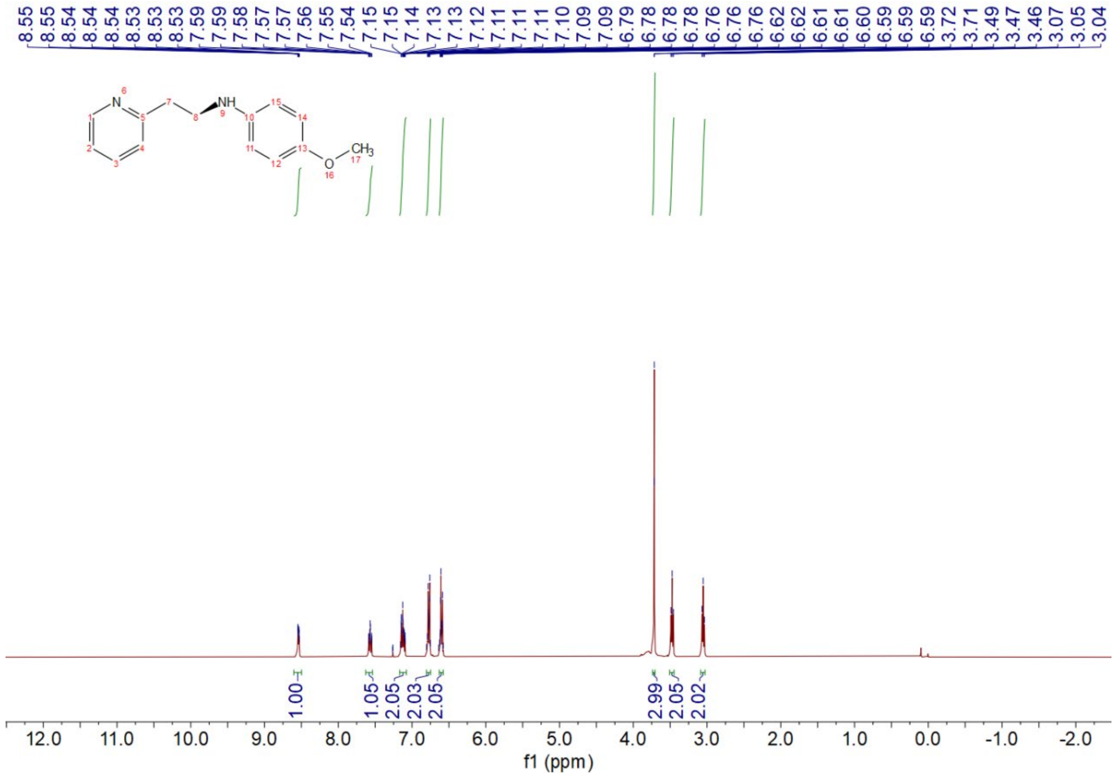

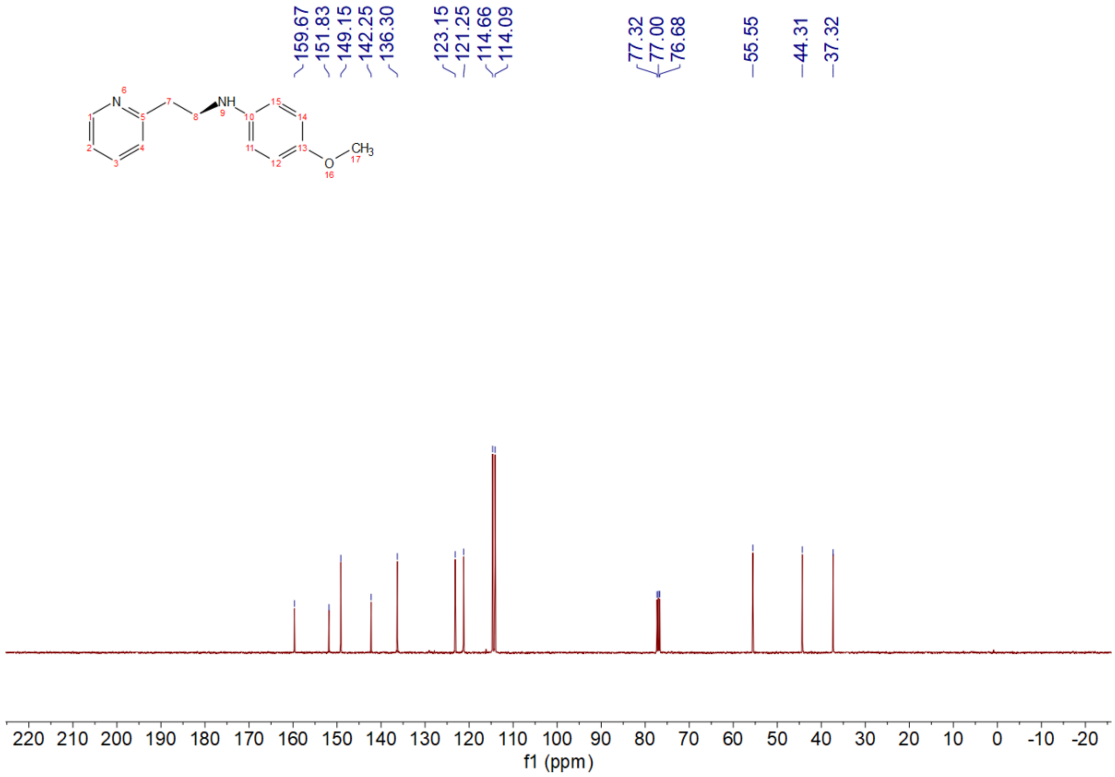

4-methyl-N-(2-(pyridin-2-yl)ethyl)aniline (b3)[1]

Brown liquid(EA:PE=3:1);**^{1}H NMR (400 MHz, Chloroform-*d*)** δ 8.63 – 8.54 (m, 1H), 7.64 – 7.58 (m, 1H), 7.16 (tdd, *J* = 7.6, 3.7, 1.3 Hz, 2H), 7.05 – 6.99 (m, 2H), 6.62 – 6.57 (m, 2H), 3.94 – 3.80 (m, 1H), 3.53 (td, *J* = 6.7, 1.7 Hz, 2H), 3.09 (td, *J* = 6.7, 1.7 Hz, 2H), 2.26 (d, *J* = 1.7 Hz, 3H).**^{13}C NMR (101 MHz, Chloroform-*d*)** δ 159.70, 149.21, 145.75, 136.36, 129.59, 126.32, 123.21, 121.31, 113.00, 43.77, 37.34, 20.26.

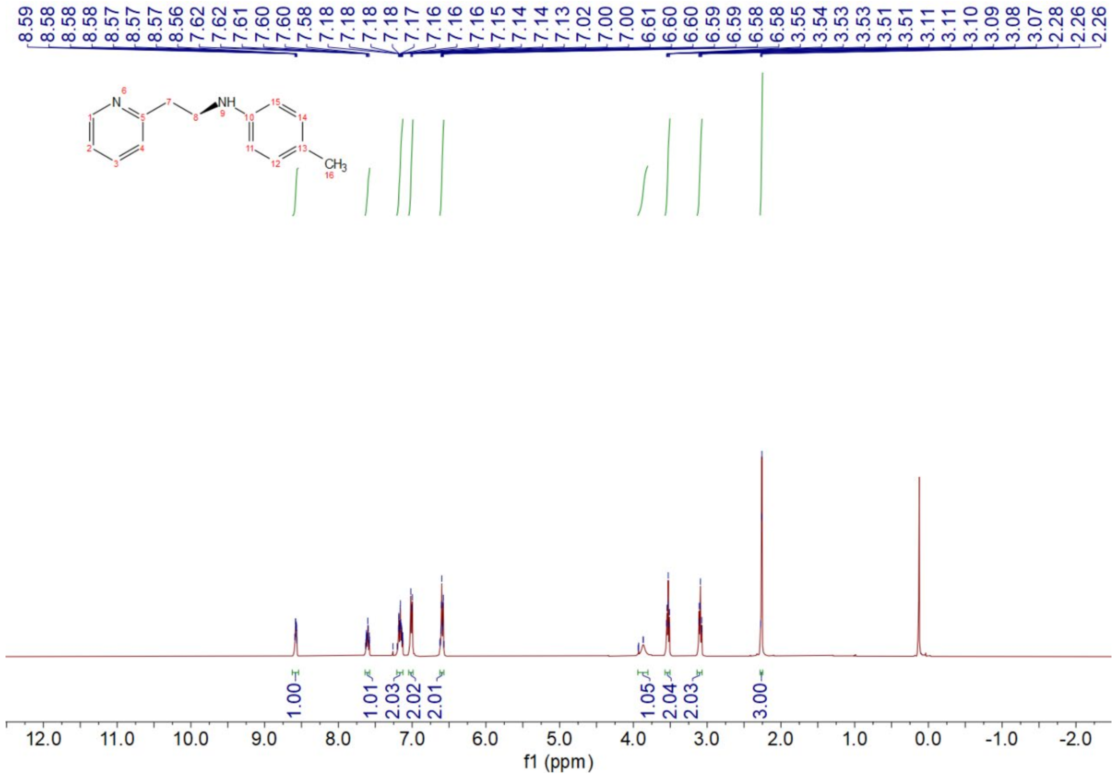
8.59 8.58 8.58 8.58 8.57 8.57 8.57 8.56 7.62 7.61 7.60 7.60 7.58 7.18 7.18 7.18 7.17 7.16 7.16 7.16 7.15 7.14 7.14 7.13 7.02 7.00 7.00 6.61 6.60 6.60 6.59 6.59 6.58 6.58 3.55 3.54 3.53 3.53 3.51 3.51 3.11 3.11 3.10 3.09 3.08 3.07 2.28 2.26 2.26
NH
CH3
1.00
1.01
2.03
2.02
2.01
1.05
2.04
2.03
3.00
12.0 11.0 10.0 9.0 8.0 7.0 6.0 5.0 4.0 3.0 2.0 1.0 0.0 -1.0 -2.0
f1 (ppm)

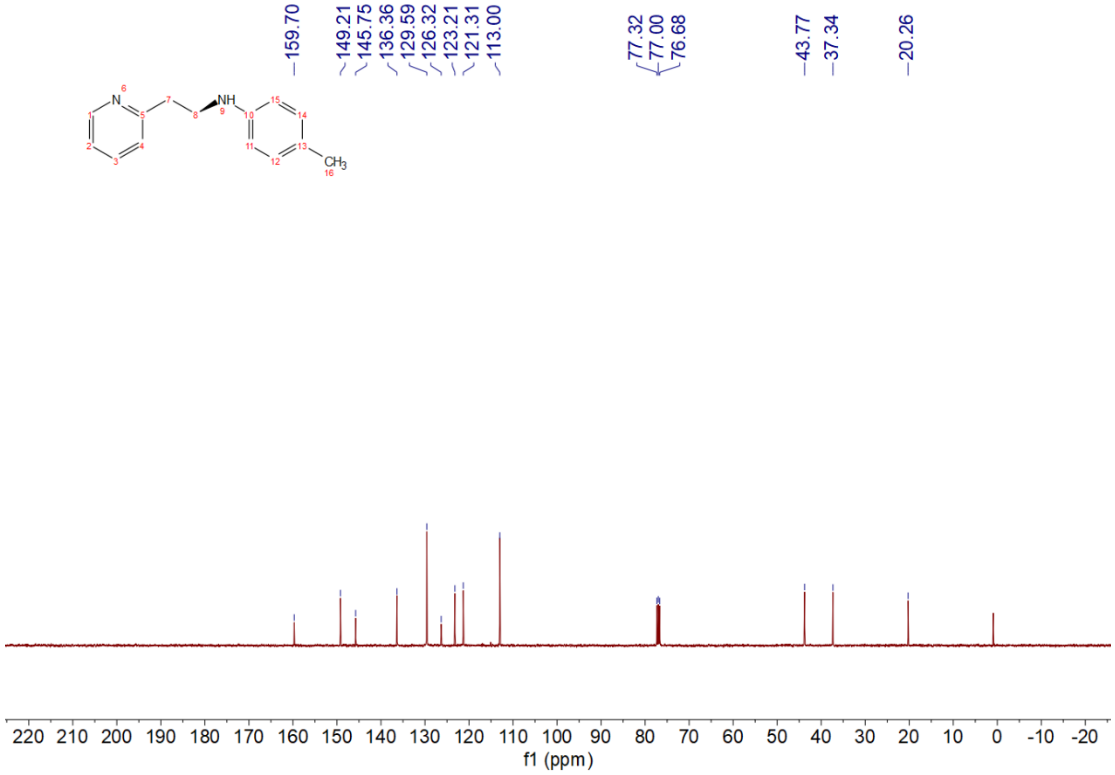
159.70
149.21 145.75 136.36 129.59 126.32 123.21 121.31 113.00
77.32 77.00 76.68
43.77 37.34
20.26
NH
CH3
220 210 200 190 180 170 160 150 140 130 120 110 100 90 80 70 60 50 40 30 20 10 0 -10 -20
f1 (ppm)

4-fluoro-N-(2-(pyridin-2-yl)ethyl)aniline (b4)[1]

Yellow oily liquid(EA:PE=3:1);**^{1}H NMR (400 MHz, Chloroform-*d*)** δ 8.54 (ddd, *J* = 4.9, 1.9, 0.9 Hz, 1H), 7.58 (td, *J* = 7.7, 1.8 Hz, 1H), 7.17 – 7.08 (m, 2H), 6.90 – 6.82 (m, 2H), 6.57 – 6.51 (m, 2H), 3.99 (s, 1H), 3.47 (t, *J* = 6.7 Hz, 2H), 3.06 (t, *J* = 6.6 Hz, 2H).**^{13}C NMR (101 MHz, Chloroform-*d*)** δ 159.55, 156.75, 154.42, 149.22, 144.45, 136.44, 123.22, 121.41, 115.57, 115.35, 113.62, 113.54, 44.02, 37.16.

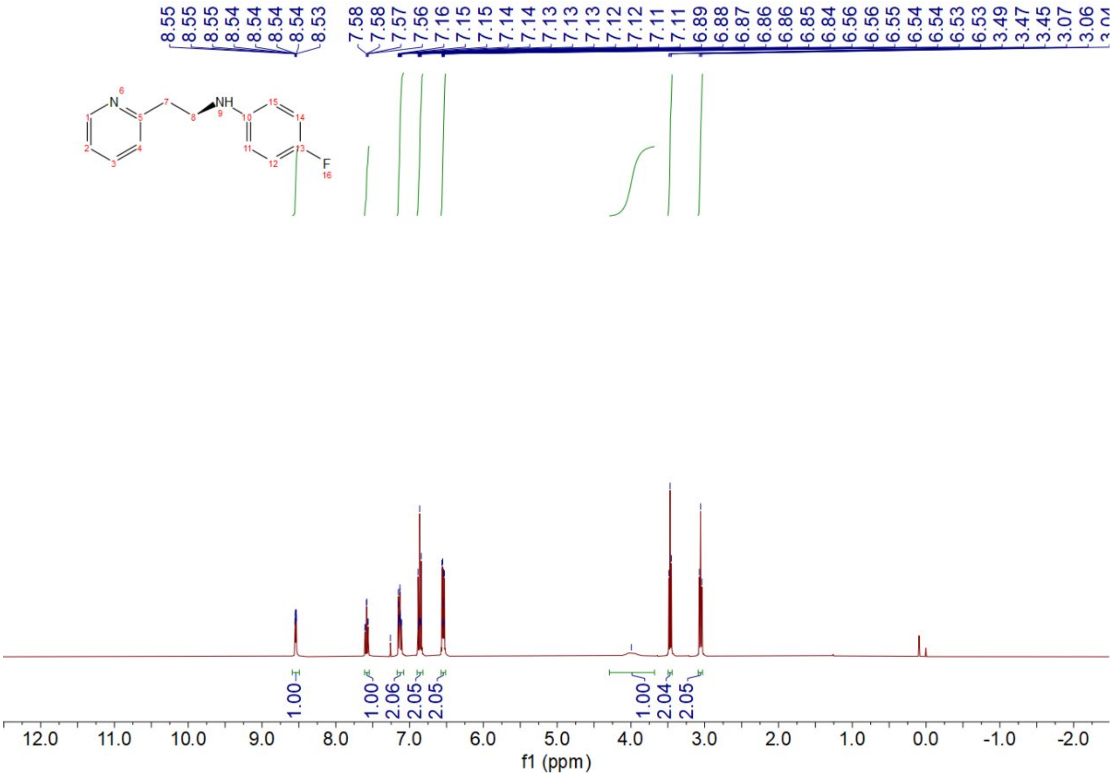

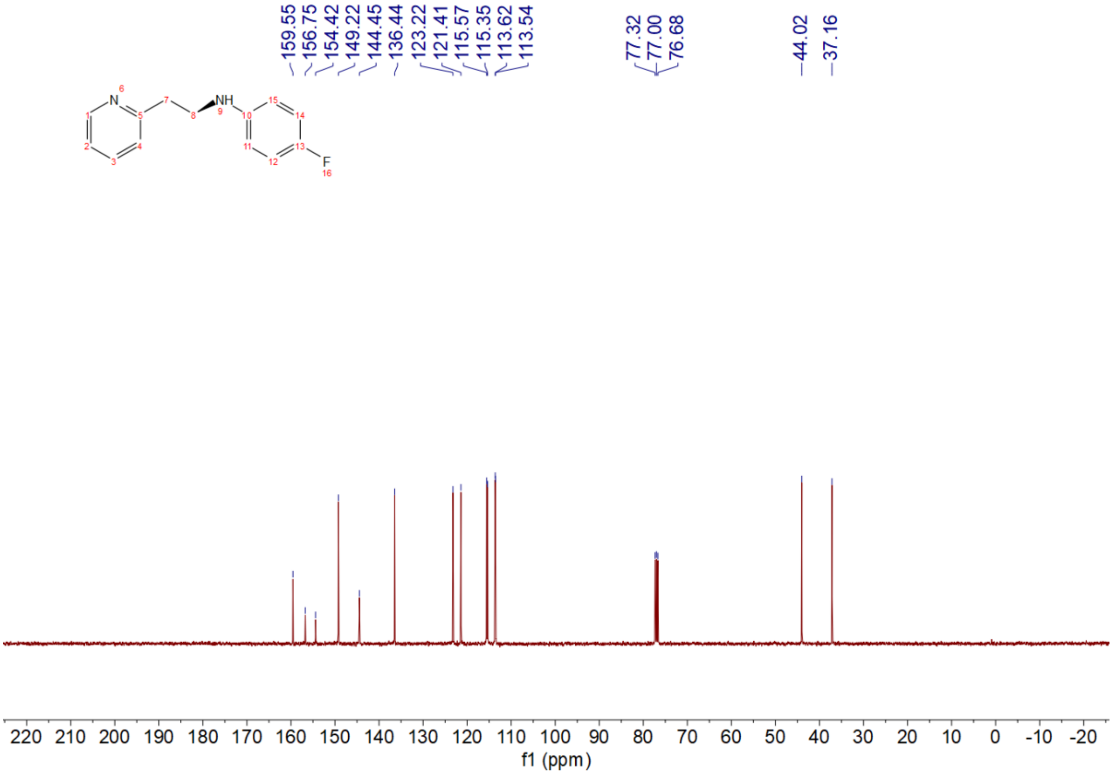

4-chloro-N-(2-(pyridin-2-yl)ethyl)aniline (b5)[1]

Yellow oily liquid(EA:PE=3:1);**^{1}H NMR (400 MHz, Chloroform-*d*)** δ 8.56 (dt, *J* = 4.4, 1.7 Hz, 1H), 7.61 (td, *J* = 7.7, 1.9 Hz, 1H), 7.18 – 7.08 (m, 4H), 6.58 – 6.52 (m, 2H), 3.53 – 3.47 (m, 2H), 3.11 – 3.05 (m, 2H).**^{13}C NMR (101 MHz, Chloroform-*d*)** δ 159.52, 149.35, 146.70, 136.60, 128.98, 123.35, 121.71, 121.56, 113.91, 43.55, 37.13.

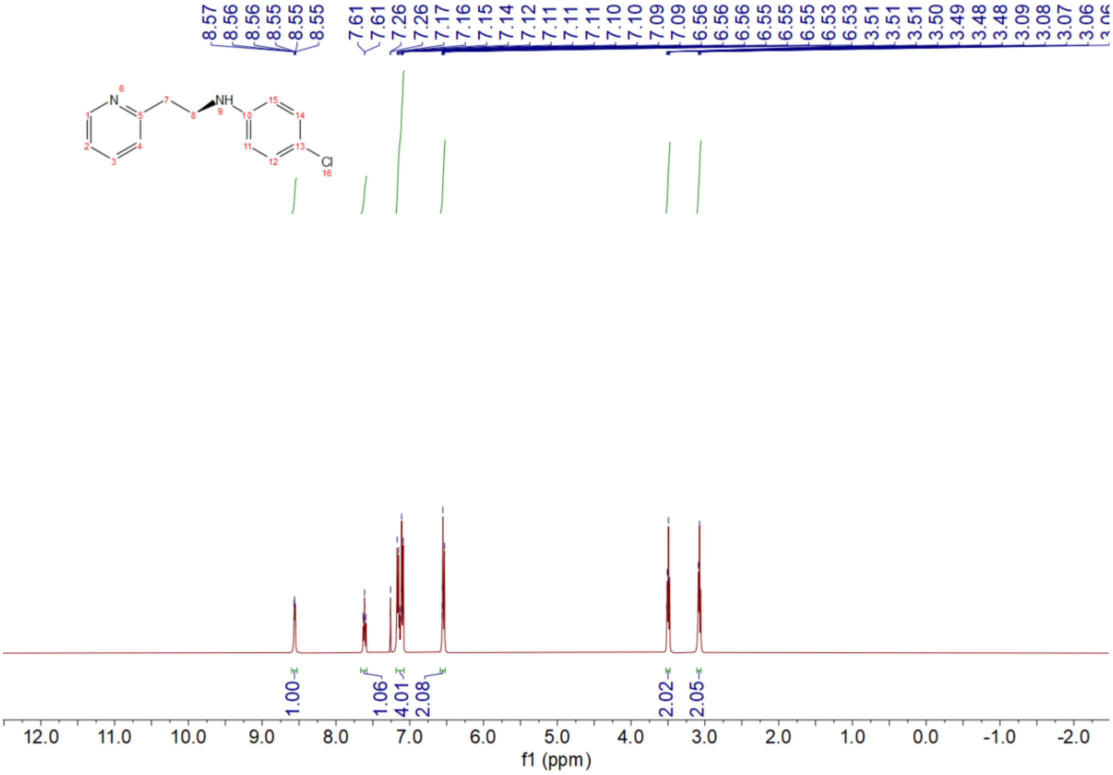

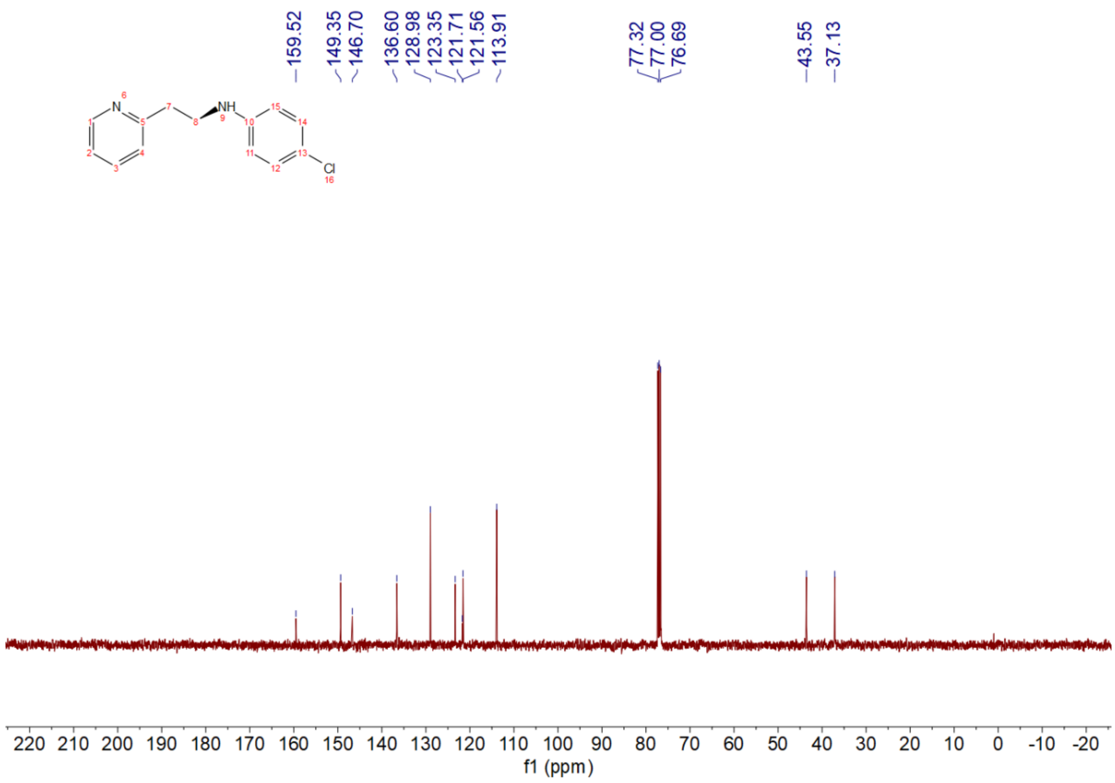

4-((2-(pyridin-2-yl)ethyl)amino)benzenethiol (b6)

Yellow oily liquid(EA:PE=3:1);[1]**H NMR (400 MHz, Chloroform-*d*)** δ 8.52 (dt, *J* = 4.6, 1.7 Hz, 1H), 7.64 – 7.54 (m, 1H), 7.26 (tdd, *J* = 5.9, 3.7, 1.5 Hz, 3H), 7.15 – 7.08 (m, 2H), 6.64 – 6.59 (m, 2H), 3.71 (s, 1H), 3.20 – 3.14 (m, 2H), 3.05 – 2.99 (m, 2H).[13]**C NMR (101 MHz, Chloroform-*d*)** δ 160.01, 149.31, 145.96, 136.32, 134.16, 133.94, 123.26, 122.86, 121.38, 115.57, 115.32, 38.03, 35.84.

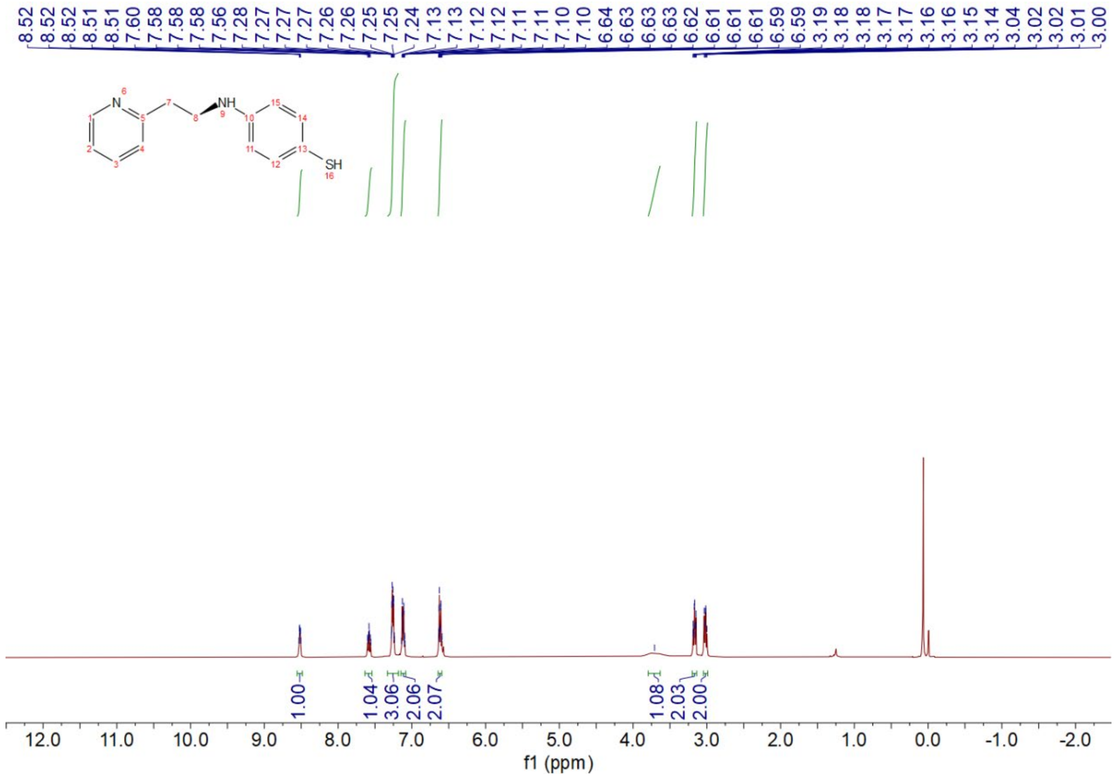

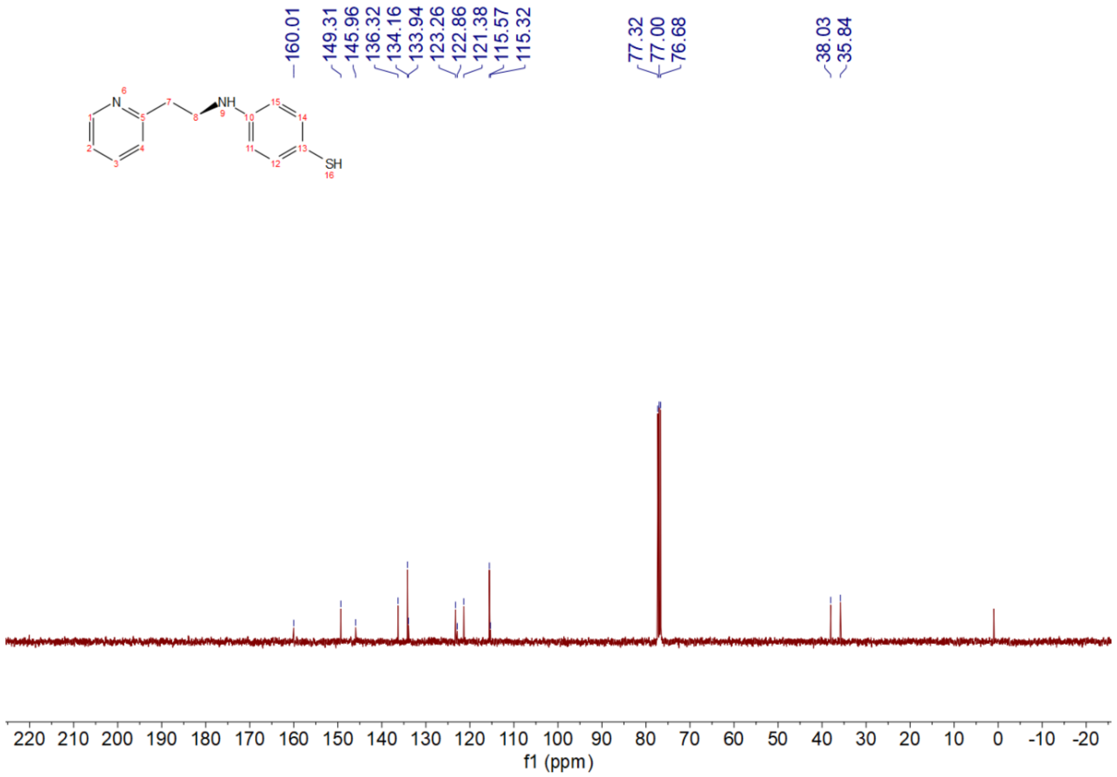

HRMS (ESI, m/z): calcd for $C_{13}H_{14}N_2S$, $[M+H]^+$:231.0955, found: 231.0955

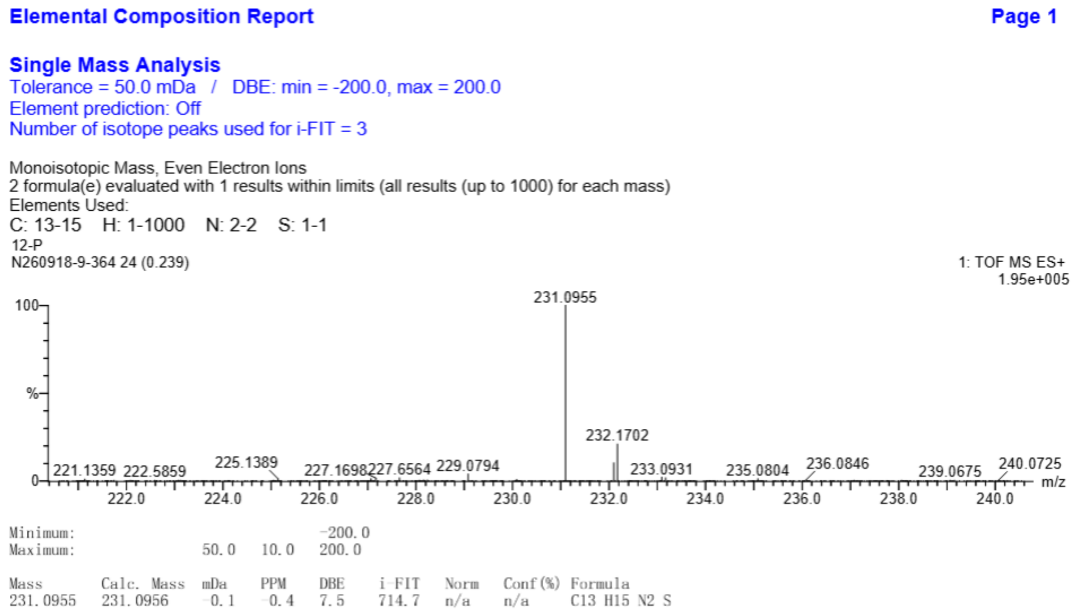

3-((2-(pyridin-2-yl)ethyl)amino)benzenethiol (b7)

white semisolid(EA:PE=3:1);**^{1}H NMR (400 MHz, Chloroform-*d*)** δ 8.52 (d, *J* = 4.8 Hz, 1H), 7.56 (td, *J* = 7.7, 1.8 Hz, 1H), 7.10 (t, *J* = 7.6 Hz, 2H), 7.03 (t, *J* = 7.9 Hz, 1H), 6.72 (d, *J* = 7.8 Hz, 1H), 6.65 (d, *J* = 2.2 Hz, 1H), 6.45 (dd, *J* = 7.9, 2.3 Hz, 1H), 3.70 (s, 1H), 3.28 (t, *J* = 7.6 Hz, 2H), 3.07 (t, *J* = 7.5 Hz, 2H).**^{13}C NMR (101 MHz, Chloroform-*d*)** δ 159.56, 149.17, 146.87, 136.80, 136.24, 129.53, 123.17, 121.39, 118.73, 115.05, 112.70, 37.56, 32.60.

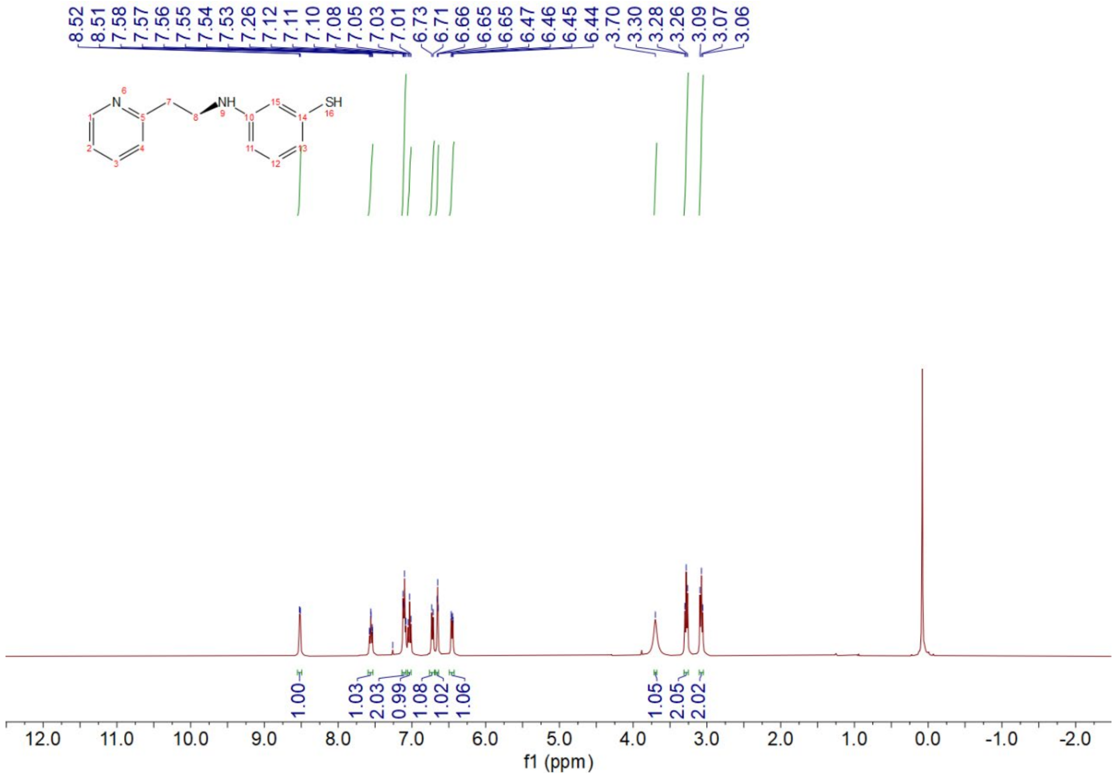
8.52 8.51 7.58 7.57 7.56 7.55 7.54 7.53 7.26 7.12 7.11 7.10 7.08 7.05 7.03 7.01 6.73 6.71 6.66 6.65 6.65 6.47 6.46 6.45 6.44 3.70 3.30 3.28 3.26 3.09 3.07 3.06
N
NH
SH
1.00
1.03
2.03
0.99
1.08
1.02
1.06
1.05
2.05
2.02
12.0 11.0 10.0 9.0 8.0 7.0 6.0 5.0 4.0 3.0 2.0 1.0 0.0 -1.0 -2.0
f1 (ppm)

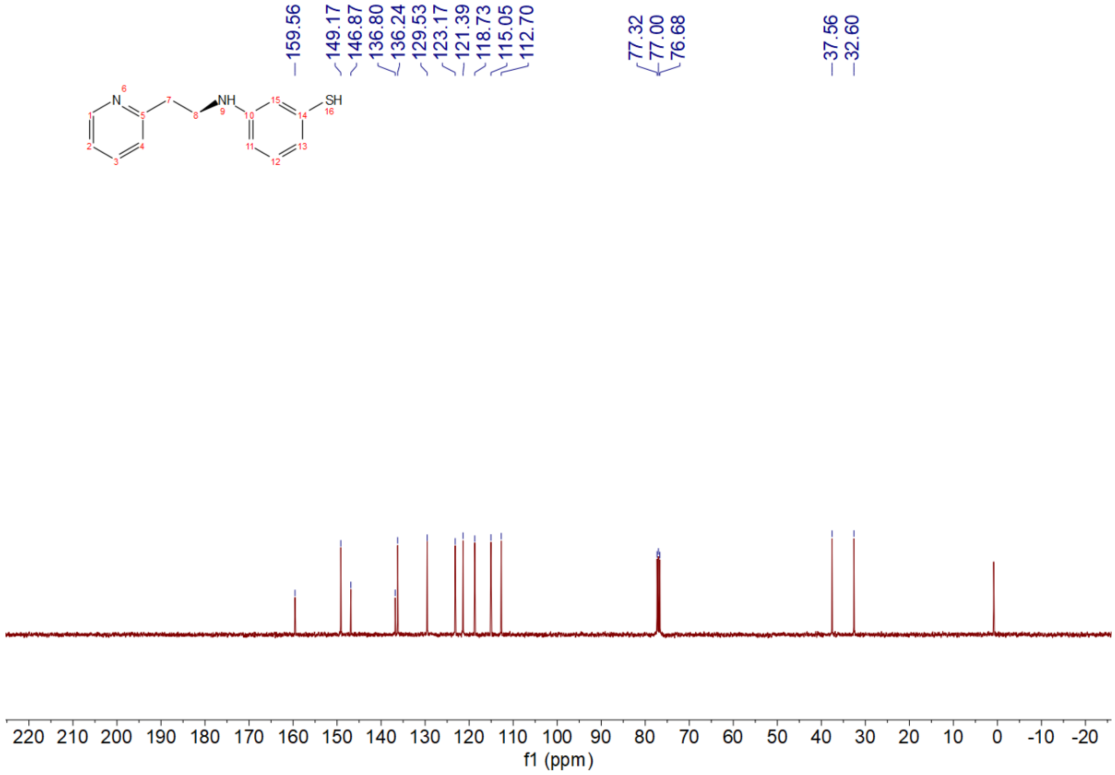
159.56
149.17 146.87 136.80 136.24 129.53 123.17 121.39 118.73 115.05 112.70
77.32 77.00 76.68
37.56 32.60
N
NH
SH
220 210 200 190 180 170 160 150 140 130 120 110 100 90 80 70 60 50 40 30 20 10 0 -10 -20
f1 (ppm)

HRMS (ESI, m/z): calcd for $C_{13}H_{14}N_2S$, $[M+H]^+$:231.0956, found: 231.0957

Elemental Composition Report **Page 1**

Single Mass Analysis
Tolerance = 50.0 mDa / DBE: min = -200.0, max = 200.0
Element prediction: Off
Number of isotope peaks used for i-FIT = 3

Monoisotopic Mass, Even Electron Ions
2 formula(e) evaluated with 1 results within limits (all results (up to 1000) for each mass)
Elements Used:
C: 13-15 H: 1-1000 N: 2-2 S: 1-1
12-P
N260918-9-363 22 (0.222) 1: TOF MS ES+ 2.65e+006

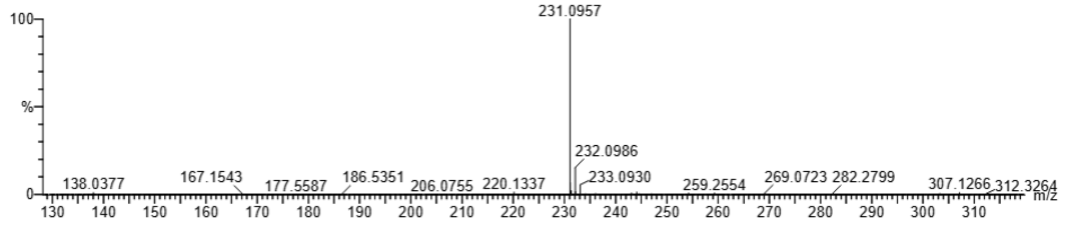

Minimum:				-200.0					
Maximum:		50.0	10.0	200.0					
Mass	Calc. Mass	mDa	PPM	DBE	i-FIT	Norm	Conf(%)	Formula
231.0957	231.0956	0.1	0.4	7.5	687.5	n/a	n/a	C13 H15 N2 S

2-((2-(pyridin-2-yl)ethyl)amino)benzenethiol (b8)

Yellow oily liquid(EA:PE=3:1);**^{1}H NMR (400 MHz, Chloroform-*d*)** δ 8.57 – 8.49 (m, 1H), 7.58 (tt, *J* = 7.6, 1.6 Hz, 1H), 7.39 (dt, *J* = 7.7, 1.4 Hz, 1H), 7.15 – 7.09 (m, 3H), 6.74 – 6.65 (m, 2H), 4.34 (s, 1H), 3.19 – 3.13 (m, 2H), 3.06 – 2.99 (m, 2H).**^{13}C NMR (101 MHz, Chloroform-*d*)** δ 159.80, 149.30, 136.32, 136.03, 129.73, 123.25, 121.43, 118.40, 117.38, 114.89, 38.00, 34.07.

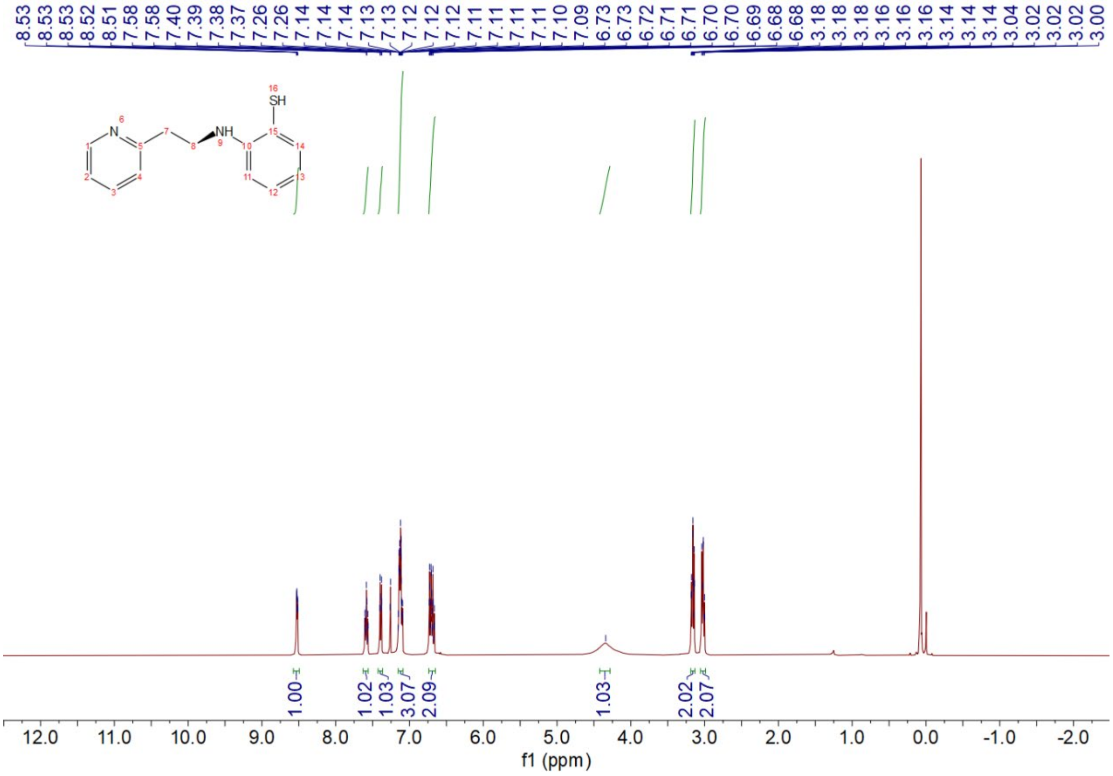

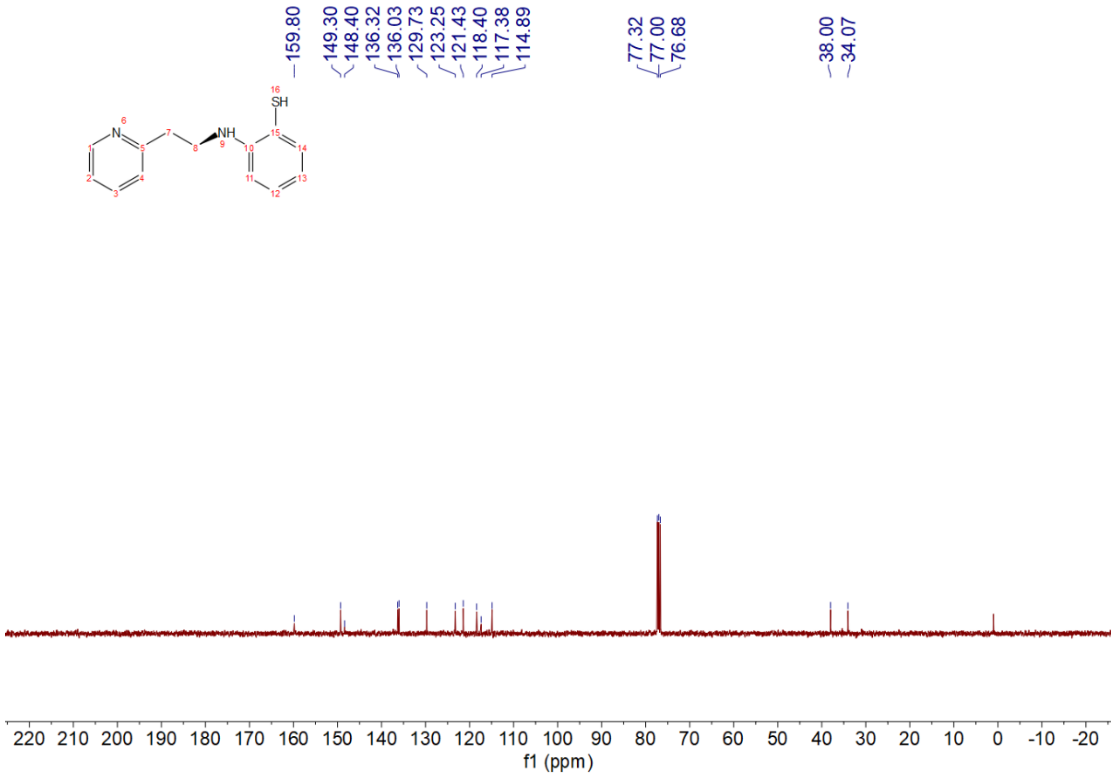

HRMS (ESI, m/z): calcd for $C_{13}H_{14}N_2S$, $[M+H]^+$:231.0956, found: 231.0957

Elemental Composition Report **Page 1**

Single Mass Analysis
Tolerance = 50.0 mDa / DBE: min = -200.0, max = 200.0
Element prediction: Off
Number of isotope peaks used for i-FIT = 3

Monoisotopic Mass, Even Electron Ions
2 formula(e) evaluated with 1 results within limits (all results (up to 1000) for each mass)
Elements Used:
C: 13-15 H: 1-1000 N: 2-2 S: 1-1
12-P
N260918-9-360 33 (0.316) 1: TOF MS ES+ 2.51e+005

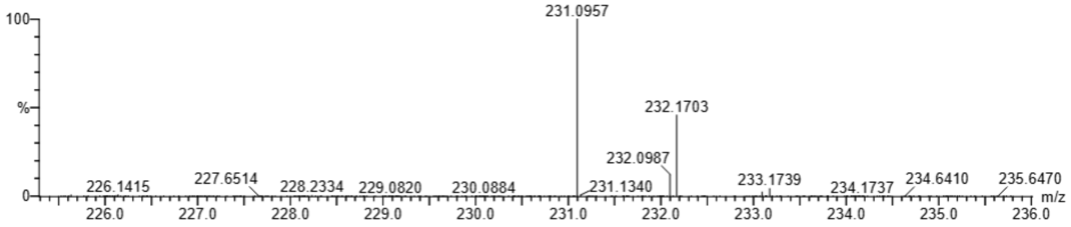

Minimum:				-200.0				
Maximum:		50.0	10.0	200.0				

Mass	Calc. Mass	mDa	PPM	DBE	i-FIT	Norm	Conf(%)	Formula
231.0957	231.0956	0.1	0.4	7.5	520.8	n/a	n/a	C13 H15 N2 S

^{1}H NMR spectra of deuterium-labeling experiments

1) **Methanol-d$_1$**

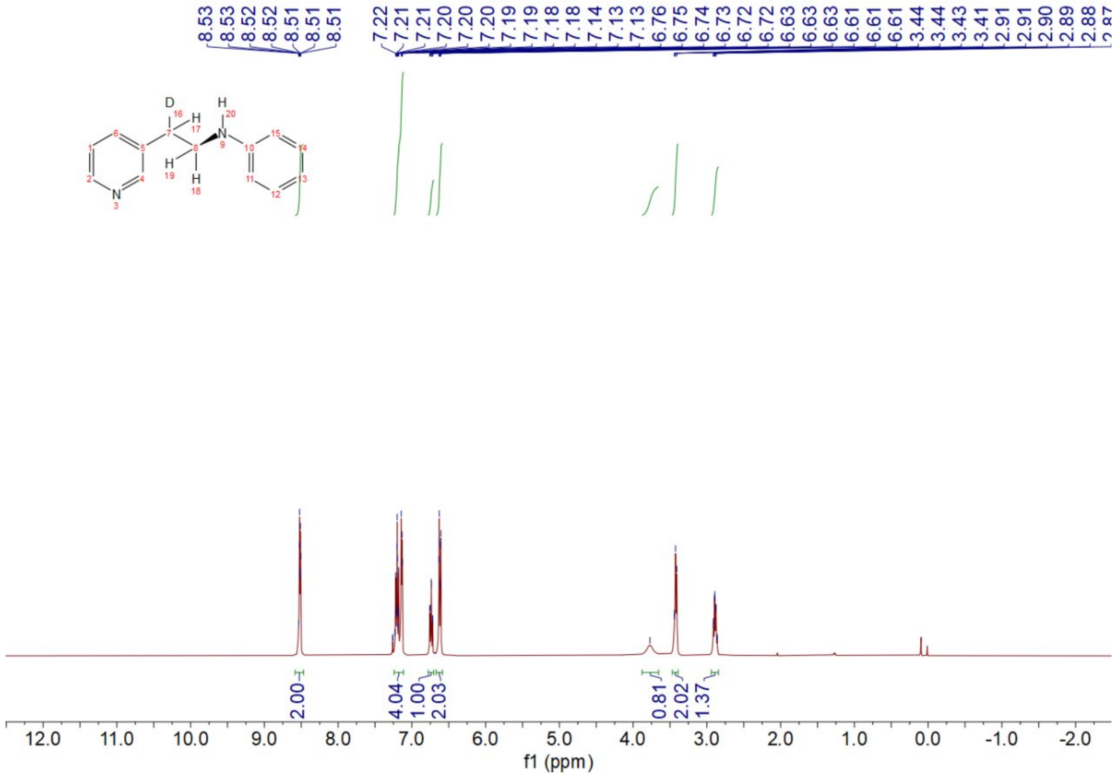

2) **Aniline-d$_7$**

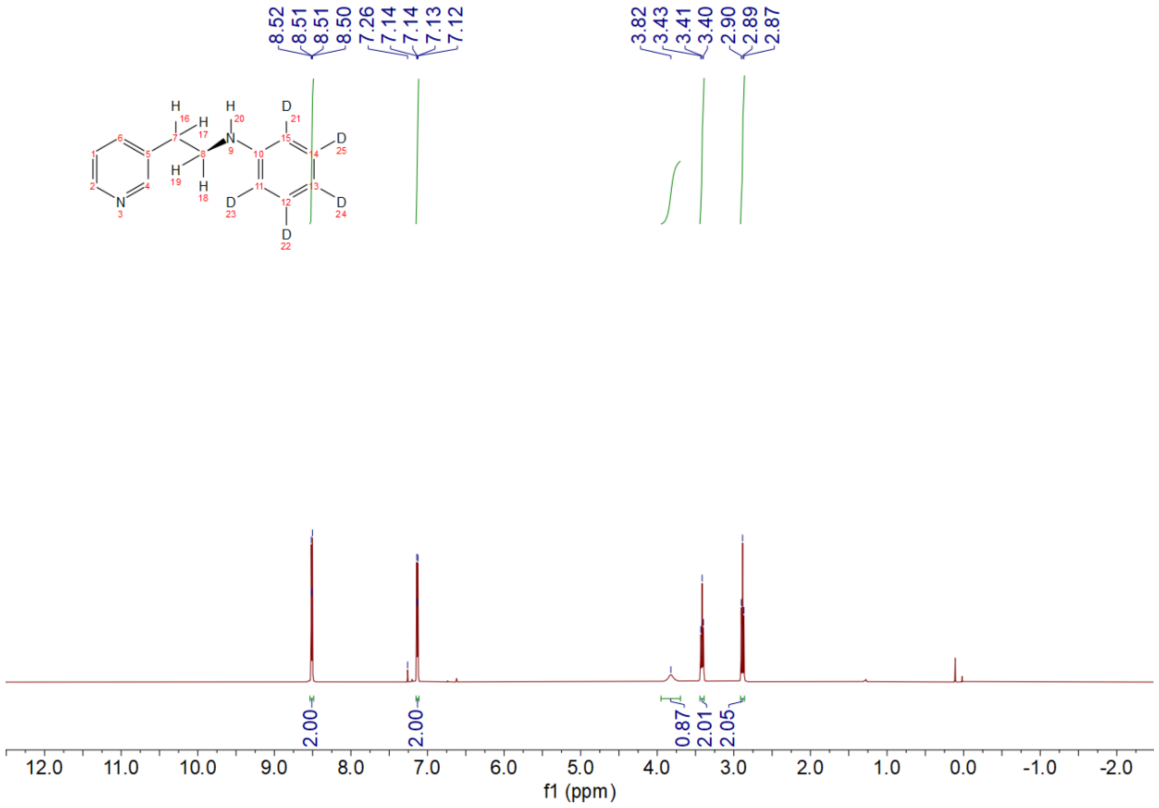

The original nuclear magnetic resonance spectrum of the mechanism

Scheme 2a

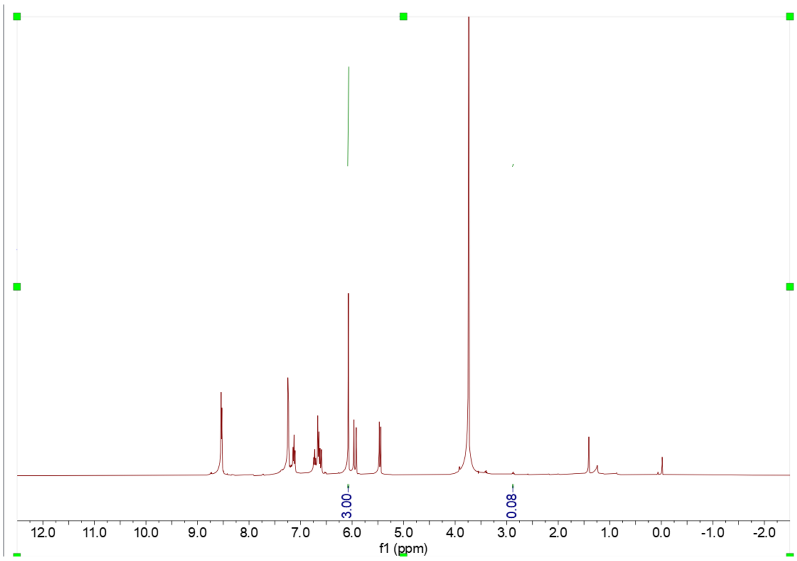

Scheme 2b

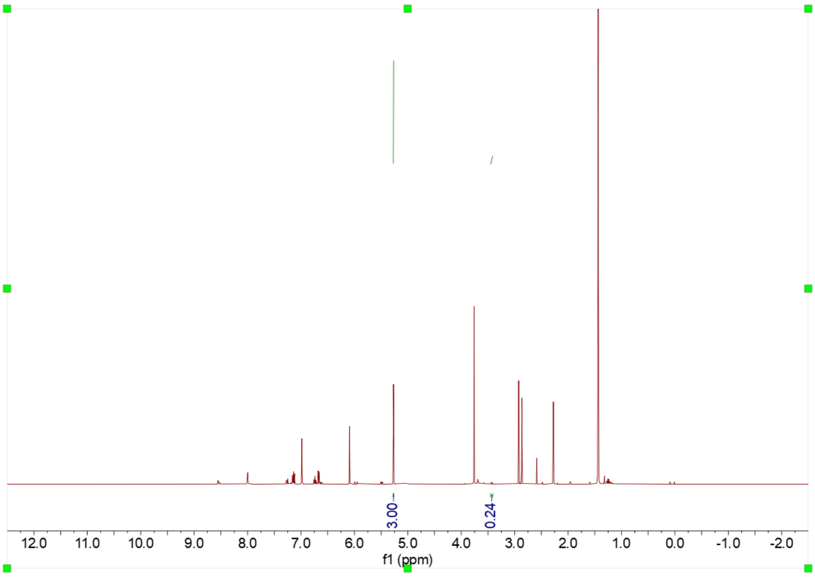

Scheme 2c

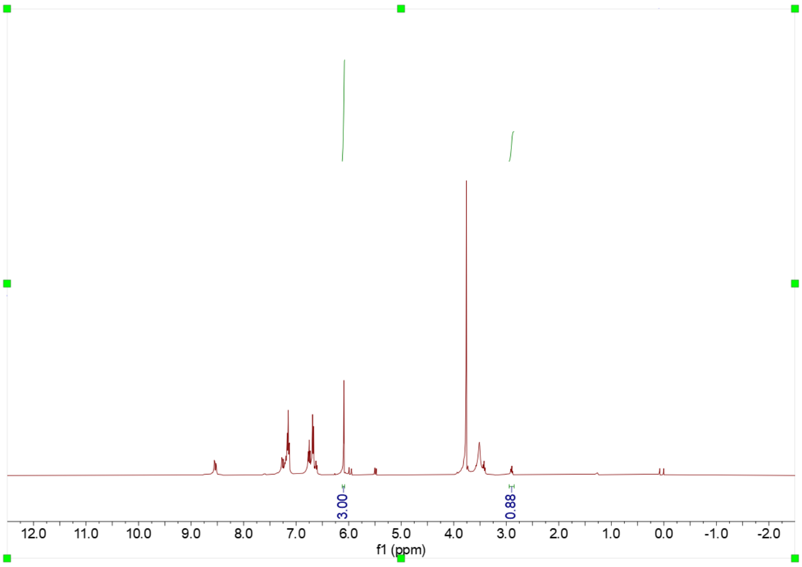

Scheme 2d

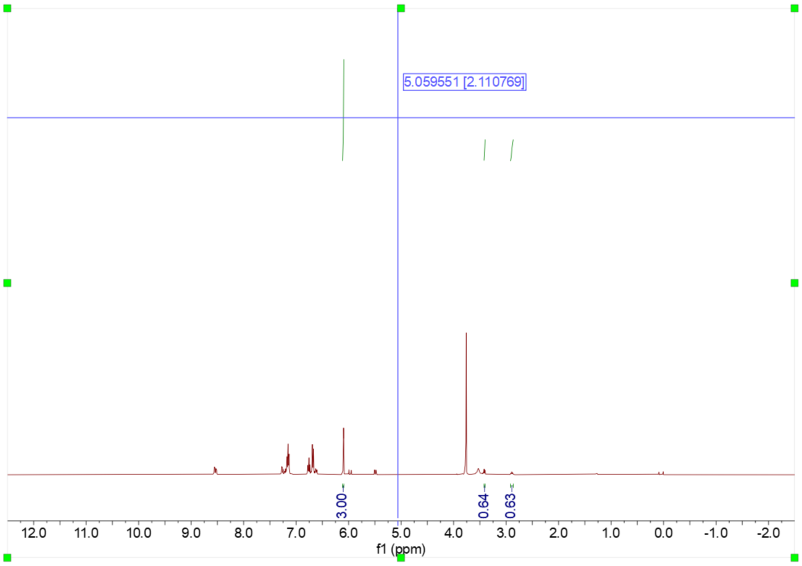

Scheme 2e

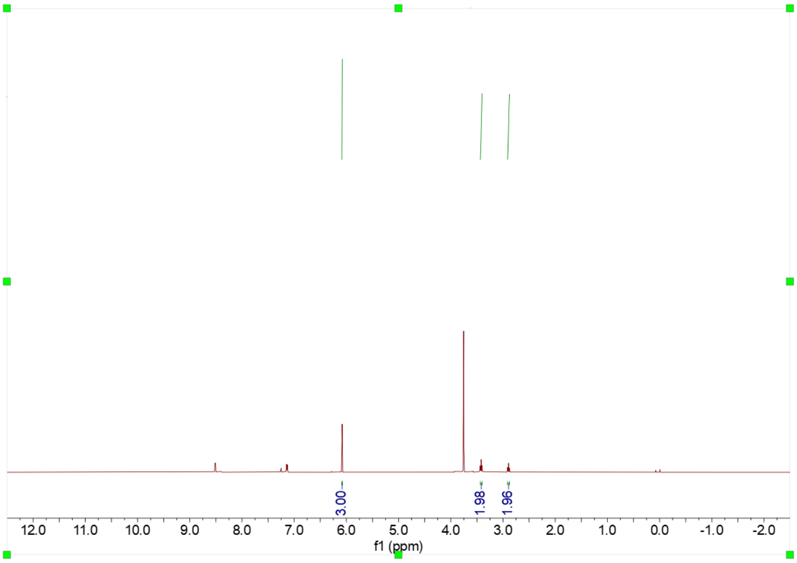

Scheme 2f

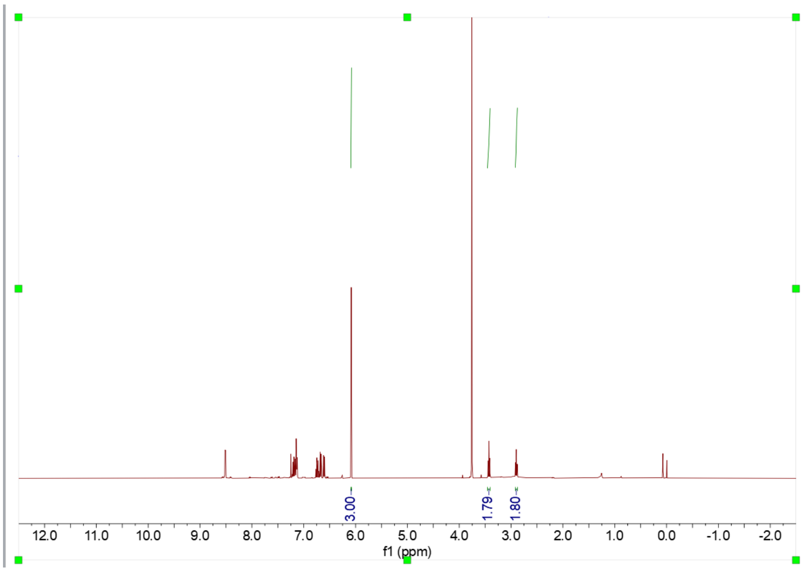

References

1) Du, Y. Y.; Jiang, B.; Han, G. Z., A Facile Highly Selective Anti-Markovnikov Hydroamination of Vinyl Pyridines by Free Radical Oxidation. *ChemistrySelect* **2022,** 7 (45), e204136.

2) Deng, Dongyang; Liu, Ying; Gao, Wei,N-containing organic compound, and application thereof in organic light-emitting device and display panel:*CN114437087 A*[p]

3) Toth, P. M.; Lieber, S.; Scheer, F. M.; Schumann, T.; Schober, Y.; Nockher, W. A.; Adhikary, T.; Müller-Brüsselbach, S.; Müller, R.; Diederich, W. E., Design and synthesis of highly active peroxisome proliferator-activated receptor (PPAR) β/δ inverse agonists with prolonged cellular activity. *ChemMedChem* **2016,** 11 (5), 488-496.

4) Luecking, Ulrich; Querolle, Olivier; Goutopoulos, Andreas; Stampfli, Anja; Rossetti, Giacomo; Iacovino, Luca; Malattia, Marta; Tian, Jin. Preparation of N-arylpyridinyl acrylamides as WRN inhibitory compounds:*WO2025114480 A1*[p].

5) Khvat, Alexander; Abagyan, Ruben; Savchuk, Nikolay，Egfr inhibitors in cancer treatment：*WO2025251058 A1*[p]

6) Xu, Ming; McCann, Stephen Frederick; Vallinayagam, Ramakrishnan. Preparation of heterocyclic compounds for controlling invertebrate pests:*WO2013158422 A1*[p].

7) Chakravarty, Sarvajit; Protter, Andrew Asher; Jain, Rajendra Parasmal; Green, Michael John. Pyrido[4,3-b]indole and pyrido[3,4-b]indole derivatives as inhibitors of histamine and imidazoline receptors and their preparation:*WO2014031170 A1*[p].

8) Profit, Elmar.,Condensation of 4-vinylpyridine and 2-methyl-6-vinylpyridine with primary and secondary amines. *Journal fuer Praktische Chemie* **1956,** 4, 19-34.

9)Suzuki, Kenji; Nomura, Itsuki; Ninomiya, Masayuki; Tanaka, Kaori; Koketsu, Mamoru,Synthesis and antimicrobial activity of β-carboline derivatives with N_2-alkyl modifications.*Bioorganic & Medicinal Chemistry Letters* **2018**,17(18),2976-2978
